# Supplementary material for: Could changing invitation and booking processes help women translate their cervical screening intentions into action? A population-based survey of women’s preferences in Great Britain
Source: BMJ Open. 2019 Jul 11;9(7):e028134. doi: 10.1136/bmjopen-2018-028134 (PMC6629419; doi:10.1136/bmjopen-2018-028134)
Supplement: Supplementary file 1 [file bmjopen-2018-028134supp001.pdf]

Online Supplement 1: Could changing invitation and booking processes help women translate their cervical screening intentions into action? A population-based survey of women's preferences in Great Britain.

(Mairead Ryan, Jo Waller and Laura Marlow)

Questionnaire

Have you ever been diagnosed with cervical cancer?

1 Yes

2 No

The next few questions in this section are about cervical screening, also known as a smear or a Pap test. The NHS Cervical Screening Programme invites women in England for a cervical screening, smear or Pap test every 3 years from age 25 to age 49 and every 5 years from age 50 to age 64. Which of these statements describes whether you have had a cervical screening? If you have had a cervical screening and can't remember when, please give your best estimate.

1 I have had a test within the last 3 years > INCLUDE (1)

2 My last test was 3 to 5 years ago > INCLUDE (2)

3 My last test was more than 5 years ago > INCLUDE (3)

4 I have never been invited to have a test > EXCLUDE

5 I have been invited but have never had a test > INCLUDE (4)

6 I have had a hysterectomy so I don't need to have tests > EXCLUDE

7 I have never heard of cervical screening > EXCLUDE

Will you go for cervical screening when next invited?

1 Definitely not > EXCLUDE

2 Probably not > EXCLUDE

3 Yes, probably > INCLUDE (a)

4 Yes, definitely > INCLUDE (a)

NB: Participants were categorised as follows based on responses to the above questions:

If answered 1 and a = maintainer

If 25-49 years and answered 2 and a = intender

If 50-64 and answered 2 and a = maintainer

If answered 3 or 4 and a = intender

On the next screen will be an invitation letter that the NHS sends to women to invite them to book a cervical screening appointment. Most women book cervical screening appointments at their GP practice. I would like you to imagine you received this letter in the post. Please read the letter and afterwards you will be asked some questions about your response to the letter.

* Picture of NHS screening letter shown to participant

I will now read a number of statements relating to the cervical screening letter you've just read. After each statement, please state the extent to which you agree, on a scale from 'strongly disagree 'to 'strongly agree'.

How much do you agree or disagree with this statement?
It is easy for me to find time to read a letter like this.

1 Strongly disagree
2 Disagree
3 Neither disagree or agree
4 Agree
5 Strongly agree

How much do you agree or disagree with this statement?
I might forget to book an appointment after reading this letter.

1 Strongly disagree
2 Disagree
3 Neither disagree or agree
4 Agree
5 Strongly agree

How much do you agree or disagree with this statement?
It is difficult for me to call my GP practice during their opening hours.
*GP opening hours provided if necessary: "Opening hours are generally between 8.00am to 6.30pm Monday to Friday"

1 Strongly disagree
2 Disagree
3 Neither disagree or agree
4 Agree
5 Strongly agree

How much do you agree or disagree with this statement?
I have access to a telephone/mobile with phone credit/minutes to call my GP practice.

1 Strongly disagree
2 Disagree
3 Neither disagree or agree
4 Agree
5 Strongly agree

How much do you agree or disagree with this statement?
It would be easy for me to find the phone number for my GP practice to contact them.

1 Strongly disagree
2 Disagree
3 Neither disagree or agree
4 Agree
5 Strongly agree

How much do you agree or disagree with this statement?
I find it takes too long to get through to a receptionist when I phone my GP practice.

1 Strongly disagree
2 Disagree
3 Neither disagree or agree
4 Agree
5 Strongly agree

We are interested in what is important to you in terms of booking a cervical screening appointment. For the following statements I read out, please state the extent to which you think each factor is important to you, on a scale from 'very unimportant' to 'very important' when booking an appointment at your GP practice.

How important is this when booking a cervical screening appointment at your GP practice?
Ease of booking

1 Very unimportant
2 Quite unimportant
3 Neither unimportant or important
4 Quite important
5 Very important

How important is this when booking a cervical screening appointment at your GP practice?
Cost of making booking (i.e. phone credit)

1 Very unimportant
2 Quite unimportant
3 Neither unimportant or important
4 Quite important
5 Very important

How important is this when booking a cervical screening appointment at your GP practice?
Choice of appointment times

1 Very unimportant
2 Quite unimportant
3 Neither unimportant or important
4 Quite important
5 Very important

How important is this when booking a cervical screening appointment at your GP practice?
Being able to change an appointment time/day after booking it

1 Very unimportant
2 Quite unimportant
3 Neither unimportant or important
4 Quite important
5 Very important

How important is this when booking a cervical screening appointment at your GP practice?
Privacy when booking an appointment

1 Very unimportant
2 Quite unimportant
3 Neither unimportant or important
4 Quite important
5 Very important

How important is this when booking a cervical screening appointment at your GP practice?
How long it takes to book an appointment

1 Very unimportant
2 Quite unimportant
3 Neither unimportant or important
4 Quite important
5 Very important

How important is this when booking a cervical screening appointment at your GP practice?
Being able to talk with a healthcare professional when booking (e.g. to ask questions about the screening before attending)

1 Very unimportant
2 Quite unimportant
3 Neither unimportant or important
4 Quite important
5 Very important

How important is this when booking a cervical screening appointment at your GP practice?
Time to the next available appointment (e.g. next available appointment isn't for two weeks)

1 Very unimportant
2 Quite unimportant
3 Neither unimportant or important
4 Quite important
5 Very important

How important is this when booking a cervical screening appointment at your GP practice?
Being able to book an appointment when the GP practice is shut (e.g. online booking)
*GP opening hours provided if necessary: "Opening hours are generally between 8.00am to 6.30pm Monday to Friday"

1 Very unimportant
2 Quite unimportant
3 Neither unimportant or important
4 Quite important
5 Very important

Again thinking about the letter you read which is sent in the post to invite women to book a cervical screening appointment. We are interested in different forms of communication to invite women to book a cervical screening appointment.

Please state the extent to which you think the following forms of communication are acceptable, on a scale from 'very unacceptable' to 'very acceptable'.

How acceptable is this form of communication when being invited to book a cervical screening appointment?
Posted letter

1 Very unacceptable
2 Quite unacceptable
3 Neither unacceptable or acceptable
4 Quite acceptable
5 Very acceptable

* If participant responded ‘Quite unacceptable’ or ‘Very unacceptable’, participant subsequently asked: Please can you tell me why you would not find receiving an invitation for a cervical screening appointment by posted letter acceptable?
How acceptable is this form of communication when being invited to book a cervical screening appointment?
Text message

1 Very unacceptable
2 Quite unacceptable
3 Neither unacceptable or acceptable
4 Quite acceptable
5 Very acceptable

* If participant responded ‘Quite unacceptable’ or ‘Very unacceptable’, participant subsequently asked: Please can you tell me why you would not find receiving an invitation for a cervical screening appointment by text message acceptable?

How acceptable is this form of communication when being invited to book a cervical screening appointment?
Email

1 Very unacceptable
2 Quite unacceptable
3 Neither unacceptable or acceptable
4 Quite acceptable
5 Very acceptable

* If participant responded ‘Quite unacceptable’ or ‘Very unacceptable’, participant subsequently asked: Please can you tell me why you would not find receiving an invitation for a cervical screening appointment by email acceptable?

How acceptable is this form of communication when being invited to book a cervical screening appointment?
Phone call to your mobile phone

1 Very unacceptable
2 Quite unacceptable
3 Neither unacceptable or acceptable
4 Quite acceptable
5 Very acceptable

* If participant responded ‘Quite unacceptable’ or ‘Very unacceptable’, participant subsequently asked: Please can you tell me why you would not find receiving an invitation for a cervical screening appointment by phone call to your mobile phone acceptable?

How acceptable is this form of communication when being invited to book a cervical screening appointment?
Phone call to your house landline

1 Very unacceptable
2 Quite unacceptable
3 Neither unacceptable or acceptable
4 Quite acceptable
5 Very acceptable

* If participant responded ‘Quite unacceptable’ or ‘Very unacceptable’, participant subsequently asked: Please can you tell me why you would not find receiving an invitation for a cervical screening appointment by phone call to your house landline acceptable?

Imagine now that different options were available to you to book a cervical screening appointment at your GP practice. Please state the extent to which you are likely to use each of the following methods to book an appointment.

How likely are you to use this method to book a cervical screening appointment at your GP practice?
Calling your GP practice

1 Very unlikely
2 Quite unlikely
3 Neither likely or unlikely
4 Quite likely
5 Very likely

How likely are you to use this method to book a cervical screening appointment at your GP practice?
Calling a 24-hour automated telephone appointment-booking system

1 Very unlikely
2 Quite unlikely
3 Neither likely or unlikely
4 Quite likely
5 Very likely

How likely are you to use this method to book a cervical screening appointment at your GP practice?
Requesting a call-back from your GP practice

1 Very unlikely
2 Quite unlikely
3 Neither likely or unlikely
4 Quite likely
5 Very likely

How likely are you to use this method to book a cervical screening appointment at your GP practice?
Booking on a website using a desktop computer/laptop

1 Very unlikely
2 Quite unlikely
3 Neither likely or unlikely
4 Quite likely
5 Very likely

How likely are you to use this method to book a cervical screening appointment at your GP practice?
Booking on a website using a smartphone

1 Very unlikely
2 Quite unlikely
3 Neither likely or unlikely
4 Quite likely
5 Very likely

How likely are you to use this method to book a cervical screening appointment at your GP practice?
Downloading an app to a smartphone to book an appointment (you could then use the app to book other appointments at your surgery)

1 Very unlikely
2 Quite unlikely
3 Neither likely or unlikely
4 Quite likely
5 Very likely

Which of the following methods have you previously used to book an appointment at your GP practice? This could be an appointment for anything, with a GP or with a nurse.
Please select all that apply.

1 Booked in person (i.e. at the reception desk)
2 Booked by phoning the GP practice
3 Booked using a 24-hour automated telephone appointment-booking system
4 Booked online on a website
5 Booked by text-message
6 Booked using a smartphone app
7 Other
8 Don’t know - someone else has always booked my appointments
9 I have never booked an appointment at my GP practice

Do you have a mobile phone?
*Description of smartphone provided if necessary; “A ‘smart phone’ is a mobile phone that performs many of the functions of a computer, typically having a touchscreen and Internet access”

1 Yes, a smart phone
2 Yes, but it is not a smart phone
3 No, I do not have a mobile phone
