# Supplementary material for: Could changing invitation and booking processes help women translate their cervical screening intentions into action? A population-based survey of women’s preferences in Great Britain
Source: BMJ Open. 2019 Jul 11;9(7):e028134. doi: 10.1136/bmjopen-2018-028134 (PMC6629419; doi:10.1136/bmjopen-2018-028134)
Supplement: Supplementary file 2 [file bmjopen-2018-028134supp002.pdf]

Online Supplement 2: Could changing invitation and booking processes help women translate their cervical screening intentions into action? A population-based survey of women's preferences in Great Britain.

(Mairead Ryan, Jo Waller and Laura Marlow)

Table S1:

Descriptives for acceptability of cervical screening invitation modalities (n=614)

	**Very unacceptable/Quite unacceptable** N (%)	**Neither unacceptable or acceptable** N (%)	**Quite acceptable/Very acceptable** N (%)	**Don't know/ not applicable (Excluded)** N (%)
Posted letter	14 (2.3)	31 (5.0)	90.1 (90.1)	16 (2.6)
Text-message	65 (10.6)	50 (8.1)	482 (78.5)	17 (2.8)
Email	95 (15.5)	52 (8.5)	445 (72.5)	22 (3.6)
Mobile phone call	92 (15.0)	53 (8.6)	453 (73.8)	16 (2.6)
Landline phone call	132 (21.5)	85 (13.8)	359 (58.5)	38 (6.2)

Table S2:

Descriptives for likelihood of using different phone-based and online booking methods (n=614)

	**Very unlikely/Quite unlikely** N (%)	**Neither unlikely or likely** N (%)	**Quite likely/Very likely** N (%)	**Don't know/ not applicable (Excluded)** N (%)
Calling the GP	21 (3.4)	25 (4.1)	550 (89.6)	18 (2.9)
Calling a 24-hour automated service	182 (29.6)	91 (14.8)	317 (51.6)	24 (3.9)
Requesting a call-back	164 (26.7)	79 (12.9)	350 (57.0)	21 (3.4)
Booking on a website using a desktop/laptop	172 (28.0)	62 (10.1)	355 (57.8)	25 (4.1)
Booking on a website using a smartphone[a]	141 (26.5)	44 (8.3)	328 (61.5)	20 (3.8)
Downloading an app to your smartphone[a]	185 (34.7)	56 (10.5)	276 (51.8)	16 (3.0)

[a] participants with no smartphone removed from analyses (*n* = 81)

Table S3

Multivariable logistic regression models of predictors of the acceptability of cervical screening invitation modalities

	**Posted letter (n=597)**	**Text-message (n=596)**	**Email (n=591)**	**Mobile phone call (n=597)**	**Landline phone call (n=575)**
	OR (95% CI)	OR (95% CI)	OR (95% CI)	OR (95% CI)	OR (95% CI)
**Age group**					
25-34	1.00	1.00	1.00	1.00	1.00
35-44	0.71 (0.30-1.68)	0.79 (0.44-1.44)	0.73 (0.43-1.23)	0.86 (0.50-1.50)	1.16 (0.73-1.83)
45-54	0.40 (0.17-0.92)*	0.60 (0.33-1.09)	0.70 (0.41-1.20)	0.49 (0.29-0.84)**	0.65 (0.41-1.05)
55-64	1.06 (0.31-3.66)	0.28 (0.15-0.55)***	0.36 (0.19-0.66)**	0.48 (0.26-0.87)*	0.93 (0.53-1.62)
**Social grade**					
AB	1.00	1.00	1.00	1.00	1.00
C1	1.00 (0.42-2.36)	1.16 (0.63-2.11)	0.75 (0.41-1.38)	1.25 (0.74-2.11)	1.15 (0.71-1.88)
C2	3.47 (1.05-11.47)*	0.97 (0.53-1.77)	0.57 (0.31-1.03)	2.37 (1.32-4.23)**	1.84 (1.10-3.06)*
D	2.11 (0.63-7.04)	1.71 (0.80-3.65)	0.81 (0.40-1.62)	1.85 (0.97-3.51)	1.83 (1.02-3.27)*
E	0.57 (0.17-1.87)	0.84 (0.36-1.93)	0.30 (0.14-0.65)**	2.69 (1.17-6.16)*	1.93 (0.94-3.97)
**Employment**					
Employed	1.00	1.00	1.00	1.00	1.00
Unemployed	0.90 (0.35-2.30)	1.34 (0.73-2.44)	1.05 (0.62-1.80)	1.27 (0.73-2.23)	1.46 (0.90-2.37)
Other (studying/retired)†	-	-	-	-	-
**Ethnicity**					
White	1.00	1.00	1.00	1.00	1.00
All other groups	0.39 (0.17-0.90)*	2.09 (0.86-5.08)	2.85 (1.24-6.57)*	1.34 (0.67-2.72)	1.31 (0.73-2.36)
**Caring responsibilities**					
No	1.00	1.00	1.00	1.00	1.00
Yes	1.50 (0.76-2.96)	0.95 (0.60-1.51)	1.10 (0.72-1.70)	1.52 (1.00-2.32)	1.14 (0.78-1.67)

Note. Reference group: 'unacceptable/ambivalent'. OR= adjusted odds ratio; CI= confidence interval; *p<0.05, **p<0.01, ***p<0.001; 'screening status' and 'practical barriers' variables not included because not significant in univariable analyses; †category not included due to insufficient cases

Table S4

Multivariable logistic regression models of predictors of phone-based booking preferences

	**Calling the GP (n=596)**	**Calling a 24-hour automated service (n=590)**	**Requesting a call-back (n=593)**
	OR (95% CI)	OR (95% CI)	OR (95% CI)
**Age group**			
25-34	1.00	1.00	1.00
35-44	1.05 (0.47-2.36)	0.69 (0.45-1.06)	1.05 (0.68-1.63)
45-54	0.60 (0.27-1.35)	0.50 (0.31-0.79)**	0.65 (0.41-1.02)
55-64	0.96 (0.34-2.75)	0.46 (0.27-0.78)**	1.30 (0.76-2.22)
**Caring responsibilities**			
No	1.00	1.00	1.00
Yes	0.84 (0.43-1.67)	0.92 (0.64-1.33)	1.74 (1.20-2.52)**
**Screening status**			
Intender	1.00	1.00	1.00
Maintainer	1.31 (0.70-2.43)	0.94 (0.67-1.32)	0.63 (0.45-0.90)*
**Practical barriers**			
0 barriers	1.00	1.00	1.00
1 barrier	1.00 (0.43-2.33)	0.83 (0.54-1.26)	0.73 (0.48-1.13)
2 barriers	1.05 (0.42-2.60)	1.24 (0.79 -1.96)	1.21 (0.76-1.93)
3 or more barriers cited	0.35 (0.15-0.83)*	1.59 (0.91-2.78)	1.17 (0.67-2.07)

Note. Reference group: ‘not likely to use/ambivalent’ OR= adjusted odds ratio; CI= confidence interval; *p<0.05, **p<0.01, ***p<0.001; ‘social grade’, ‘employment’ and ‘ethnicity’ not included because not significant in univariable analyses

Table S5

Multivariable logistic regression models of predictors of online booking preferences

	**Booking on a website using a desktop/laptop (n=589)**	**Booking on a website using a smartphone [a] (n=513)**	**Downloading an app to your smartphone [a] (n=517)**
	OR (95% CI)	OR (95% CI)	OR (95% CI)
**Age group**			
25-34	1.00	1.00	1.00
35-44	0.60 (0.38-0.95)*	0.59 (0.34-0.92)*	0.52 (0.33-0.82)**
45-54	0.52 (0.32-0.85)**	0.36 (0.21-0.61)***	0.35 (0.21-0.58)***
55-64	0.32 (0.18-0.55)***	0.25 (0.13-0.47)***	0.23 (0.12-0.44)***
**Social grade**			
AB	1.00	1.00	1.00
C1	0.59 (0.35-0.99)*	0.67 (0.38-1.18)	0.91 (0.54-1.54)
C2	0.51 (0.30-0.87)*	0.47 (0.26-0.83)*	0.82 (0.47-1.41)
D	0.47 (0.26-0.86)*	0.39 (0.20-0.73)**	0.62 (0.34-1.14)
E	0.34 (0.17-0.68)**	0.41 (0.18-0.94)*	0.76 (0.34-1.69)
**Employment**			
Employed	1.00	1.00	1.00
Unemployed	0.85 (0.54-1.35)	0.84 (0.50-1.41)	0.79 (0.48-1.31)
Other (studying/retired)	0.91 (0.42-1.97)	0.55 (0.22-1.35)	0.35 (0.13-0.91)*
**Practical barriers**			
0 barriers	1.00	1.00	1.00
1 barrier	1.35 (0.87-2.08)	1.18 (0.73-1.91)	1.21 (0.75-1.93)
2 barriers	1.67 (1.04-2.69)*	1.52 (0.90-2.54)	1.66 (1.00-2.73)*
3 or more barriers	2.07 (1.15-3.73)*	2.74 (1.41-5.33)**	2.46 (1.00-2.73)**

Note. Reference group: 'not likely to use/ambivalent' OR= adjusted odds ratio; CI= confidence interval; *p<0.05, **p<0.01, ***p<0.001; [a] participants with no smartphone removed from analyses (n = 81); 'ethnicity', 'caring responsibilities' and 'screening status' not included because not significant in univariable analyses
