# Supplementary material for: Could changing invitation and booking processes help women translate their cervical screening intentions into action? A population-based survey of women’s preferences in Great Britain
Source: BMJ Open. 2019 Jul 11;9(7):e028134. doi: 10.1136/bmjopen-2018-028134 (PMC6629419; doi:10.1136/bmjopen-2018-028134)
Supplement: Supplementary file 3 [file bmjopen-2018-028134supp003.pdf]

Online Supplement 3: Could changing invitation and booking processes help women translate their cervical screening intentions into action? A population-based survey of women's preferences in Great Britain.

(Mairead Ryan, Jo Waller and Laura Marlow)

Survey inclusion flow diagram

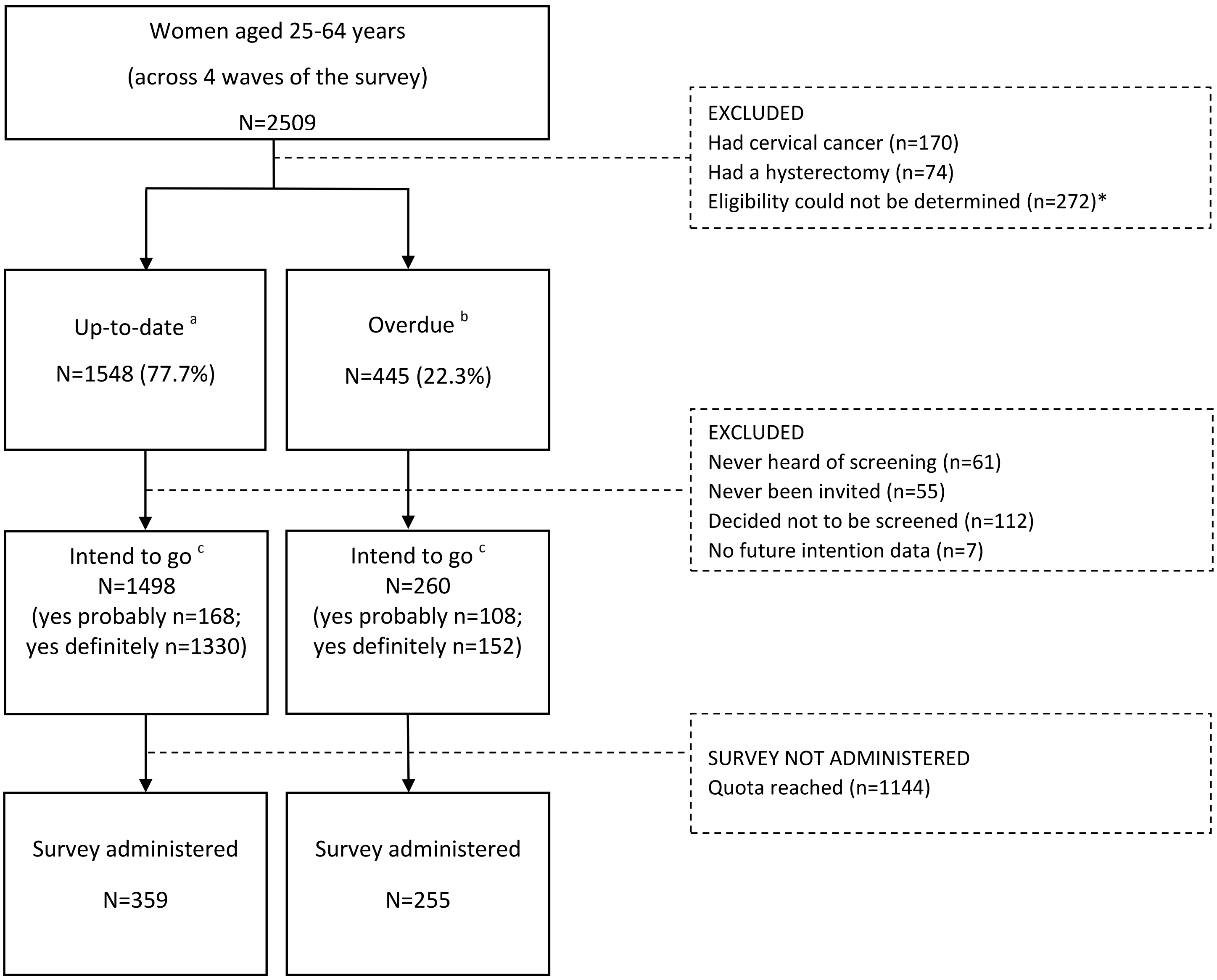

*Women who refused to answer the hysterectomy question (n=177) or screening uptake question (n=95)

[a] Up-to-date: been screened within the last 3 years if 25-64 years or the last 5 years if 50-64 years

[b] Overdue: not been screened within the last 3 years if 25-64 years or the last 5 years if 50-64 years

[c] Responded that they would 'probably' or 'definitely' attend screening when next invited
