# Supplementary material for: Could changing invitation and booking processes help women translate their cervical screening intentions into action? A population-based survey of women’s preferences in Great Britain
Source: BMJ Open. 2019 Jul 11;9(7):e028134. doi: 10.1136/bmjopen-2018-028134 (PMC6629419; doi:10.1136/bmjopen-2018-028134)
Supplement: Supplementary file 4 [file bmjopen-2018-028134supp004.pdf]

Online Supplement 4: Could changing invitation and booking processes help women translate their cervical screening intentions into action? A population-based survey of women's preferences in Great Britain.

(Mairead Ryan, Jo Waller and Laura Marlow)

Open responses provided for citing invitation method as unacceptable

**Invitation mode**	**Unacceptable (n)**	**Reasons for being unacceptable**
Posted letter	12	Don't open post/might miss the letter/no time to read letter (n=4)<br>Receive letter too late (n=2)<br>Letter could be lost in the post (n=2)<br>Other (n=4)<br>• Would forget (n=1)<br>• Environmental concerns (n=1)<br>• Waste of time (n=1)<br>• No reason provided (n=1)
Text-message	67	Privacy concerns (n=21)<br>Easy to miss it/may not read message (n=9)<br>Reason not provided (i.e. N/A) (n=9)<br>Doesn't have or use mobile (n=7)<br>Impersonal (n=6)<br>Could change number (n=4)<br>Prefer a letter/phone call (n=4)<br>Not reliable source/unprofessional (n=3)<br>Would forget/not act on it (n=2)<br>Other (n=2)<br>• Don't know (n=1)<br>• They can text me but I don't want to text them (n=1)
Email	94	Would be lost in other emails/would not be seen (n=38)<br>No email/doesn't use email/no internet/no computer (n=17)<br>Privacy concerns (n=12)<br>Reason not provided (i.e. N/A) (n=12)<br>Prefer phone or letter (n=5)<br>Would forget/not act on it (n=2)<br>Impersonal/rude (n=2)<br>Other (n=6)<br>• Not timely (n=1)<br>• Intrusive (n=1)<br>• Not normal (n=1)<br>• No reason (n=1)<br>• Not keen (n=1)<br>• Doesn't trust source (n=1)
Mobile	90	Would not be able to pick up/would miss call (n=33)

phone call		Privacy concerns (n=22)<br>Would prefer in writing/a letter (n=10)<br>Reason not provided (i.e. N/A) (n=8)<br>Would not know number – so would not answer call (n=5)<br>No mobile (n=2)<br>Would forget (n=2)<br>Too many phone calls (n=2)<br>Other (n=6)<br>• Don't like idea (n=1)<br>• Talking takes too much time (n=1)<br>• Need time to think (n=1)<br>• Impersonal (n=1)<br>• People change phone number (n=1)<br>• Don't like calls (n=1)
Landline phone call	129	No landline (n=39)<br>Would miss call/out of the house during the day (n=31)<br>Privacy concerns (n=24)<br>No reason provided (i.e. N/A) (n=12)<br>Feels intrusive (n=5)<br>Prefer in writing/letter (n=5)<br>Don't want phone call (n=4)<br>Not reliable source (n=3)<br>Other (n=6)<br>• Impersonal (n=1)<br>• "Better with working" (n=1)<br>• Unnecessary (n=1)<br>• Unknown number (n=1)<br>• Want time to think (n=1)<br>• Doesn't matter either way (n=1)
