# Supplementary material for: Could changing invitation and booking processes help women translate their cervical screening intentions into action? A population-based survey of women’s preferences in Great Britain
Source: BMJ Open. 2019 Jul 11;9(7):e028134. doi: 10.1136/bmjopen-2018-028134 (PMC6629419; doi:10.1136/bmjopen-2018-028134)
Supplement: Author's manuscript [file bmjopen-2018-028134.draft_revisions.pdf]

BMJ Open is committed to open peer review. As part of this commitment we make the peer review history of every article we publish publicly available.

When an article is published we post the peer reviewers' comments and the authors' responses online. We also post the versions of the paper that were used during peer review. These are the versions that the peer review comments apply to.

The versions of the paper that follow are the versions that were submitted during the peer review process. They are not the versions of record or the final published versions. They should not be cited or distributed as the published version of this manuscript.

BMJ Open is an open access journal and the full, final, typeset and author-corrected version of record of the manuscript is available on our site with no access controls, subscription charges or pay-per-view fees (http://bmjopen.bmj.com).

If you have any questions on BMJ Open's open peer review process please email info.bmjopen@bmj.com

BMJ Open

Could changing invitation and booking processes help women translate their cervical screening intentions into action: a population-based survey of women's preferences

Journal:	*BMJ Open*
Manuscript ID	bmjopen-2018-028134
Article Type:	Research
Date Submitted by the Author:	23-Nov-2018

Complete List of Authors:	Ryan, Mairead; University College London Institute of Epidemiology and Health Care, Department of Behavioural Science and Health;<br>Waller, Jo; UCL, Epidemiology and Public Health<br>Marlow, Laura; University College London

Keywords:	cervical cancer screening, non-participants, interventions, age, screening status, uptake

SCHOLARONE™
Manuscripts

Could changing invitation and booking processes help women translate their cervical screening intentions into action: a population-based survey of women's preferences

Mairead Ryan, MSc

Jo Waller, PhD

Laura A.V Marlow, PhD

Cancer Communication and Screening Group, Research Department of Behavioural Science and Health, University College London, Gower Street, London, UK

Corresponding author contact details: Laura Marlow, l.marlow@ucl.ac.uk, 020 7679 1798.

MR, JW and LM are supported by a Cancer Research UK Career Development Fellowship awarded to JW (C7492/A17219).

For peer review only - http://bmjopen.bmj.com/site/about/guidelines.xhtml

Abstract

Objectives: Many women who do not attend screening intend to go, but do not get around to booking an appointment. Qualitative work suggests these 'intenders' face more practical barriers to screening than women who are up-to-date ('maintainers'). This study explored practical barriers to booking a screening appointment and preferences for alternative invitation and booking methods that might overcome these barriers.

Design: A cross-sectional survey was employed.

Setting: Great Britain

Participants: Women aged 25 to 64, living in Great Britain who intended to be screened but were overdue ('intenders', n=255) and women who were up-to-date with screening ('maintainers', n=359)

Results: 'Intenders' reported slightly more barriers than 'maintainers' overall (mean = 1.36 vs 1.06, t=3.03, p <0.01) and were more likely to think they might forget to book an appointment (Odds ratio=2.87, 95% confidence interval: 2.01-4.09). Over half of women said they would book on a website using a smartphone (62%) or a computer (58%). Older women and women from lower social grades were less likely to say they would use online booking methods (all ps<.05). Women who reported two or more barriers were more likely to say they would use online booking than women who reported none (ps<.01).

Conclusions: Women who are overdue for screening face practical barriers to booking appointments. Tailoring the appointment booking process to the preferences cited in this study may help women overcome logistical barriers to participation and increase coverage for cervical screening.

Strengths and limitations of this study

- Booking preferences were assessed in women who are up-to-date and overdue for cervical screening

For peer review only - http://bmjopen.bmj.com/site/about/guidelines.xhtml

- The invitation and booking process was broken down into its component parts to identify barriers at each stage of the process as well as options which may help women to overcome these barriers

- The practical barriers explored within this study were not exhaustive of all barriers faced by women

Introduction

Cervical screening programmes are designed to reduce the incidence and mortality rate of cervical cancer.[1] In Great Britain all eligible women aged 25 to 64 registered with a GP are invited to be screened for the presence of abnormal cell changes in the cervix, which could, if undetected and untreated, develop into cervical cancer. The efficacy of the programme has been widely acknowledged,[2] however the success of any screening programme is dependent on good coverage. In 2017, coverage (i.e. the percentage of eligible women recorded as adequately screened) was 72%, well below the national target of 80% and in keeping with a trend of decreasing screening coverage.

Reasons for screening non-attendance are complex and differ depending on socio-demographic factors such as age, socio-economic status and marital status.[3-6] Emotional barriers including embarrassment, fear of pain and negative experiences are often reported, particularly in qualitative studies.[7-9] While these barriers undoubtedly need to be addressed, practical barriers have been found to be more predictive of screening status than emotional barriers.[10] Recent research showed that over half of women overdue for cervical screening have positive intentions to attend.[11] While this is encouraging, intentions are frequently not translated into action.[12, 13]

Weinstein used a 'messy desk' analogy to help explain the problem of translating intentions into action.[14] He proposed that we do not carry out errands in a logical sequence, but rather in a haphazard manner, acting on 'to-do' list items when we feel pressure, when items need to be actioned quickly, when prompted or because of personal preference. More recently, Sheeran and Webb identified three key problems (or 'TRIALS') people might encounter when trying to realise their intentions; i) they fail to get started (e.g. forget to act or miss an opportunity to act), ii) they fail to keep the goal on track (fail to monitor the goal, face competing thoughts or distractions) and iii) they fail to close (don't quite meet the goal).[15]

Women receive a posted letter inviting them to book a screening appointment. The letter states the recipient "can make an appointment for cervical screening by phoning *(their)* GP surgery". GP surgery hours generally coincide with 'normal' working hours, presenting several practical barriers

For peer review only - http://bmjopen.bmj.com/site/about/guidelines.xhtml

for women who are in full-time employment or who have caring responsibilities, both in terms of phoning and attending a GP surgery. Previous research has identified that many women find the booking process arduous and inflexible.[3]

Few studies have assessed alternative methods of inviting women for cervical screening.[16] The most recent Cochrane review of interventions to improve uptake [16] reported two studies from the 1980s and 90s, which found that participants who received a telephone invitation were significantly more likely to attend than those who received a letter.[17, 18] Studies which have examined the utility of more recent technological developments to invite women are lacking.[19] There is also a paucity of literature concerning alternative booking methods for cervical screening, most likely due to limited booking options being available until recently. One trial investigated the efficacy of online booking among first time invitees.[20] The intervention group booked slightly more appointments within three months (2.18% higher than the control group) however, this was not statistically significant.[20] The authors noted that the way the online booking system was offered could account for the lack of support (in a letter participants were asked to visit a website to book at one of three sexual health clinics). Hence, other forms of online booking may be desirable to women.

New technologies offer opportunities for editing the architecture of the invitation and booking system in ways that may help to overcome some of the challenges women face between forming a positive intention and translating this into behaviour. The present study explored practical barriers to booking an appointment among two groups: women who are up-to-date with screening ('maintainers') and women who intend to be screened but are currently overdue ('intenders'), the aim of which was to examine any between-group differences which may account for this intention-behaviour gap among 'intenders'. We also assessed invitation and booking preferences and explored whether these might help to overcome practical barriers.

Methods

Participants

Participants were recruited by Kantar TNS UK as part of their omnibus survey. The TNS omnibus survey recruits a new sample of 2000-4000 men and women living in Great Britain on a weekly basis and asks questions on a range of topics commissioned by external companies. Recruitment uses random location sampling to identify areas for sampling participants using the 2011 Census and the Postcode Address File. Recruiters visit homes in the identified areas and knock on doors asking those who answer to participate. All interviews were conducted in English. Quotas are set at each location for age, gender, working status, and presence of children in the household.

For peer review only - http://bmjopen.bmj.com/site/about/guidelines.xhtml

Women who were eligible for cervical screening and had not previously been diagnosed with cervical cancer, were asked to report their past attendance at cervical screening and future intention to attend (see Online Supplement 1). Responses to these questions were used to classify women as ‘intenders’ (intended to be screened but were currently overdue), ‘maintainers’ (up-to-date with screening and intending to go in the future) or ‘other’ (never heard of screening, never been invited, decided not to be screened). A sample of 600 women was expected to allow us to establish a significant difference of 5% between preferred booking options in the two groups of attenders within +/- 8% with 95% confidence.

Procedure

Ethical approval was granted by University College London Research Ethics Committee (reference: 10353/003). All questions were piloted with women eligible for screening (*n=10*). Data were collected between April and May 2018. Face-to-face computer-assisted personal interviews were used to collect data. Kantar TNS provided anonymised data to UCL for analysis.

Measures

Invitation preferences: Participants were asked whether several different modes of communication were acceptable to them as a means of being invited to book a cervical screening appointment (see Online Supplement 1). Participants were considered to find a mode of communication ‘acceptable’ if they responded quite/very acceptable or ‘unacceptable’ if they responded quite/very unacceptable, neither unacceptable or acceptable, don’t know or not applicable. Participants who responded quite/very unacceptable were asked to explain why (open response).

Practical barriers to booking an appointment: Participants were asked to respond to a list of barriers, which were based on the key problems outlined in the TRIALS model.[15] Statements addressing the key problem of ‘failing to get started’ included ‘It is easy for me to find time to read a letter like this’ and ‘I might forget to book an appointment after reading this letter’. Statements addressing ‘failing to keep the goal on track’ included ‘It is difficult for me to call my GP practice during their opening hours’ and ‘I find it difficult to get through to a receptionist when I phone my GP practice’. Women were then asked to state which booking attributes were important to them, the aim of which was to address factors that might influence ‘failure to close’ (i.e. being able to book the appointment).

Booking preferences: Participants were asked to indicate how likely they would be to use different booking methods. Participants were considered ‘likely to use’ a method if they responded quite/very likely or ‘not likely to use’ if they responded quite/very unlikely, neither unlikely nor likely, don’t know or not applicable. Participants were also asked to indicate which booking methods they had used in the past for any GP appointment.

For peer review only - http://bmjopen.bmj.com/site/about/guidelines.xhtml

Socio-demographic and background factors: Data regarding age, ethnicity, education level, employment status, marital status, social grade, child/carer responsibilities and smartphone ownership were also collected. Social grade is determined by the occupation of the Chief Income Earner in the household and is classified as follows: AB managerial/professional; C1 supervisory; C2 skilled manual; D semi-skilled/unskilled manual; E casual workers/unemployed.[21]

Patient and Public Involvement Statement

The study was supported by a PPI group who provided input into the contents of the survey. A group of 10 screening-eligible women were invited to guide and refine the survey questions. Women who were both up-to-date and overview were represented in the group. The group helped to establish the perceived difficulty of the questions (e.g. unknown terms, ambiguous concepts, long and overly complex questions) and omissions from the survey. The questions and response options were tailored based on feedback provided by this PPI group.

Analyses

All analyses were conducted using IBM SPSS version 22. Chi-squared analyses were conducted to test for significant differences in participant demographics between 'Intenders' and 'Maintainers'. Descriptive statistics were conducted to assess booking history and smartphone/mobile phone ownership across all participants. For each of the six practical barrier statements, any positively-framed items were reverse-scored so that a higher score was indicative of a barrier for all items. Total practical barrier scores were created by allocating a score of 1 for each barrier statement that a participant 'agreed' or 'strongly agreed' with and adding these together (possible range 0-6). Independent samples t-tests were conducted to assess differences in the mean barriers scores between 'intenders' and 'maintainers'. A series of binary logistic regressions were then conducted to assess the associations between endorsing each barrier/booking attribute and the unadjusted odds for being an 'intender' (versus a 'maintainer'). A series of univariable logistic regressions were conducted to explore whether socio-demographic factors, screening status and number of practical barriers reported were associated with invitation and booking preferences. Multivariable logistic regressions are presented as supplementary material.

Results

Sample characteristics

Over four weeks of the survey, 2088 respondents were identified as being eligible for cervical screening and had not previously had cervical cancer. Of these, 1548 (74%) were up-to-date and 445 (21%) were overdue for screening. Our questions on invitation and booking preferences for cervical

For peer review only - http://bmjopen.bmj.com/site/about/guidelines.xhtml

screening were asked to all women who were classified as 'intenders' (n=255) and women who were classified as 'maintainers' (n=359) in the first week.

Sample characteristics for participants classified as 'intenders' (n=255) and 'maintainers' (n=359) are presented in Table 1. Mean age was 41.69 years (SD=10.84, range: 25-64 years), the majority self-identified as White (89%), were employed (64%), married or co-habiting (67%) and had regular caring responsibilities (i.e. for children /parents; 63%). 'Intenders' (mean=39.41; SD=9.94) were significantly younger than 'maintainers' (mean=43.31; SD=11.16); $t(612)=4.47$, $p<.001$.

The majority of women had previously booked by phoning the practice (89%), over one-third had booked in person (39%) and 14% had booked on a website. 'Maintainers' were significantly more likely to have previously booked on a website than 'intenders' (see Table 1). The majority of participants had a smartphone (87%), fewer women had a mobile phone which was not a smartphone (11%) and a small minority had no mobile phone (2%).

Practical barriers to appointment booking and desired attributes

Over two-thirds of women reported one or more barriers to booking (69%); mean number of reported barriers was 1.21 (SD=1.06). 'Intenders' (mean=1.36; SD=1.06) reported slightly more barriers than 'maintainers' overall (mean=1.10; SD=1.04; $t(612)=3.03$, $p<0.01$). The most commonly endorsed barriers and desired booking attributes are outlined in Table 2. The 'intenders' group were significantly more likely to endorse the statement 'I might forget to book an appointment after reading this letter' than 'maintainers'. 'Intenders' were also more likely to state 'How long it takes to book the appointment' was important to them than 'maintainers'.

Invitation preferences

Posted letters emerged as the most acceptable invitation mode followed by text-message (see Table 3). Socio-demographic predictors of the acceptability of each modality are shown in Table 3. Text-message, email and mobile call invitations were less acceptable to women aged 55-64; these associations remained significant in multivariable analyses (see Online Supplement 2). Mobile and landline call invites were more acceptable to women from lower socio-economic backgrounds and this remained significant in multivariable analyses for mobile invites. Reasons for considering invitation modes as unacceptable are provided in Online Supplement 3; fears about missing a phone call/email or text and privacy concerns were commonly cited. Many participants also reported they had no landline phone.

Phone-based booking preferences

For peer review only - http://bmjopen.bmj.com/site/about/guidelines.xhtml

Most women said they were likely to book by phoning their GP practice (90%; see Table 4). Older women were significantly less likely to say they would call a 24-hour automated service than women aged 25-34 (41% vs 61%). Women with caring responsibilities were more likely to say they would request a call-back compared to women with no caring responsibilities (62% vs 49%). 'Maintainers' were less likely to say they would request a call-back than 'intenders' (63% vs 53%). These associations remained significant in multivariable analyses. Women who cited three or more barriers were more likely to say they would call a 24-hour automated service but this association was not significant in multivariable analyses.

Online booking preferences

Booking on a website using a smartphone (62%) was the preferred online booking method (see Table 5). Older women (55-64 years) were less likely to say they would book online than younger women (25-34 years). Women in lower social grades were less likely than women in the highest grade to state they would book on a website, either using a desktop or smartphone. Participants who were studying or retired were less likely than those employed to say they would book online (either on a website using a smartphone: 41% vs 65%, or through an app: 24% vs 56%). Women who reported two or more barriers were more likely to report that they would use all online booking methods compared to women who reported no barriers (see Table 5). Age, social grade and number of barriers remained significant in multivariable analyses.

Discussion

This study examined women's practical barriers to booking a cervical screening appointment and assessed whether invitation and booking preferences are associated with reported barriers, socio-demographic factors and screening status. Approximately one-third of all women reported that it is difficult to phone their GP practice within opening hours and half reported that it is difficult to get through to a receptionist. Although the survey found that 'intenders' experience slightly more practical barriers to screening than 'maintainers', endorsement of barriers across the sample suggests that both groups need more support in booking an appointment.

'Intenders' were more likely to report that they would forget to book an appointment after reading the screening letter than 'maintainers'. This key problem relates to a 'failure to get started', which is a first barrier people face between forming an intention and translating this into behaviour.[15] Written reminders are an integral part of the screening programme and there is good evidence to show these improve uptake,[16] but in their current format these reminders do not seem to help all

For peer review only - http://bmjopen.bmj.com/site/about/guidelines.xhtml

women to remember to book their appointment. The use of text-message reminders has shown promise in other screening contexts.[22] ‘Intenders’ were also more likely to say that the length of time needed to book an appointment was important to them. Since all women eligible for cervical screening fall within the working age population, and GP opening hours generally overlap with working hours, it is likely this cohort face competing obligations,[23] and, as a result ‘fail to keep their goal on track’.[15] The rate of female employment (16 to 64 years) has increased from 62.2% in 1994, when coverage was high (85%; five yearly coverage for women aged 20 to 64)[24] to 70.5% in 2017.[25] Alternative booking methods may provide more flexibility.

Women who reported more barriers showed greater interest in using alternative booking methods. Specifically, participants who reported two or more barriers were more likely to say that they would book on a website or through an app. This is perhaps not surprising since these methods overcome the most common practical barriers highlighted by participants, including, difficulty getting through to a receptionist and difficulty calling the practice during opening hours. While online booking services are already set up in the majority of GP practices across England for GP appointments, a national survey found that over 40% of patients are currently unaware if there are online booking services at their GP practice.[26] Hence, signposting online booking services, if available for nurse appointments, to groups of the screening-eligible population (i.e. younger women who are more likely to be ‘intenders’) may be an effective means of increasing uptake. This survey suggests that there are likely to be age and socio-economic inequalities in the use of online bookings. For example, women aged 45-54 years and women age 55-64 showed less interest in using online booking methods. Thus, ensuring that traditional telephone booking options remains available is important.

Previous research has found that it is very difficult for individuals to maintain intentions after even very brief periods of time (less than one minute), especially in circumstances where there are competing tasks.[27] Unlike posted letters, which may not be read until the end of the day, text-messages can be delivered at a time when GP practices are open, so women can act immediately on their intentions to book an appointment. Given that text-message invites were considered acceptable to the majority of women across all socio-demographic backgrounds, and have previously been found to be effective in increasing uptake for other national screening programmes,[22] the use of text-message invitations may be a worthwhile intervention to explore. Text-messages within the cervical screening programme have, thus far, been introduced as a booking reminder, rather than as a stand-alone invitation, which the current study did not specify.

For peer review only - http://bmjopen.bmj.com/site/about/guidelines.xhtml

Some participants shared concerns that they may miss the message; outlining that text-messages would be used as a supplemental invitation may have further increased acceptability within the sample. Further research is needed to explore methods of overcoming privacy concerns associated with text-messages, which some of the participants raised.

This study had some limitations. We were unable to collect data on women who elected not to participate in the study. Hence the response rate and differences between respondents and non-respondents could not be determined. This survey was also conducted in English and therefore non-English speakers were not represented. Given ethnic disparities in screening attendance in England,[28] more work is needed to explore methods of overcoming practical barriers to screening for ethnic minority women. Furthermore, although this study explored practical barriers to appointment-booking based on the TRIALS model,[15] several other practical barriers were not assessed. For example, previous research has found that ‘intenders’ are more likely to have children under the age of five;[11] childcare may be an additional practical barrier to screening. Thus the barriers cited in this study are not exhaustive of all practical barriers to screening for women.

Nevertheless, this was the first study to assess preferences for booking a screening appointment in Great Britain. The invitation and booking process was broken down to identify barriers at each stage and associated preferences which may help women to overcome such barriers. The lack of differences by screening status negates any concerns that changing the architecture might deter ‘maintainers’ from participation. Future interventions may assess the efficacy of i) signposting invitees to online booking services, ii) text-messages which are delivered during GP opening hours and iii) sending reminders to reduce the likelihood of forgetting to book an appointment.

For peer review only

For peer review only - http://bmjopen.bmj.com/site/about/guidelines.xhtml

Table 1:

Sample Characteristics (n=614)

	Overall (n=614)	Maintainers (n=359)	Intenders (n=255)	Difference between maintainers and intenders
	N (%)	N (%)	N (%)	Chi Square (df), P-value
**Age (years)**				14.16 (3), <.001
25-34	192 (31.3)	103 (28.7)	89 (34.9)	
35-44	183 (29.8)	95 (26.5)	88 (34.5)	
45-54	137 (22.3)	88 (24.5)	49 (19.2)	
55-64	102 (16.6)	73 (20.3)	29 (11.4)	
**Ethnicity**				0.10 (1), 0.76
White	547 (89.1)	321 (89.4)	226 (88.6)	
All other groups	67 (10.9)	38 (10.6)	29 (11.4)	
**Education level**				2.12 (4), 0.71
GCSE or below	180 (29.3)	108 (30.1)	72 (28.2)	
A level or equivalent	71 (11.6)	45 (12.5)	26 (10.2)	
College qualification	115 (18.7)	62 (17.3)	53 (20.8)	
Degree or higher	213 (34.7)	125 (34.8)	88 (34.5)	
Other	35 (5.7)	19 (5.3)	16 (6.3)	
**Employment status**				3.19 (2), 0.20
Employed (full-time/part-time)	392 (63.8)	234 (65.2)	158 (62.0)	
Unemployed	182 (29.6)	98 (27.3)	84 (32.9)	
Other	40 (6.5)	27 (7.5)	13 (5.1)	
**Marital status**				2.89 (2), 0.24
Single	129 (21.0)	67 (18.7)	62 (24.3)	
Married/living as married	413 (67.3)	249 (69.4)	164 (64.3)	
Widowed/divorced/separated	72 (11.7)	43 (12.0)	29 (11.4)	
**Parent/carer role**				0.62 (0.45), 0.43
Yes	387 (63.0)	221 (61.6)	166 (65.1)	
No	222 (36.2)	134 (37.3)	88 (34.5)	
**Social status**				7.93 (4), 0.09
AB (highest)	134 (21.8)	90 (25.1)	44 (17.3)	
C1	157 (25.6)	88 (24.5)	69 (27.1)	
C2	142 (23.1)	84 (23.4)	58 (22.7)	
D	93 (15.1)	54 (15.0)	39 (15.3)	
E (lowest)	88 (14.3)	43 (12.0)	45 (17.6)	
**Booking history (Yes/No)**				
Phoned the practice	545 (88.8)	316 (88.0)	229 (89.8)	0.47 (1), 0.49
At reception (in person)	240 (39.1)	145 (40.4)	95 (37.3)	0.62 (1), 0.43
24-hr automated service	23 (3.7)	14 (3.9)	9 (3.5)	0.06 (1), 0.81
Text-message	7 (1.1)	4 (1.1)	3 (1.2)	0.01 (1), 0.94
Website	85 (13.8)	60 (16.7)	25 (9.8)	5.97 (1), <.05
Smartphone app	23 (3.7)	15 (4.2)	8 (3.1)	0.45 (1), 0.50
**Phone ownership**				0.72 (2), 0.70
Smartphone	533 (86.8)	315 (87.7)	218 (85.5)	
Non-smartphone mobile	67 (10.9)	36 (10.0)	31 (12.2)	
No phone	14 (2.3)	8 (2.2)	6 (2.4)	

For peer review only - http://bmjopen.bmj.com/site/about/guidelines.xhtml

Table 2:

Practical barriers to appointment booking and booking characteristics considered to be important (n=614)

	All (*n=614*) N (%)	'Maintainers' (*n=359*) N (%)	'Intenders' (*n=255*) N (%)	OR for being an 'intender' (95% CI)
**Practical barriers to booking screening (% agree/strongly agree)**				
It is (*not*) easy for me to find time to read a letter like this	25 (4.1)	15 (4.2)	10 (3.9)	0.94 (0.41-2.12)
I might forget to book an appointment after reading this letter	187 (30.5)	76 (21.2)	111 (43.5)	2.87 (2.01-4.09)**
It is difficult for me to call my GP practice during their opening hours	192 (31.3)	108 (30.1)	84 (32.9)	1.14 (0.81-1.61)
I (*do not*) have access to a telephone/mobile with phone credit/minutes to call my GP practice	13 (2.1)	8 (2.2)	5 (2.0)	0.88 (0.28-2.71)
I would (*not*) find it easy to find the phone number for my GP practice to contact them	19 (3.1)	11 (3.1)	8 (3.1)	1.01 (0.41-2.59)
I find it difficult to get through to a receptionist when I phone my GP practice	306 (49.8)	177 (49.3)	129 (50.6)	1.05 (0.76-1.45)
**Booking attributes (% saying quite/very important)**				
Ease of booking	519 (84.5)	305 (85.0)	214 (83.9)	0.92 (0.59-1.44)
Choice of appointments	486 (79.2)	280 (78.0)	206 (80.8)	1.19 (0.83-1.77)
Being able to change an appointment after booking	474 (77.2)	274 (76.3)	200 (78.4)	1.13 (0.77-1.66)
How long it takes to book appointment	424 (69.1)	235 (65.5)	189 (74.1)	1.51 (1.06-2.15)*
Waiting time for next available appointment	428 (69.7)	245 (68.2)	183 (71.8)	1.18 (0.83-1.68)
Privacy when booking appointment	410 (66.8)	230 (64.1)	180 (70.6)	1.35 (0.95-1.90)
Being able to talk with a healthcare professional when booking	345 (56.2)	195 (54.3)	150 (58.8)	1.20 (0.87-1.66)
Being able to book an appointment when the GP practice is shut	284 (46.3)	173 (48.2)	111 (43.5)	0.83 (0.60-1.15)[a]
Cost of making booking (i.e. phone credit)	166 (27.0)	94 (26.2)	72 (28.2)	1.11 (0.77-1.59)

Note. OR= odds ratio; CI= confidence interval; *p<0.05, **p<0.001, [a]30% missing data for this variable

For peer review only

For peer review only - http://bmjopen.bmj.com/site/about/guidelines.xhtml

Table 3:

Univariable logistic regression models of predictors of the acceptability of cervical screening invitation modalities (n=614)

	Posted letter		Text-message		Email		Mobile phone call		Landline phone call	
	%	OR (95% CI)	%	OR (95% CI)	%	OR (95% CI)	%	OR (95% CI)	%	OR (95% CI)
**All participants**	90.1	1.00	78.5	1.00	72.5	1.00	73.8	1.00	58.5	1.00
**Age group**										
25-34	92.2	1.00	86.7	1.00	80.9	1.00	82.4	1.00	65.0	1.00
35-44	89.1	0.69 (0.34-1.39)	84.2	0.78 (0.45-1.34)	78.2	0.76 (0.47-1.23)	80.8	0.85 (0.52-1.41)	67.5	1.06 (0.70-1.61)
45-54	86.9	0.56 (0.27-1.16)	78.7	0.64 (0.36-1.12)	74.1	0.71 (0.43-1.19)	69.1	0.52 (0.31-0.87)*	53.4	0.67 (0.43-1.04)
55-64	92.2	1.00 (0.41-2.43)	65.6	0.29 (0.16-0.50)***	60.0	0.33 (0.20-0.56)***	62.9	0.36 (0.21-0.61)***	60.4	0.85 (0.52-1.38)
**Social grade**										
AB	89.6	1.00	76.1	1.00	77.6	1.00	62.7	1.00	49.3	1.00
C1	85.4	0.68 (0.34-1.38)	77.1	1.05 (0.61-1.82)	73.2	0.79 (0.46-1.35)	67.5	1.24 (0.76-2.01)	51.6	1.10 (0.69-1.74)
C2	96.5	3.20 (1.12-9.14)*	78.9	1.17 (0.67-2.06)	72.5	0.76 (0.44-1.32)	82.4	2.79 (1.60-4.86)***	65.5	1.96 (1.21-3.17)**
D	93.5	1.69 (0.63-4.58)	84.9	1.77 (0.89-3.54)	77.4	0.99 (0.53-1.86)	77.4	2.04 (1.12-3.72)*	63.4	1.79 (1.04-3.07)*
E	85.2	0.67 (0.30-1.51)	77.3	1.07 (0.56-2.02)	58.0	0.40 (0.22-0.72)**	84.1	3.15 (1.61-6.15)**	68.2	2.01 (1.26-3.87)**
**Employment**										
Employed	91.1	1.00	78.8	1.00	76.0	1.00	72.4	1.00	55.1	1.00
Unemployed	86.8	0.65 (0.37-1.12)	79.7	1.05 (0.68-1.63)	67.0	0.64 (0.44-0.94)*	80.8	1.60 (1.04-2.46)*	66.5	1.62 (1.12-2.33)*
Other (studying/retired)	95.0	1.86 (0.43-8.05)	70.0	0.63 (0.61-1.29)	62.5	0.53 (0.27-1.04)	55.0	0.47 (0.24-0.90)*	55.0	0.99 (0.52-1.92)
**Ethnicity**										
White	91.0	1.00	77.7	1.00	71.1	1.00	73.1	1.00	57.6	1.00
All other groups	82.1	0.45 (0.23-0.90)*	85.1	1.64 (0.81-3.30)	83.6	2.07 (1.06-4.05)*	79.1	1.39 (0.75-2.58)	65.7	1.41 (0.83-2.40)
**Caring responsibilities**										
No	89.2	1.00	75.2	1.00	68.9	1.00	66.2	1.00	55.0	1.00
Yes	91.5	1.30 (0.75-2.26)	81.1	1.42 (0.95-2.11)	75.2	1.37 (0.95-1.97)	78.8	1.90 (1.31-2.75)**	61.0	1.28 (0.92-1.79)
**Screening status**										
Intender	88.6	1.00	79.6	1.00	72.2	1.00	74.1	1.00	58.0	1.00
Maintainer	91.1	1.31 (0.77-2.23)	77.7	0.89 (0.60-1.32)	72.7	1.03 (0.72-1.47)	73.5	0.97 (0.67-1.40)	58.8	1.03 (0.74-1.43)
**Practical barriers**										
0 barriers	89.6	1.00	77.2	1.00	68.9	1.00	73.6	1.00	56.0	1.00
1 barrier	93.2	1.57 (0.76-3.26)	77.9	1.04 (0.64-1.68)	72.1	1.17 (0.75-1.81)	74.7	1.06 (0.67-1.68)	57.9	1.08 (0.72-1.62)
2 barriers	90.7	1.12 (0.55-2.31)	80.0	1.18 (0.70-1.99)	74.7	1.33 (0.83-2.14)	72.7	0.96 (0.59-1.55)	60.7	1.21 (0.79-1.87)
3 or more barriers	82.7	0.55 (0.26-1.16)	80.2	1.20 (0.63-2.28)	77.8	1.58 (0.86-2.89)	74.1	1.03 (0.57-1.85)	61.7	1.27 (0.75-2.16)

Note. OR= unadjusted odds ratio; CI= confidence interval; *p<0.05, **p<0.01, ***p<0.05

For peer review only - http://bmjopen.bmj.com/site/about/guidelines.xhtml

Table 4

Univariable logistic regression models of predictors of phone-based booking preferences (n=614)

	Calling the GP		Calling a 24-hour automated service		Requesting a call-back	
	% likely to book by..	OR (95% CI)	% likely to book by..	OR (95% CI)	% likely to book by..	OR (95% CI)
**All participants**	89.6	1.00	51.6	1.00	57.0	1.00
**Age group**						
25-34	90.6	1.00	60.9	1.00	59.4	1.00
35-44	89.6	0.89 (0.45-1.76)	53.0	0.73 (0.48-1.09)	61.7	1.11 (0.73-1.67)
45-54	89.1	0.84 (0.41-1.73)	44.5	0.52 (0.33-0.80)**	48.2	0.64 (0.41-0.99)*
55-64	88.2	0.78 (0.36-1.68)	41.2	0.45 (0.28-0.73)**	55.9	0.87 (0.53-1.41)
**Social grade**						
AB	88.8	1.00	50.0	1.00	53.7	1.00
C1	86.0	0.77 (0.38-1.56)	49.0	0.96 (0.61-1.53)	49.0	0.83 (0.52-1.32)
C2	93.0	1.66 (0.72-3.85)	58.5	1.41 (0.87-2.26)	59.9	1.28 (0.80-2.07)
D	92.5	1.55 (0.61-3.96)	52.7	1.11 (0.66-1.89)	63.4	1.49 (0.87-2.57)
E	88.6	0.98 (0.42-2.30)	46.6	0.87 (0.51-1.50)	64.8	1.58 (0.91-2.76)
**Employment**						
Employed	89.8	1.00	52.8	1.00	55.9	1.00
Unemployed	89.6	0.98 (0.55-1.74)	50.0	0.89 (0.63-1.27)	61.5	1.26 (0.88-1.81)
Other (studying/retired)	87.5	0.80 (0.30-2.15)	47.5	0.81 (0.42-1.55)	47.5	0.72 (0.37-1.37)
**Ethnicity**						
White	89.8	1.00	50.3	1.00	56.3	1.00
All other groups	88.1	0.84 (0.38-1.85)	62.7	1.66 (0.97-2.80)	62.7	1.30 (0.77-2.20)
**Caring responsibilities**						
No	89.6	1.00	50.9	1.00	49.1	1.00
Yes	90.7	1.13 (0.65-1.96)	52.7	1.08 (0.77-1.50)	62.3	1.71 (1.23-2.39)**
**Screening status**						
Intender	87.8	1.00	53.7	1.00	63.1	1.00
Maintainer	90.8	1.37 (0.81-2.30)	50.1	0.87 (0.63-1.20)	52.6	0.65 (0.47-0.90)*
**Practical barriers**						
0 barriers	87.6	1.00	47.2	1.00	53.4	1.00
1 barrier	92.6	1.79 (0.89-3.57)	47.9	1.03 (0.69-1.54)	53.2	0.99 (0.66-1.48)
2 barriers	92.0	1.63 (0.79-3.37)	56.7	1.47 (0.95-2.25)	62.7	1.47 (0.95-2.27)
3 or more barriers cited	82.7	0.68 (0.33-1.39)	63.0	1.81 (1.06-3.07)*	61.7	1.57 (0.92-2.68)

Note. OR= unadjusted odds ratio; CI= confidence interval; *p<0.05, **p<0.01, ***p<0.001

For peer review only - http://bmjopen.bmj.com/site/about/guidelines.xhtml

Table 5

Univariable logistic regression models of predictors of online booking preferences (n=614)

	**Booking on a website using a desktop/laptop**		**Booking on a website using a smartphone [a]**		**Downloading an app to your smartphone [a]**	
	% likely to book by..	OR (95% CI)	% likely to book by..	OR (95% CI)	% likely to book by..	OR (95% CI)
**All participants**	57.8	1.00	56.4	1.00	47.4	1.00
**Age group**						
25-34	67.7	1.00	71.9	1.00	64.9	1.00
35-44	59.6	0.70 (0.46-1.07)	64.6	0.72 (0.45-1.12)	53.7	0.63 (0.41-0.97)*
45-54	54.0	0.56 (0.36-0.88)*	53.1	0.44 (0.27-0.72)**	42.5	0.40 (0.25-0.65)***
55-64	41.2	0.33 (0.20-0.55)***	40.8	0.27 (0.15-0.48)***	28.2	0.21 (0.12-0.39)***
**Social grade**						
AB	70.1	1.00	71.4	1.00	54.8	1.00
C1	56.1	0.54 (0.33-0.88)	61.0	0.63 (0.37-1.05)	51.1	0.86 (0.53-1.40)
C2	58.5	0.60 (0.36-0.99)*	59.8	0.60 (0.35-1.01)	54.9	1.01 (0.61-1.66)
D	57.0	0.56 (0.32-0.98)*	56.6	0.52 (0.29-0.93)*	48.2	0.77 (0.44-1.34)
E	42.0	0.31 (0.18-0.54)***	52.5	0.44 (0.23-0.83)*	45.9	0.70 (0.38-1.30)
**Employment**						
Employed	62.5	1.00	65.4	1.00	55.6	1.00
Unemployed	50.0	0.60 (0.41-0.86)**	56.1	0.67 (0.46-1.00)*	48.0	0.74 (0.50-1.08)
Other (studying/retired)	47.5	0.54 (0.28-1.04)	41.4	0.37 (0.17-0.81)*	24.1	0.25 (0.11-0.61)**
**Ethnicity**						
White	57.4	1.00	61.1	1.00	51.5	1.00
All other groups	61.2	1.17 (0.70-1.97)	65.1	1.19 (0.69-2.06)	54.0	1.11 (0.65-1.87)
**Caring responsibilities**						
No	58.1	1.00	59.0	1.00	46.4	1.00
Yes	58.4	1.01 (0.72-1.41)	63.8	1.22 (0.85-1.77)	55.4	1.43 (1.00-2.05)
**Screening status**						
Intender	57.3	1.00	62.4	1.00	56.0	1.00
Maintainer	58.2	1.04 (0.75-1.44)	61.0	0.94 (0.66-1.34)	48.9	0.75 (0.53-1.07)
**Practical barriers**						
0 barriers	47.7	1.00	50.6	1.00	40.4	1.00
1 barrier	58.4	1.54 (1.03-2.31)*	61.3	1.54 (1.00-2.40)	51.2	1.55 (1.00-2.41)*
2 barriers	64.7	2.01 (1.30-3.11)**	68.9	2.17 (1.34-3.50)**	59.8	2.20 (1.38-3.51)**
3 or more barriers	64.2	2.32 (1.35-4.01)**	73.3	2.69 (1.48-4.87)**	64.0	2.63 (1.49-4.62)**

Note. OR= unadjusted odds ratio; CI= confidence interval; *p<0.05, **p<0.01, ***p<0.001, [a] participants with no smartphone removed from analyses (*n* = 81)

For peer review only - http://bmjopen.bmj.com/site/about/guidelines.xhtml

Contributorship statement

MR (Conceptualisation; Data analysis; Project administration; Writing – original draft; Writing – review & editing)

JW (Conceptualisation; Supervision; Writing – review & editing)

LM (Conceptualisation; Data analysis; Supervision; Writing – review & editing)

All authors approved the final manuscript as submitted.

Competing interests

The authors have no competing interests to declare.

Funding

MR, JW and LM are supported by a Cancer Research UK Career Development Fellowship awarded to JW (C7492/A17219

Data sharing statement

Data used and analysed in the study are available from the corresponding author on request (l.marlow@ucl.ac.uk).

For peer review only

References

1. NHS. Cervical Screening Programme, England 2016-17, National Statistics. In: Screening & Immunisations Team ND, (ed.). 2017.

2. Landy R, Pesola F, Castanon A, et al. Impact of cervical screening on cervical cancer mortality: estimation using stage-specific results from a nested case-control study. *British journal of cancer* 2016; 115: 1140-1146. 2016/10/26. DOI: 10.1038/bjc.2016.290.

3. Waller J, Jackowska M, Marlow L, et al. Exploring age differences in reasons for nonattendance for cervical screening: a qualitative study. *BJOG : an international journal of obstetrics and gynaecology* 2012; 119: 26-32. 2011/06/15. DOI: 10.1111/j.1471-0528.2011.03030.x.

4. Hope KA, Moss E, Redman CWE, et al. Psycho-social influences upon older women's decision to attend cervical screening: A review of current evidence. *Prev Med* 2017; 101: 60-66. 2017/05/16. DOI: 10.1016/j.ypmed.2017.05.002.

5. Douglas E, Waller J, Duffy SW, et al. Socioeconomic inequalities in breast and cervical screening coverage in England: are we closing the gap? *Journal of medical screening* 2016; 23: 98-103. 2015/09/18. DOI: 10.1177/0969141315600192.

6. Spencer AM, Roberts SA, Brabin L, et al. Sociodemographic factors predicting mother's cervical screening and daughter's HPV vaccination uptake. *Journal of epidemiology and community health* 2014; 68: 571-577. 2014/02/26. DOI: 10.1136/jech-2013-202629.

7. Sutton S and Rutherford C. Sociodemographic and attitudinal correlates of cervical screening uptake in a national sample of women in Britain. *Social Science & Medicine* 2005; 61: 2460-2465. DOI: https://doi.org/10.1016/j.socscimed.2005.07.017.

8. Young B, Bedford L, Kendrick D, et al. Factors influencing the decision to attend screening for cancer in the UK: a meta-ethnography of qualitative research. *Journal of public health (Oxford, England)* 2018; 40: 315-339. 2017/05/10. DOI: 10.1093/pubmed/fdx026.

9. Marlow LAV, Ferrer RA, Chorley AJ, et al. Variation in health beliefs across different types of cervical screening non-participants. *Preventive Medicine* 2018; 111: 204-209. DOI: https://doi.org/10.1016/j.ypmed.2018.03.014.

10. Waller J, Bartoszek M, Marlow L, et al. Barriers to cervical cancer screening attendance in England: a population-based survey. *Journal of medical screening* 2009; 16: 199-204. 2010/01/08. DOI: 10.1258/jms.2009.009073.

11. Marlow LAV, Chorley AJ, Haddrell J, et al. Understanding the heterogeneity of cervical cancer screening non-participants: Data from a national sample of British women. *Eur J Cancer* 2017; 80: 30-38. 2017/05/24. DOI: 10.1016/j.ejca.2017.04.017.

12. Sheeran P and Conner M. Improving the translation of intentions into health actions: The role of motivational coherence. *Health psychology : official journal of the Division of Health Psychology, American Psychological Association* 2017; 36: 1065-1073. 2017/08/22. DOI: 10.1037/hea0000553.

13. Sheeran P. Intention—Behavior Relations: A Conceptual and Empirical Review. *European review of social psychology* 2002; 12: 1-36. DOI: 10.1080/14792772143000003.

14. Weinstein ND. The precaution adoption process. *Health psychology : official journal of the Division of Health Psychology, American Psychological Association* 1988; 7: 355-386. 1988/01/01.

15. Sheeran P and Webb T. The Intention–Behavior Gap. *Social and Personality Psychology Compass* 2016; 10: 503-518. DOI: doi:10.1111/spc3.12265.

16. Everett T, Bryant A, Griffin MF, et al. Interventions targeted at women to encourage the uptake of cervical screening. *The Cochrane database of systematic reviews* 2011: CD002834. 2011/05/13. DOI: 10.1002/14651858.CD002834.pub2.

17. McDowell I, Newell C and Rosser W. Computerized reminders to encourage cervical screening in family practice. *The Journal of family practice* 1989; 28: 420-424. 1989/04/01.

18. Binstock MA, Geiger AM, Hackett JR, et al. Pap smear outreach: a randomized controlled trial in an HMO. *American journal of preventive medicine* 1997; 13: 425-426. 1998/01/07.

For peer review only - http://bmjopen.bmj.com/site/about/guidelines.xhtml

19. Duffy SW, Myles JP, Maroni R, et al. Rapid review of evaluation of interventions to improve participation in cancer screening services. *Journal of medical screening* 2017; 24: 127-145. 2016/10/19. DOI: 10.1177/0969141316664757.
20. Kitchener HC, Gittins M, Rivero-Arias O, et al. A cluster randomised trial of strategies to increase cervical screening uptake at first invitation (STRATEGIC). *Health technology assessment (Winchester, England)* 2016; 20: 1-138. 2016/09/17. DOI: 10.3310/hta20680.
21. Ipsos. Social grade: a classification tool. 2009.
22. Kerrison RS, Shukla H, Cunningham D, et al. Text-message reminders increase uptake of routine breast screening appointments: a randomised controlled trial in a hard-to-reach population. *British journal of cancer* 2015; 112: 1005-1010. 2015/02/11. DOI: 10.1038/bjc.2015.36.
23. Chorley AJ, Marlow LA, Forster AS, et al. Experiences of cervical screening and barriers to participation in the context of an organised programme: a systematic review and thematic synthesis. *Psycho-oncology* 2017; 26: 161-172. 2016/04/14. DOI: 10.1002/pon.4126.
24. Quinn M, Babb P, Jones J, et al. Effect of screening on incidence of and mortality from cancer of cervix in England: evaluation based on routinely collected statistics. *BMJ (Clinical research ed)* 1999; 318: 904-908. 1999/04/02.
25. ONS. Labour Force Survey. Office for National Statistics, 2018.
26. MORI I. The GP Patient Survey 2018. Ipsos MORI, 2018.
27. Einstein GO, McDaniel MA, Williford CL, et al. Forgetting of intentions in demanding situations is rapid. *Journal of experimental psychology Applied* 2003; 9: 147-162. 2003/10/23. DOI: 10.1037/1076-898x.9.3.147.
28. Johnson HC, Lafferty EI, Eggo RM, et al. Effect of HPV vaccination and cervical cancer screening in England by ethnicity: a modelling study. *The Lancet Public health* 2018; 3: e44-e51. 2018/01/09. DOI: 10.1016/s2468-2667(17)30238-4.

For peer review only

For peer review only - http://bmjopen.bmj.com/site/about/guidelines.xhtml

Online Supplement 1: Could changing invitation and booking processes help women translate their cervical screening intentions into action: a population-based survey of women's preferences
(Mairead Ryan, Jo Waller and Laura Marlow)

Questionnaire

Have you ever been diagnosed with cervical cancer?
1 Yes
2 No

The next few questions in this section are about cervical screening, also known as a smear or a Pap test. The NHS Cervical Screening Programme invites women in England for a cervical screening, smear or Pap test every 3 years from age 25 to age 49 and every 5 years from age 50 to age 64.Which of these statements describes whether you have had a cervical screening? If you have had a cervical screening and can't remember when, please give your best estimate.
1 I have had a test within the last 3 years
2 My last test was 3 to 5 years ago
3 My last test was more than 5 years ago
4 I have never been invited to have a test
5 I have been invited but have never had a test
6 I have had a hysterectomy so I don't need to have tests
7 I have never heard of cervical screening

Will you go for cervical screening when next invited?
1 Definitely not
2 Probably not
3 Yes, probably
4 Yes, definitely

On the next screen will be an invitation letter that the NHS sends to women to invite them to book a cervical screening appointment. Most women book cervical screening appointments at their GP practice. I would like you to imagine you received this letter in the post. Please read the letter and afterwards you will be asked some questions about your response to the letter.

* Picture of NHS screening letter shown to participant

I will now read a number of statements relating to the cervical screening letter you've just read. After each statement, please state the extent to which you agree, on a scale from 'strongly disagree 'to 'strongly agree'.

How much do you agree or disagree with this statement?
It is easy for me to find time to read a letter like this.
1 Strongly disagree
2 Disagree
3 Neither disagree or agree
4 Agree
5 Strongly agree

For peer review only

How much do you agree or disagree with this statement?
I might forget to book an appointment after reading this letter.
1 Strongly disagree
2 Disagree
3 Neither disagree or agree
4 Agree
5 Strongly agree

How much do you agree or disagree with this statement?
It is difficult for me to call my GP practice during their opening hours.
*GP opening hours provided if necessary: “Opening hours are generally between 8.00am to 6.30pm Monday to Friday”
1 Strongly disagree
2 Disagree
3 Neither disagree or agree
4 Agree
5 Strongly agree

How much do you agree or disagree with this statement?
I have access to a telephone/mobile with phone credit/minutes to call my GP practice.
1 Strongly disagree
2 Disagree
3 Neither disagree or agree
4 Agree
5 Strongly agree

How much do you agree or disagree with this statement?
It would be easy for me to find the phone number for my GP practice to contact them.
1 Strongly disagree
2 Disagree
3 Neither disagree or agree
4 Agree
5 Strongly agree

How much do you agree or disagree with this statement?
I find it takes too long to get through to a receptionist when I phone my GP practice.
1 Strongly disagree
2 Disagree
3 Neither disagree or agree
4 Agree
5 Strongly agree

We are interested in what is important to you in terms of booking a cervical screening appointment. For the following statements I read out, please state the extent to which you think each factor is important to you, on a scale from ‘very unimportant’ to ‘very important’ when booking an appointment at your GP practice.

For peer review only

How important is this when booking a cervical screening appointment at your GP practice?
Ease of booking
1 Very unimportant
2 Quite unimportant
3 Neither unimportant or important
4 Quite important
5 Very important

How important is this when booking a cervical screening appointment at your GP practice?
Cost of making booking (i.e. phone credit)
1 Very unimportant
2 Quite unimportant
3 Neither unimportant or important
4 Quite important
5 Very important

How important is this when booking a cervical screening appointment at your GP practice?
Choice of appointment times
1 Very unimportant
2 Quite unimportant
3 Neither unimportant or important
4 Quite important
5 Very important

How important is this when booking a cervical screening appointment at your GP practice?
Being able to change an appointment time/day after booking it
1 Very unimportant
2 Quite unimportant
3 Neither unimportant or important
4 Quite important
5 Very important

How important is this when booking a cervical screening appointment at your GP practice?
Privacy when booking an appointment
1 Very unimportant
2 Quite unimportant
3 Neither unimportant or important
4 Quite important
5 Very important

How important is this when booking a cervical screening appointment at your GP practice?
How long it takes to book an appointment
1 Very unimportant
2 Quite unimportant
3 Neither unimportant or important
4 Quite important
5 Very important

For peer review only

How important is this when booking a cervical screening appointment at your GP practice?
Being able to talk with a healthcare professional when booking (e.g. to ask questions about the screening before attending)

1 Very unimportant
2 Quite unimportant
3 Neither unimportant or important
4 Quite important
5 Very important

How important is this when booking a cervical screening appointment at your GP practice?
Time to the next available appointment (e.g. next available appointment isn't for two weeks)

1 Very unimportant
2 Quite unimportant
3 Neither unimportant or important
4 Quite important
5 Very important

How important is this when booking a cervical screening appointment at your GP practice?
Being able to book an appointment when the GP practice is shut (e.g. online booking)
*GP opening hours provided if necessary: "Opening hours are generally between 8.00am to 6.30pm Monday to Friday"

1 Very unimportant
2 Quite unimportant
3 Neither unimportant or important
4 Quite important
5 Very important

Again thinking about the letter you read which is sent in the post to invite women to book a cervical screening appointment. We are interested in different forms of communication to invite women to book a cervical screening appointment.

Please state the extent to which you think the following forms of communication are acceptable, on a scale from 'very unacceptable' to 'very acceptable'.

How acceptable is this form of communication when being invited to book a cervical screening appointment?
Posted letter

1 Very unacceptable
2 Quite unacceptable
3 Neither unacceptable or acceptable
4 Quite acceptable
5 Very acceptable

* If participant responded 'Quite unacceptable' or 'Very unacceptable', participant subsequently asked: Please can you tell me why you would not find receiving an invitation for a cervical screening appointment by posted letter acceptable?

For peer review only

How acceptable is this form of communication when being invited to book a cervical screening appointment?
Text message

1 Very unacceptable
2 Quite unacceptable
3 Neither unacceptable or acceptable
4 Quite acceptable
5 Very acceptable

* If participant responded 'Quite unacceptable' or 'Very unacceptable', participant subsequently asked: Please can you tell me why you would not find receiving an invitation for a cervical screening appointment by text message acceptable?

How acceptable is this form of communication when being invited to book a cervical screening appointment?
Email

1 Very unacceptable
2 Quite unacceptable
3 Neither unacceptable or acceptable
4 Quite acceptable
5 Very acceptable

* If participant responded 'Quite unacceptable' or 'Very unacceptable', participant subsequently asked: Please can you tell me why you would not find receiving an invitation for a cervical screening appointment by email acceptable?

How acceptable is this form of communication when being invited to book a cervical screening appointment?
Phone call to your mobile phone

1 Very unacceptable
2 Quite unacceptable
3 Neither unacceptable or acceptable
4 Quite acceptable
5 Very acceptable

* If participant responded 'Quite unacceptable' or 'Very unacceptable', participant subsequently asked: Please can you tell me why you would not find receiving an invitation for a cervical screening appointment by phone call to your mobile phone acceptable?

How acceptable is this form of communication when being invited to book a cervical screening appointment?
Phone call to your house landline

1 Very unacceptable
2 Quite unacceptable
3 Neither unacceptable or acceptable
4 Quite acceptable
5 Very acceptable

* If participant responded 'Quite unacceptable' or 'Very unacceptable', participant subsequently asked: Please can you tell me why you would not find receiving an invitation for a cervical screening appointment by phone call to your house landline acceptable?

For peer review only

Imagine now that different options were available to you to book a cervical screening appointment at your GP practice. Please state the extent to which you are likely to use each of the following methods to book an appointment.

How likely are you to use this method to book a cervical screening appointment at your GP practice?
Calling your GP practice

1 Very unlikely
2 Quite unlikely
3 Neither likely or unlikely
4 Quite likely
5 Very likely

How likely are you to use this method to book a cervical screening appointment at your GP practice?
Calling a 24-hour automated telephone appointment-booking system

1 Very unlikely
2 Quite unlikely
3 Neither likely or unlikely
4 Quite likely
5 Very likely

How likely are you to use this method to book a cervical screening appointment at your GP practice?
Requesting a call-back from your GP practice

1 Very unlikely
2 Quite unlikely
3 Neither likely or unlikely
4 Quite likely
5 Very likely

How likely are you to use this method to book a cervical screening appointment at your GP practice?
Booking on a website using a desktop computer/laptop

1 Very unlikely
2 Quite unlikely
3 Neither likely or unlikely
4 Quite likely
5 Very likely

How likely are you to use this method to book a cervical screening appointment at your GP practice?
Booking on a website using a smartphone

1 Very unlikely
2 Quite unlikely
3 Neither likely or unlikely
4 Quite likely
5 Very likely

For peer review only

How likely are you to use this method to book a cervical screening appointment at your GP practice? Downloading an app to a smartphone to book an appointment (you could then use the app to book other appointments at your surgery)

1 Very unlikely
2 Quite unlikely
3 Neither likely or unlikely
4 Quite likely
5 Very likely

Which of the following methods have you previously used to book an appointment at your GP practice? This could be an appointment for anything, with a GP or with a nurse.
Please select all that apply.

1 Booked in person (i.e. at the reception desk)
2 Booked by phoning the GP practice
3 Booked using a 24-hour automated telephone appointment-booking system
4 Booked online on a website
5 Booked by text-message
6 Booked using a smartphone app
7 Other
8 Don't know - someone else has always booked my appointments
9 I have never booked an appointment at my GP practice

Do you have a mobile phone?
*Description of smartphone provided if necessary; "A 'smart phone' is a mobile phone that performs many of the functions of a computer, typically having a touchscreen and Internet access"

1 Yes, a smart phone
2 Yes, but it is not a smart phone
3 No, I do not have a mobile phone

For peer review only

Online Supplement 2: Could changing invitation and booking processes help women translate their cervical screening intentions into action: a population-based survey of women's preferences

(Mairead Ryan, Jo Waller and Laura Marlow)

Table 1:

Multivariable logistic regression models of predictors of the acceptability of cervical screening invitation modalities (n=614)

	Posted letter	Text-message	Email	Mobile phone call	Landline phone call
	OR (95% CI)	OR (95% CI)	OR (95% CI)	OR (95% CI)	OR (95% CI)
**Age group**					
25-34	1.00	1.00	1.00	1.00	1.00
35-44	0.63 (0.30-1.34)	0.71 (0.40-1.25)	0.64 (0.38-1.05)	0.78 (0.46-1.32)	1.06 (0.68-1.63)
45-54	0.49 (0.22-1.06)	0.63 (0.35-1.12)	0.71 (0.42-1.20)	0.50 (0.30-0.85)*	0.70 (0.44-1.10)
55-64	1.03 (0.36-2.94)	0.29 (0.15-0.53)***	0.35 (0.19-0.63)***	0.47 (0.26-0.86)*	0.99 (0.58-1.70)
**Social grade**					
AB	1.00	1.00	1.00	1.00	1.00
C1	0.78 (0.36-1.66)	1.03 (0.58-1.82)	0.75 (0.43-1.32)	1.17 (0.71-1.94)	1.04 (0.65-1.67)
C2	3.47 (1.18-10.27)*	1.04 (0.58-1.87)	0.68 (0.39-1.21)	2.58 (1.45-4.58)**	1.82 (1.11-2.99)
D	1.75 (0.63-4.92)	1.60 (0.78-3.28)	0.90 (0.47-1.73)	1.82 (0.98-3.39)	1.67 (0.96-2.90)
E	0.88 (0.31-2.52)	0.92 (0.42-2.01)	0.38 (0.19-0.78)**	2.98 (1.35-6.58)**	1.76 (0.90-3.43)
**Employment**					
Employed	1.00	1.00	1.00	1.00	1.00
Unemployed	0.63 (0.30-1.33)	1.12 (0.65-1.94)	0.89 (0.54-1.47)	1.06 (0.63-1.79)	1.31 (0.83-2.06)
Other (studying/retired)†	-	-	-	-	-
**Ethnicity**					
White	1.00	1.00	1.00	1.00	1.00
All other groups	0.45 (0.21-0.97)*	1.60 (0.75-3.39)	2.22 (1.08-4.57)*	1.26 (0.65-2.45)	1.38 (0.79-2.42)
**Caring responsibilities**					
No	1.00	1.00	1.00	1.00	1.00
Yes	1.79 (0.96-3.23)	1.09 (0.70-1.69)	1.19 (0.79-1.80)	1.60 (1.06-2.41)*	1.20 (0.83-1.73)

Note. OR= adjusted odds ratio; CI= confidence interval; *p<0.05, **p<0.01, ***p<0.001; 'screening status' and 'practical barriers' variables not included because not significant in univariable analyses; †category not included due to insufficient cases

For peer review only

Table 2

Multivariable logistic regression models of predictors of phone-based booking preferences (n=614)

	**Calling the GP**	**Calling a 24-hour automated service**	**Requesting a call-back**
	OR (95% CI)	OR (95% CI)	OR (95% CI)
**Age group**			
25-34	1.00	1.00	1.00
35-44	0.78 (0.38-1.60)	0.68 (0.45-1.04)	0.96 (0.62-1.47)
45-54	0.70 (0.32-1.50)	0.50 (0.32-0.79)*	0.65 (0.41-1.03)
55-64	0.76 (0.31-1.83)	0.45 (0.27-0.76)**	1.19 (0.70-2.01)
**Caring responsibilities**			
No	1.00	1.00	1.00
Yes	1.11 (0.61-2.01)	0.95 (0.67-1.37)	1.82 (1.26-2.62)**
**Screening status**			
Intender	1.00	1.00	1.00
Maintainer	1.55 (0.89-2.68)	0.97 (0.70-1.36)	0.68 (0.48-0.95)*
**Practical barriers**			
0 barriers	1.00	1.00	1.00
1 barrier	1.48 (0.71-3.06)	0.90 (0.59-1.36)	0.81 (0.53-1.23)
2 barriers	1.39 (0.65-2.97)	1.26 (0.81-1.96)	1.26 (0.80-1.97)
3 or more barriers cited	0.63 (0.29-1.37)	1.63 (0.94-2.82)	1.35 (0.77-2.36)

Note. OR= adjusted odds ratio; CI= confidence interval; *p<0.05, **p<0.01, ***p<0.001; 'social grade', 'employment' and 'ethnicity' not included because not significant in univariable analyses

For peer review only

Table 3

Multivariable logistic regression models of predictors of online booking preferences (n=614)

	**Booking on a website using a desktop/laptop**	**Booking on a website using a smartphone [a]**	**Downloading an app to your smartphone [a]**
	OR (95% CI)	OR (95% CI)	OR (95% CI)
**Age group**			
25-34	1.00	1.00	1.00
35-44	0.64 (0.42-1.00)*	0.58 (0.37-0.91)*	0.50 (0.33-0.77)**
45-54	0.58 (0.36-0.93)*	0.33 (0.20-0.53)***	0.29 (0.18-0.47)***
55-64	0.34 (0.20-0.58)***	0.19 (0.11-0.33)***	0.17 (0.10-0.31)***
**Social grade**			
AB	1.00	1.00	1.00
C1	0.52 (0.31-0.86)*	0.61 (0.36-1.02)	0.88 (0.53-1.45)
C2	0.55 (0.33-0.93)*	0.43 (0.25-0.73)**	0.76 (0.46-1.28)
D	0.50 (0.78-0.89)*	0.43 (0.24-0.78)**	0.66 (0.37-1.18)
E	0.35 (0.18-0.69)**	0.42 (0.21-0.84)*	0.63 (0.31-1.28)
**Employment**			
Employed	1.00	1.00	1.00
Unemployed	0.80 (0.51-1.24)	0.72 (0.46-1.14)	0.76 (0.48-1.20)
Other (studying/retired)	0.82 (0.39-1.73)	0.54 (0.25-1.17)	0.35 (0.14-0.85)*
**Practical barriers**			
0 barriers	1.00	1.00	1.00
1 barrier	1.46 (0.96-2.23)	1.20 (0.78-1.85)	1.17 (0.76-1.81)
2 barriers	1.73 (1.10-2.73)*	1.82 (1.13-1.91)*	1.77 (1.11-2.82)*
3 or more barriers	2.16 (1.22-3.82)**	2.59 (1.43-4.69)**	2.57 (1.45-4.56)**

Note. OR= adjusted odds ratio; CI= confidence interval; *p<0.05, **p<0.01, ***p<0.001; [a] participants with no smartphone removed from analyses (*n* = 81); 'ethnicity', 'caring responsibilities' and 'screening status' not included because not significant in univariable analyses

For peer review only

Online Supplement 3: Could changing invitation and booking processes help women translate their cervical screening intentions into action: a population-based survey of women's preferences
(Mairead Ryan, Jo Waller and Laura Marlow)

Open responses provided for citing invitation method as unacceptable

Invitation mode	Unacceptable (n)	Reasons for being unacceptable
Posted letter	12	Don't open post/might miss the letter/no time to read letter (n=4)<br>Receive letter too late (n=2)<br>Letter could be lost in the post (n=2)<br>Other (n=4)<br>• Would forget (n=1)<br>• Environmental concerns (n=1)<br>• Waste of time (n=1)<br>• No reason provided (n=1)
Text-message	67	Privacy concerns (n=21)<br>Easy to miss it/may not read message (n=9)<br>Reason not provided (i.e. N/A) (n=9)<br>Doesn't have or use mobile (n=7)<br>Impersonal (n=6)<br>Could change number (n=4)<br>Prefer a letter/phone call (n=4)<br>Not reliable source/unprofessional (n=3)<br>Would forget/not act on it (n=2)<br>Other (n=2)<br>• Don't know (n=1)<br>• They can text me but I don't want to text them (n=1)
Email	94	Would be lost in other emails/would not be seen (n=38)<br>No email/doesn't use email/no internet/no computer (n=17)<br>Privacy concerns (n=12)<br>Reason not provided (i.e. N/A) (n=12)<br>Prefer phone or letter (n=5)<br>Would forget/not act on it (n=2)<br>Impersonal/rude (n=2)<br>Other (n=6)<br>• Not timely (n=1)<br>• Intrusive (n=1)<br>• Not normal (n=1)<br>• No reason (n=1)<br>• Not keen (n=1)<br>• Doesn't trust source (n=1)
Mobile phone	90	Would not be able to pick up/would miss call (n=33)<br>Privacy concerns (n=22)

For peer review only

call		Would prefer in writing/a letter (n=10)<br>Reason not provided (i.e. N/A) (n=8)<br>Would not know number – so would not answer call (n=5)<br>No mobile (n=2)<br>Would forget (n=2)<br>Too many phone calls (n=2)<br>Other (n=6)<br>• Don't like idea (n=1)<br>• Talking takes too much time (n=1)<br>• Need time to think (n=1)<br>• Impersonal (n=1)<br>• People change phone number (n=1)<br>• Don't like calls (n=1)
Landline phone call	129	No landline (n=39)<br>Would miss call/out of the house during the day (n=31)<br>Privacy concerns (n=24)<br>No reason provided (i.e. N/A) (n=12)<br>Feels intrusive (n=5)<br>Prefer in writing/letter (n=5)<br>Don't want phone call (n=4)<br>Not reliable source (n=3)<br>Other (n=6)<br>• Impersonal (n=1)<br>• "Better with working" (n=1)<br>• Unnecessary (n=1)<br>• Unknown number (n=1)<br>• Want time to think (n=1)<br>• Doesn't matter either way (n=1)

For peer review only

STROBE 2007 (v4) Statement—Checklist of items that should be included in reports of *cross-sectional studies*

Section/Topic	Item #	Recommendation	Reported on page #
**Title and abstract**	1	(*a*) Indicate the study's design with a commonly used term in the title or the abstract	2
		(*b*) Provide in the abstract an informative and balanced summary of what was done and what was found	2
**Introduction**			
Background/rationale	2	Explain the scientific background and rationale for the investigation being reported	3-4
Objectives	3	State specific objectives, including any prespecified hypotheses	4
**Methods**			
Study design	4	Present key elements of study design early in the paper	4
Setting	5	Describe the setting, locations, and relevant dates, including periods of recruitment, exposure, follow-up, and data collection	4
Participants	6	(*a*) Give the eligibility criteria, and the sources and methods of selection of participants	4-5
Variables	7	Clearly define all outcomes, exposures, predictors, potential confounders, and effect modifiers. Give diagnostic criteria, if applicable	5-6
Data sources/ measurement	8*	For each variable of interest, give sources of data and details of methods of assessment (measurement). Describe comparability of assessment methods if there is more than one group	5-6
Bias	9	Describe any efforts to address potential sources of bias	4
Study size	10	Explain how the study size was arrived at	5
Quantitative variables	11	Explain how quantitative variables were handled in the analyses. If applicable, describe which groupings were chosen and why	6
Statistical methods	12	(*a*) Describe all statistical methods, including those used to control for confounding	6
		(*b*) Describe any methods used to examine subgroups and interactions	6
		(*c*) Explain how missing data were addressed	
		(*d*) If applicable, describe analytical methods taking account of sampling strategy	
		(*e*) Describe any sensitivity analyses	
**Results**			

For peer review only

Participants	13*	(a) Report numbers of individuals at each stage of study—eg numbers potentially eligible, examined for eligibility, confirmed eligible, included in the study, completing follow-up, and analysed	6
		(b) Give reasons for non-participation at each stage	n/a
		(c) Consider use of a flow diagram	
Descriptive data	14*	(a) Give characteristics of study participants (eg demographic, clinical, social) and information on exposures and potential confounders	6-7
		(b) Indicate number of participants with missing data for each variable of interest	
Outcome data	15*	Report numbers of outcome events or summary measures	
Main results	16	(*a*) Give unadjusted estimates and, if applicable, confounder-adjusted estimates and their precision (eg, 95% confidence interval). Make clear which confounders were adjusted for and why they were included	7-8, 12-15
		(*b*) Report category boundaries when continuous variables were categorized	5-6
		(*c*) If relevant, consider translating estimates of relative risk into absolute risk for a meaningful time period	n/a
Other analyses	17	Report other analyses done—eg analyses of subgroups and interactions, and sensitivity analyses	
**Discussion**			
Key results	18	Summarise key results with reference to study objectives	8-10
Limitations	19	Discuss limitations of the study, taking into account sources of potential bias or imprecision. Discuss both direction and magnitude of any potential bias	9-10
Interpretation	20	Give a cautious overall interpretation of results considering objectives, limitations, multiplicity of analyses, results from similar studies, and other relevant evidence	10
Generalisability	21	Discuss the generalisability (external validity) of the study results	9-10
**Other information**			
Funding	22	Give the source of funding and the role of the funders for the present study and, if applicable, for the original study on which the present article is based	1

*Give information separately for cases and controls in case-control studies and, if applicable, for exposed and unexposed groups in cohort and cross-sectional studies.

Note: An Explanation and Elaboration article discusses each checklist item and gives methodological background and published examples of transparent reporting. The STROBE checklist is best used in conjunction with this article (freely available on the Web sites of PLoS Medicine at http://www.plosmedicine.org/, Annals of Internal Medicine at http://www.annals.org/, and Epidemiology at http://www.epidem.com/). Information on the STROBE Initiative is available at www.strobe-statement.org.

For peer review only

BMJ Open

Could changing invitation and booking processes help women translate their cervical screening intentions into action? A population-based survey of women's preferences in Great Britain.

Journal:	*BMJ Open*
Manuscript ID	bmjopen-2018-028134.R1
Article Type:	Research
Date Submitted by the Author:	10-Apr-2019
Complete List of Authors:	Ryan, Mairead; University College London Institute of Epidemiology and Health Care, Department of Behavioural Science and Health;<br>Waller, Jo; UCL, Epidemiology and Public Health<br>Marlow, Laura; University College London
**Primary Subject Heading**:	Epidemiology
Secondary Subject Heading:	Epidemiology, Communication
Keywords:	cervical cancer screening, non-participants, interventions, age, screening status, uptake

SCHOLARONE™
Manuscripts

Could changing invitation and booking processes help women translate their cervical screening intentions into action? A population-based survey of women's preferences in Great Britain.

Mairead Ryan, MSc

Jo Waller, PhD

Laura A.V Marlow, PhD

Cancer Communication and Screening Group, Research Department of Behavioural Science and Health, University College London, Gower Street, London, UK

Corresponding author contact details: Laura Marlow, l.marlow@ucl.ac.uk, 020 7679 1798.

MR, JW and LM are supported by a Cancer Research UK Career Development Fellowship awarded to JW (C7492/A17219).

For peer review only

Abstract

Objectives: Many women who do not attend screening intend to go, but do not get around to booking an appointment. Qualitative work suggests these ‘intenders’ face more practical barriers to screening than women who are up-to-date (‘maintainers’). This study explored practical barriers to booking a screening appointment and preferences for alternative invitation and booking methods that might overcome these barriers.

Design: A cross-sectional survey was employed.

Setting: Great Britain

Participants: Women aged 25 to 64, living in Great Britain who intended to be screened but were overdue (‘intenders’, n=255) and women who were up-to-date with screening (‘maintainers’, n=359)

Results: ‘Intenders’ reported slightly more barriers than ‘maintainers’ overall (mean = 1.36 vs 1.06, t=3.03, p <0.01) and were more likely to think they might forget to book an appointment (Odds ratio=2.87, 95% confidence interval: 2.01-4.09). Over half of women said they would book on a website using a smartphone (62%),a computer (58%) or via an app (52%). Older women and women from lower social grades were less likely to say they would use online booking methods (all ps<.05). Women who reported two or more barriers were more likely to say they would use online booking than women who reported none (ps<.01).

Conclusions: Women who are overdue for screening face practical barriers to booking appointments. Future interventions may assess the efficacy of changing the architecture of the invitation and booking system. This may help women overcome logistical barriers to participation and increase coverage for cervical screening.

Strengths and limitations of this study

- This was the first study to break down the invitation and booking process into its component parts, identifying barriers at each stage of the process and alternative booking options which may help women to overcome these barriers

For peer review only - http://bmjopen.bmj.com/site/about/guidelines.xhtml

- Women were purposely recruited to be up-to-date and overdue, however response rate was not recorded.
- The practical barriers cited in this study relate to the booking process and are not exhaustive of all practical barriers to cervical screening. They may not reflect booking processes in other countries.

Introduction

Cervical screening programmes are designed to reduce the incidence and mortality rate of cervical cancer.[1] In Great Britain all eligible women aged 25 to 64 registered with a GP are invited to be screened for the presence of abnormal cell changes in the cervix, which could, if undetected and untreated, develop into cervical cancer. The efficacy of the programme has been widely acknowledged,[2] however the success of any screening programme is dependent on good coverage. In 2017, coverage (i.e. the percentage of eligible women recorded as adequately screened) was 72%, well below the national target of 80% and in keeping with a trend of decreasing screening coverage.

Reasons for screening non-attendance are complex and differ depending on socio-demographic factors such as age, socio-economic status and marital status.[3-6] Emotional barriers including embarrassment, fear of pain and negative experiences are often reported, particularly in qualitative studies.[7-9] While these barriers undoubtedly need to be addressed, practical barriers have been found to be more predictive of screening status than emotional barriers.[10] Recent research showed that over half of women overdue for cervical screening have positive intentions to attend.[11] While this is encouraging, intentions are frequently not translated into action.[12, 13]

Weinstein used a 'messy desk' analogy to help explain the problem of translating intentions into action.[14] He proposed that we do not carry out errands in a logical sequence, but rather in a haphazard manner, acting on 'to-do' list items when we feel pressure, when items need to be actioned quickly, when prompted or because of personal preference. More recently, Sheeran and Webb identified three key problems (or 'TRIALS') people might encounter when trying to realise their intentions; i) they fail to get started (e.g. forget to act or miss an opportunity to act), ii) they fail to keep the goal on track (fail to monitor the goal, face competing thoughts or distractions) and iii) they fail to close (don't quite meet the goal).[15]

Women receive a posted letter inviting them to book a screening appointment. The letter states the recipient "can make an appointment for cervical screening by phoning *(their)* GP surgery". GP surgery hours generally coincide with 'normal' working hours, presenting several practical barriers for women who are in full-time employment or who have caring responsibilities, both in terms of

For peer review only - http://bmjopen.bmj.com/site/about/guidelines.xhtml

phoning and attending a GP surgery. Previous research has identified that many women find the booking process arduous and inflexible.[3]

Few studies have assessed alternative methods of inviting women for cervical screening.[16] The most recent Cochrane review of interventions to improve uptake [16] reported two studies from the 1980s and 90s, which found that participants who received a telephone invitation were significantly more likely to attend than those who received a letter.[17, 18] Studies which have examined the utility of more recent technological developments to invite women are lacking.[19] There is also a paucity of literature concerning alternative booking methods for cervical screening, most likely due to limited booking options being available until recently. One trial investigated the efficacy of online booking among first time invitees.[20] The intervention group booked slightly more appointments within three months (2.18% higher than the control group) however, this was not statistically significant.[20] The authors noted that the way the online booking system was offered could account for the lack of support (in a letter participants were asked to visit a website to book at one of three sexual health clinics). Hence, other forms of online booking may be desirable to women.

New technologies offer opportunities for editing the architecture of the invitation and booking system in ways that may help to overcome some of the challenges women face between forming a positive intention and translating this into behaviour, as highlighted in the TRIALS model. For example, online booking methods may reduce the likelihood that women would fail to get started, given that opportunities to act (i.e. book an appointment) are not limited to GP practice opening hours. The present study explored practical barriers to booking an appointment among two groups: women who are up-to-date with screening ('maintainers') and women who intend to be screened but are currently overdue ('intenders'). Our aim was to examine between-group differences which may account for this intention-behaviour gap among 'intenders'. We also assessed invitation and booking preferences and explored whether these might help to overcome practical barriers.

Methods

Participants

Participants were recruited by Kantar TNS UK as part of their omnibus survey. The TNS omnibus survey recruits a new sample of 2000-4000 men and women living in Great Britain on a weekly basis and asks questions on a range of topics commissioned by external companies. Recruitment uses random location sampling to identify areas for sampling participants using the 2011 Census and the Postcode Address File. Recruiters visit homes in the identified areas and knock on doors asking those who answer to participate. All interviews are conducted in English. Quotas are set at each location for age, gender, working status, and presence of children in the household.

For peer review only - http://bmjopen.bmj.com/site/about/guidelines.xhtml

For peer review only

Women who were eligible for cervical screening and had not previously been diagnosed with cervical cancer, were asked to report their past attendance at cervical screening and future intention to attend (see Online Supplement 1). Responses to these questions were used to classify women as 'intenders' (intended to be screened but were currently overdue), 'maintainers' (up-to-date with screening and intending to go in the future) or 'other' (never heard of screening, never been invited, decided not to be screened). A sample of 600 women was expected to allow us to establish a significant difference of 5% between preferred booking options in the two groups of attenders within +/- 8% with 95% confidence.

Procedure

Ethical approval was granted by University College London Research Ethics Committee (reference: 10353/003). Data were collected between April and May 2018. Face-to-face computer-assisted personal interviews were used to collect data. Kantar TNS provided anonymised data to UCL for analysis.

Measures

Invitation preferences: Participants were asked whether several different modes of communication were acceptable to them as a means of being invited to book a cervical screening appointment (see Online Supplement 1). Participants' responses were recoded as 'acceptable' (if they responded quite acceptable/very acceptable) or 'unacceptable' (if they responded quite unacceptable/very unacceptable/neither unacceptable nor acceptable/don't know/not applicable). Participants who responded quite/very unacceptable were asked to explain why (open response).

Practical barriers to booking an appointment: Participants were asked to respond to a list of barriers, which were based on the key problems outlined in the TRIALS model.[15] Statements addressing the key problem of 'failing to get started' included 'It is easy for me to find time to read a letter like this' and 'I might forget to book an appointment after reading this letter'. Statements addressing 'failing to keep the goal on track' included 'It is difficult for me to call my GP practice during their opening hours' and 'I find it difficult to get through to a receptionist when I phone my GP practice'. Women were then asked to state which booking attributes were important to them, the aim of which was to address factors that might influence 'failure to close' (i.e. being able to book the appointment).

Booking preferences: Participants were asked to indicate how likely they would be to use different booking methods. The feasibility of these methods were informally discussed with stakeholders from the NHS national screening programme and with representatives from a technology company, who develop methods of improving access to healthcare. Participants' responses were recoded as 'likely to use' a method (if they responded quite likely/very likely) or 'not likely to use' (if they responded

For peer review only - http://bmjopen.bmj.com/site/about/guidelines.xhtml

quite unlikely/very unlikely/neither unlikely nor likely/don't know /not applicable). Participants were also asked to indicate which booking methods they had used in the past for any GP appointment.

Socio-demographic and background factors: Data regarding age, ethnicity, education level, employment status, marital status, social grade, child/carer responsibilities and smartphone ownership were also collected. Social grade is determined by the occupation of the Chief Income Earner in the household and is classified as follows: AB managerial/professional; C1 supervisory; C2 skilled manual; D semi-skilled/unskilled manual; E casual workers/unemployed.[21]

Patient and Public Involvement Statement

The study was supported by a PPI group who provided input into the contents of the survey. A group of 10 screening-eligible women were invited to guide and refine the survey questions. Women who were both up-to-date and overdue were represented in the group. The group helped to establish the perceived difficulty of the questions (e.g. unknown terms, ambiguous concepts, long and overly complex questions) and omissions from the survey. The questions and response options were tailored based on feedback provided by this PPI group.

Analyses

All analyses were conducted using IBM SPSS version 22. Chi-squared analyses were conducted to test for significant differences in participant demographics between 'Intenders' and 'Maintainers'. Descriptive statistics were conducted to assess booking history and smartphone/mobile phone ownership across all participants. For each of the six practical barrier statements, any positively-framed items were reverse-scored so that a higher score was indicative of a barrier for all items. Total practical barrier scores were created by allocating a score of 1 for each barrier statement that a participant 'agreed' or 'strongly agreed' with and adding these together (possible range 0-6). Independent samples t-tests were conducted to assess differences in the mean barriers scores between 'intenders' and 'maintainers'. A series of binary logistic regressions were then conducted to assess the associations between endorsing each barrier/booking attribute and the unadjusted odds for being an 'intender' (versus a 'maintainer'). A series of univariable logistic regressions were conducted to explore whether socio-demographic factors, screening status and number of practical barriers reported were associated with invitation and booking preferences. Multivariable logistic regressions are presented as supplementary material.

Results

Sample characteristics

For peer review only - http://bmjopen.bmj.com/site/about/guidelines.xhtml

2509 eligible respondents (i.e. women aged 25-64 years) completed the Kantar TNS survey. After exclusions, 1548 (78%) were up-to-date and 445 (22%) were overdue for screening. Our questions on invitation and booking preferences for cervical screening were asked to all women who were classified as 'intenders' and women who were classified as 'maintainers' in week 1. See Online Supplement 2 for survey inclusion flow diagram.

Sample characteristics for participants classified as 'intenders' (n=255) and 'maintainers' (n=359) are presented in Table 1. Mean age was 41.69 years (SD=10.84, range: 25-64 years), the majority self-identified as White (89%), were employed (64%), married or co-habiting (67%) and had regular caring responsibilities (i.e. for children/parents; 63%). 'Intenders' (mean=39.41; SD=9.94) were significantly younger than 'maintainers' (mean=43.31; SD=11.16); $t(612)=4.47$, $p<.001$.

The majority of women had previously booked by phoning the practice (89%), over one-third had booked in person (39%) and 14% had booked on a website. 'Maintainers' were significantly more likely to have previously booked on a website than 'intenders' (see Table 1). The majority of participants had a smartphone (87%), fewer women had a mobile phone which was not a smartphone (11%) and a small minority had no mobile phone (2%).

Practical barriers to appointment booking and desired attributes

Over two-thirds of women reported one or more barriers to booking (69%); mean number of reported barriers was 1.21 (SD=1.06). 'Intenders' (mean=1.36; SD=1.06) reported slightly more barriers than 'maintainers' overall (mean=1.10; SD=1.04; $t(612)=3.03$, $p<0.01$). The most commonly endorsed barrier was 'I find it difficult to get through to a receptionist when I phone my GP practice' (50% of participants 'strongly agreed' or 'agreed'), followed by 'It is difficult for me to call my GP practice during their opening hours' (31%) and 'I might forget to book an appointment after reading this letter' (31%). Practical barriers to appointment booking and booking characteristics considered to be important are outlined in Table 2. The 'intenders' group were significantly more likely to endorse the statement 'I might forget to book an appointment after reading this letter' than 'maintainers'. 'Intenders' were also more likely to state 'How long it takes to book the appointment' was important to them than 'maintainers'.

Invitation preferences

Posted letters emerged as the most acceptable invitation mode followed by text-message (see Table 3). Socio-demographic predictors of the acceptability of each modality are shown in Table 3. Text-message, email and mobile call invitations were less acceptable to women aged 55-64; these associations remained significant in multivariable analyses (see Online Supplement 3). Mobile and landline call invites were more acceptable to women from lower socio-economic backgrounds and

For peer review only - http://bmjopen.bmj.com/site/about/guidelines.xhtml

this remained significant in multivariable analyses for mobile invites. Reasons for considering invitation modes as unacceptable are provided in Online Supplement 4; fears about missing a phone call/email or text and privacy concerns were commonly cited. Many participants also reported they had no landline phone.

Phone-based booking preferences

Most women said they were likely to book by phoning their GP practice (90%; see Table 4). Older women were significantly less likely to say they would call a 24-hour automated service than women aged 25-34 (41% vs 61%). Women with caring responsibilities were more likely to say they would request a call-back compared to women with no caring responsibilities (62% vs 49%). 'Maintainers' were less likely to say they would request a call-back than 'intenders' (63% vs 53%). These associations remained significant in multivariable analyses. Women who cited three or more barriers were more likely to say they would call a 24-hour automated service but this association was not significant in multivariable analyses.

Online booking preferences

Booking on a website using a smartphone (62%) was the preferred online booking method (see Table 5). Older women (55-64 years) were less likely to say they would book online than younger women (25-34 years). Women in lower social grades were less likely than women in the highest grade to state they would book on a website, either using a desktop or smartphone. Participants who were studying or retired were less likely than those employed to say they would book online (either on a website using a smartphone: 41% vs 65%, or through an app: 24% vs 56%). Women who reported two or more barriers were more likely to report that they would use all online booking methods compared to women who reported no barriers (see Table 5). Age, social grade, employment status and number of barriers remained significant in multivariable analyses.

Discussion

This study examined women's practical barriers to booking a cervical screening appointment and assessed whether invitation and booking preferences are associated with reported barriers, socio-demographic factors and screening status. Approximately one-third of all women reported that it is difficult to phone their GP practice within opening hours and half reported that it is difficult to get through to a receptionist. Although the survey found that 'intenders' experience slightly more practical barriers to screening than 'maintainers', endorsement of barriers across the sample suggests that both groups need more support in booking an appointment.

For peer review only - http://bmjopen.bmj.com/site/about/guidelines.xhtml

'Intenders' were more likely to report that they would forget to book an appointment after reading the screening letter than 'maintainers'. This key problem relates to a 'failure to get started', which is a first barrier people face between forming an intention and translating this into behaviour.[15] Written reminders are an integral part of the screening programme and there is good evidence to show these improve uptake,[16] but in their current format these reminders do not seem to help all women to remember to book their appointment. Future research might explore methods of increasing the salience of cervical screening among invitees (e.g. employing implementation intentions).[22] The use of text-message reminders has shown promise in other screening contexts.[23] 'Intenders' were also more likely to say that the length of time needed to book an appointment was important to them. Since all women eligible for cervical screening fall within the working age population, and GP opening hours generally overlap with working hours, it is likely this cohort face competing obligations,[24] and, as a result 'fail to keep their goal on track'.[15] The rate of female employment (16 to 64 years) has increased from 62.2% in 1994, when coverage was high (85%; five yearly coverage for women aged 20 to 64)[25] to 70.5% in 2017.[26] Alternative booking methods may provide more flexibility.

Women who reported more barriers showed greater interest in using alternative booking methods. Specifically, participants who reported two or more barriers were more likely to say that they would book on a website or through an app. This is perhaps not surprising since these methods overcome the most common practical barriers highlighted by participants, including, difficulty getting through to a receptionist and difficulty calling the practice during opening hours; hence they 'fail to close'. Nevertheless, while 24-hour automated services offers these same advantages, consistent with previous national surveys,[27] fewer women reported that they would use this booking option. Online booking services are already set up in the majority of GP practices across England for GP appointments, however a national survey found that over 40% of patients are unaware if there are online booking services at their GP practice.[28] Hence, signposting online booking services, if available for nurse appointments, to groups of the screening-eligible population (i.e. younger women who are more likely to be 'intenders') may be an effective means of increasing uptake. This survey suggests that there are likely to be age and socio-economic inequalities in the use of online bookings. For example, women aged 45-54 years and women age 55-64 showed less interest in using online booking methods. Thus, ensuring that traditional telephone booking options remains available is important.

For peer review only - http://bmjopen.bmj.com/site/about/guidelines.xhtml

Previous research has found that it is very difficult for individuals to maintain intentions after even very brief periods of time (less than one minute), especially in circumstances where there are competing tasks.[29] Unlike posted letters, which may not be read until the end of the day, text-messages can be delivered at a time when GP practices are open, so women can act immediately on their intentions to book an appointment. Given that text-message invites were considered acceptable to the majority of women across all socio-demographic backgrounds, and have previously been found to be effective in increasing uptake for other national screening programmes,[23] the use of text-message invitations may be a worthwhile intervention to explore. Text-messages within the cervical screening programme have, thus far, been introduced as a booking reminder, rather than as a stand-alone invitation, which the current study did not specify. Some participants shared concerns that they may miss the message; outlining that text-messages would be used as a supplemental invitation may have further increased acceptability within the sample. Further research is needed to explore methods of overcoming privacy concerns associated with text-messages, which some of the participants raised.

This study had some limitations. We were unable to collect data on women who elected not to participate in the study. Hence the response rate and differences between respondents and non-respondents could not be determined. Women in the survey tended to be slightly less deprived and were less likely to be from ethnic minority backgrounds than the population represented in the most recent Census.[30] This suggests there was a slight bias in participation. This survey was also conducted in English and therefore non-English speakers were not represented. Given ethnic disparities in screening attendance in England,[31] more work is needed to explore methods of overcoming practical barriers to screening for ethnic minority women.

Participation in screening was self-reported. Previous research has found that women tend to over-report their participation in cervical screening programmes,[32, 33] thus some of the women classified as 'maintainers' may actually be overdue for screening. Furthermore, although this study explored practical barriers to appointment-booking based on the TRIALS model,[15] several other practical barriers were not assessed. For example, previous research has found that 'intenders' are more likely to have children under the age of five;[11] childcare may be an additional practical barrier to screening. Thus the barriers cited in this study are not exhaustive of all practical barriers to screening for women. In addition, the study was designed to reflect the current booking process for cervical screening in Great Britain. While there may be parallels with other countries that have call-recall programs with paper-based invitations and self-booked appointments in primary care, the findings

For peer review only - http://bmjopen.bmj.com/site/about/guidelines.xhtml

may not be generalisable to screening programmes in other countries, where the invitation and booking approach differs.

Nevertheless, this was the first study to assess preferences for booking a screening appointment in Great Britain, an important first step in the development of trialling and implementing any of these changes. The invitation and booking process was broken down to identify barriers at each stage and associated preferences which may help women to overcome such barriers. The lack of differences by screening status suggests that changing the architecture should not deter ‘maintainers’ from participation. Future interventions may assess the efficacy of i) signposting invitees to online booking services, ii) text-messages which are delivered during GP opening hours and iii) sending reminders to reduce the likelihood of forgetting to book an appointment. Implementation research will further determine how best to introduce such changes to the screening infrastructure.

For peer review only

For peer review only - http://bmjopen.bmj.com/site/about/guidelines.xhtml

Table 1:

Sample Characteristics (n=614)

	Overall (n=614) N (%)	Maintainers (n=359) N (%)	Intenders (n=255) N (%)	Difference between maintainers and intenders Chi Square (df), P-value
**Age (years)**				14.16 (3), <.001
25-34	192 (31.3)	103 (28.7)	89 (34.9)	
35-44	183 (29.8)	95 (26.5)	88 (34.5)	
45-54	137 (22.3)	88 (24.5)	49 (19.2)	
55-64	102 (16.6)	73 (20.3)	29 (11.4)	
**Ethnicity**				0.10 (1), 0.76
Any white	547 (89.1)	321 (89.4)	226 (88.6)	
All other groups	67 (10.9)	38 (10.6)	29 (11.4)	
**Education level**				2.12 (4), 0.71
GCSE or below	180 (29.3)	108 (30.1)	72 (28.2)	
A level or equivalent	71 (11.6)	45 (12.5)	26 (10.2)	
College qualification	115 (18.7)	62 (17.3)	53 (20.8)	
Degree or higher	213 (34.7)	125 (34.8)	88 (34.5)	
Other	35 (5.7)	19 (5.3)	16 (6.3)	
**Employment status**				3.19 (2), 0.20
Employed (full-time/part-time)	392 (63.8)	234 (65.2)	158 (62.0)	
Unemployed	182 (29.6)	98 (27.3)	84 (32.9)	
Other (studying/retired)	40 (6.5)	27 (7.5)	13 (5.1)	
**Marital status**				2.89 (2), 0.24
Single	129 (21.0)	67 (18.7)	62 (24.3)	
Married/living as married	413 (67.3)	249 (69.4)	164 (64.3)	
Widowed/divorced/separated	72 (11.7)	43 (12.0)	29 (11.4)	
**Parent/carer role**				0.62 (0.45), 0.43
Yes	387 (63.0)	221 (61.6)	166 (65.1)	
No	222 (36.2)	134 (37.3)	88 (34.5)	
**Social status**				7.93 (4), 0.09
AB (highest)	134 (21.8)	90 (25.1)	44 (17.3)	
C1	157 (25.6)	88 (24.5)	69 (27.1)	
C2	142 (23.1)	84 (23.4)	58 (22.7)	
D	93 (15.1)	54 (15.0)	39 (15.3)	
E (lowest)	88 (14.3)	43 (12.0)	45 (17.6)	
**Booking history (Yes/No)**				
Phoned the practice	545 (88.8)	316 (88.0)	229 (89.8)	0.47 (1), 0.49
At reception (in person)	240 (39.1)	145 (40.4)	95 (37.3)	0.62 (1), 0.43
24-hr automated service	23 (3.7)	14 (3.9)	9 (3.5)	0.06 (1), 0.81
Text-message	7 (1.1)	4 (1.1)	3 (1.2)	0.01 (1), 0.94
Website	85 (13.8)	60 (16.7)	25 (9.8)	5.97 (1), <.05
Smartphone app	23 (3.7)	15 (4.2)	8 (3.1)	0.45 (1), 0.50
**Phone ownership**				0.72 (2), 0.70
Smartphone	533 (86.8)	315 (87.7)	218 (85.5)	
Non-smartphone mobile	67 (10.9)	36 (10.0)	31 (12.2)	
No phone	14 (2.3)	8 (2.2)	6 (2.4)	

For peer review only

For peer review only - http://bmjopen.bmj.com/site/about/guidelines.xhtml

Table 2:

Practical barriers to appointment booking and booking characteristics considered to be important (n=614)

	All (*n=614*) N (%)	'Maintainers' (*n=359*) N (%)	'Intenders' (*n=255*) N (%)	OR for being an 'intender' (95% CI)
**Practical barriers to booking screening (% agree/strongly agree)**				
It is (*not*) easy for me to find time to read a letter like this	25 (4.1)	15 (4.2)	10 (3.9)	0.94 (0.41-2.12)
I might forget to book an appointment after reading this letter	187 (30.5)	76 (21.2)	111 (43.5)	2.87 (2.01-4.09)**
It is difficult for me to call my GP practice during their opening hours	192 (31.3)	108 (30.1)	84 (32.9)	1.14 (0.81-1.61)
I (*do not*) have access to a telephone/mobile with phone credit/minutes to call my GP practice	13 (2.1)	8 (2.2)	5 (2.0)	0.88 (0.28-2.71)
I would (*not*) find it easy to find the phone number for my GP practice to contact them	19 (3.1)	11 (3.1)	8 (3.1)	1.01 (0.41-2.59)
I find it difficult to get through to a receptionist when I phone my GP practice	306 (49.8)	177 (49.3)	129 (50.6)	1.05 (0.76-1.45)
**Booking attributes (% saying quite/very important)**				
Ease of booking	519 (84.5)	305 (85.0)	214 (83.9)	0.92 (0.59-1.44)
Choice of appointments	486 (79.2)	280 (78.0)	206 (80.8)	1.19 (0.83-1.77)
Being able to change an appointment after booking	474 (77.2)	274 (76.3)	200 (78.4)	1.13 (0.77-1.66)
How long it takes to book appointment	424 (69.1)	235 (65.5)	189 (74.1)	1.51 (1.06-2.15)*
Waiting time for next available appointment	428 (69.7)	245 (68.2)	183 (71.8)	1.18 (0.83-1.68)
Privacy when booking appointment	410 (66.8)	230 (64.1)	180 (70.6)	1.35 (0.95-1.90)
Being able to talk with a healthcare professional when booking	345 (56.2)	195 (54.3)	150 (58.8)	1.20 (0.87-1.66)
Being able to book an appointment when the GP practice is shut	284 (46.3)	173 (48.2)	111 (43.5)	0.83 (0.60-1.15)[a]
Cost of making booking (i.e. phone credit)	166 (27.0)	94 (26.2)	72 (28.2)	1.11 (0.77-1.59)

Note. OR= odds ratio; CI= confidence interval; *p<0.05, **p<0.001, [a]30% missing data for this variable

For peer review only - http://bmjopen.bmj.com/site/about/guidelines.xhtml

Table 3:

Univariable logistic regression models of predictors of the acceptability of cervical screening invitation modalities (n=614)

	Posted letter		Text-message		Email		Mobile phone call		Landline phone call	
	%	OR (95% CI)	%	OR (95% CI)	%	OR (95% CI)	%	OR (95% CI)	%	OR (95% CI)
**All participants**	90.1	1.00	78.5	1.00	72.5	1.00	73.8	1.00	58.5	1.00
**Age group**										
25-34	92.2	1.00	86.7	1.00	80.9	1.00	82.4	1.00	65.0	1.00
35-44	89.1	0.69 (0.34-1.39)	84.2	0.78 (0.45-1.34)	78.2	0.76 (0.47-1.23)	80.8	0.85 (0.52-1.41)	67.5	1.06 (0.70-1.61)
45-54	86.9	0.56 (0.27-1.16)	78.7	0.64 (0.36-1.12)	74.1	0.71 (0.43-1.19)	69.1	0.52 (0.31-0.87)*	53.4	0.67 (0.43-1.04)
55-64	92.2	1.00 (0.41-2.43)	65.6	0.29 (0.16-0.50)***	60.0	0.33 (0.20-0.56)***	62.9	0.36 (0.21-0.61)***	60.4	0.85 (0.52-1.38)
**Social grade**										
AB	89.6	1.00	76.1	1.00	77.6	1.00	62.7	1.00	49.3	1.00
C1	85.4	0.68 (0.34-1.38)	77.1	1.05 (0.61-1.82)	73.2	0.79 (0.46-1.35)	67.5	1.24 (0.76-2.01)	51.6	1.10 (0.69-1.74)
C2	96.5	3.20 (1.12-9.14)*	78.9	1.17 (0.67-2.06)	72.5	0.76 (0.44-1.32)	82.4	2.79 (1.60-4.86)***	65.5	1.96 (1.21-3.17)**
D	93.5	1.69 (0.63-4.58)	84.9	1.77 (0.89-3.54)	77.4	0.99 (0.53-1.86)	77.4	2.04 (1.12-3.72)*	63.4	1.79 (1.04-3.07)*
E	85.2	0.67 (0.30-1.51)	77.3	1.07 (0.56-2.02)	58.0	0.40 (0.22-0.72)**	84.1	3.15 (1.61-6.15)**	68.2	2.01 (1.26-3.87)**
**Employment**										
Employed	91.1	1.00	78.8	1.00	76.0	1.00	72.4	1.00	55.1	1.00
Unemployed	86.8	0.65 (0.37-1.12)	79.7	1.05 (0.68-1.63)	67.0	0.64 (0.44-0.94)*	80.8	1.60 (1.04-2.46)*	66.5	1.62 (1.12-2.33)*
Other (studying/retired)	95.0	1.86 (0.43-8.05)	70.0	0.63 (0.61-1.29)	62.5	0.53 (0.27-1.04)	55.0	0.47 (0.24-0.90)*	55.0	0.99 (0.52-1.92)
**Ethnicity**										
Any white	91.0	1.00	77.7	1.00	71.1	1.00	73.1	1.00	57.6	1.00
All other groups	82.1	0.45 (0.23-0.90)*	85.1	1.64 (0.81-3.30)	83.6	2.07 (1.06-4.05)*	79.1	1.39 (0.75-2.58)	65.7	1.41 (0.83-2.40)
**Caring responsibilities**										
No	89.2	1.00	75.2	1.00	68.9	1.00	66.2	1.00	55.0	1.00
Yes	91.5	1.30 (0.75-2.26)	81.1	1.42 (0.95-2.11)	75.2	1.37 (0.95-1.97)	78.8	1.90 (1.31-2.75)**	61.0	1.28 (0.92-1.79)
**Screening status**										
Intender	88.6	1.00	79.6	1.00	72.2	1.00	74.1	1.00	58.0	1.00
Maintainer	91.1	1.31 (0.77-2.23)	77.7	0.89 (0.60-1.32)	72.7	1.03 (0.72-1.47)	73.5	0.97 (0.67-1.40)	58.8	1.03 (0.74-1.43)
**Practical barriers**										
0 barriers	89.6	1.00	77.2	1.00	68.9	1.00	73.6	1.00	56.0	1.00
1 barrier	93.2	1.57 (0.76-3.26)	77.9	1.04 (0.64-1.68)	72.1	1.17 (0.75-1.81)	74.7	1.06 (0.67-1.68)	57.9	1.08 (0.72-1.62)
2 barriers	90.7	1.12 (0.55-2.31)	80.0	1.18 (0.70-1.99)	74.7	1.33 (0.83-2.14)	72.7	0.96 (0.59-1.55)	60.7	1.21 (0.79-1.87)
3 or more barriers	82.7	0.55 (0.26-1.16)	80.2	1.20 (0.63-2.28)	77.8	1.58 (0.86-2.89)	74.1	1.03 (0.57-1.85)	61.7	1.27 (0.75-2.16)

Note. OR= unadjusted odds ratio; CI= confidence interval; *p<0.05, **p<0.01, ***p<0.05

For peer review only

For peer review only - http://bmjopen.bmj.com/site/about/guidelines.xhtml

Table 4

Univariable logistic regression models of predictors of phone-based booking preferences (n=614)

	Calling the GP		Calling a 24-hour automated service		Requesting a call-back	
	% likely to book by..	OR (95% CI)	% likely to book by..	OR (95% CI)	% likely to book by..	OR (95% CI)
**All participants**	89.6	1.00	51.6	1.00	57.0	1.00
**Age group**						
25-34	90.6	1.00	60.9	1.00	59.4	1.00
35-44	89.6	0.89 (0.45-1.76)	53.0	0.73 (0.48-1.09)	61.7	1.11 (0.73-1.67)
45-54	89.1	0.84 (0.41-1.73)	44.5	0.52 (0.33-0.80)**	48.2	0.64 (0.41-0.99)*
55-64	88.2	0.78 (0.36-1.68)	41.2	0.45 (0.28-0.73)**	55.9	0.87 (0.53-1.41)
**Social grade**						
AB	88.8	1.00	50.0	1.00	53.7	1.00
C1	86.0	0.77 (0.38-1.56)	49.0	0.96 (0.61-1.53)	49.0	0.83 (0.52-1.32)
C2	93.0	1.66 (0.72-3.85)	58.5	1.41 (0.87-2.26)	59.9	1.28 (0.80-2.07)
D	92.5	1.55 (0.61-3.96)	52.7	1.11 (0.66-1.89)	63.4	1.49 (0.87-2.57)
E	88.6	0.98 (0.42-2.30)	46.6	0.87 (0.51-1.50)	64.8	1.58 (0.91-2.76)
**Employment**						
Employed	89.8	1.00	52.8	1.00	55.9	1.00
Unemployed	89.6	0.98 (0.55-1.74)	50.0	0.89 (0.63-1.27)	61.5	1.26 (0.88-1.81)
Other (studying/retired)	87.5	0.80 (0.30-2.15)	47.5	0.81 (0.42-1.55)	47.5	0.72 (0.37-1.37)
**Ethnicity**						
Any white	89.8	1.00	50.3	1.00	56.3	1.00
All other groups	88.1	0.84 (0.38-1.85)	62.7	1.66 (0.97-2.80)	62.7	1.30 (0.77-2.20)
**Caring responsibilities**						
No	89.6	1.00	50.9	1.00	49.1	1.00
Yes	90.7	1.13 (0.65-1.96)	52.7	1.08 (0.77-1.50)	62.3	1.71 (1.23-2.39)**
**Screening status**						
Intender	87.8	1.00	53.7	1.00	63.1	1.00
Maintainer	90.8	1.37 (0.81-2.30)	50.1	0.87 (0.63-1.20)	52.6	0.65 (0.47-0.90)*
**Practical barriers**						
0 barriers	87.6	1.00	47.2	1.00	53.4	1.00
1 barrier	92.6	1.79 (0.89-3.57)	47.9	1.03 (0.69-1.54)	53.2	0.99 (0.66-1.48)
2 barriers	92.0	1.63 (0.79-3.37)	56.7	1.47 (0.95-2.25)	62.7	1.47 (0.95-2.27)
3 or more barriers cited	82.7	0.68 (0.33-1.39)	63.0	1.81 (1.06-3.07)*	61.7	1.57 (0.92-2.68)

Note. OR= unadjusted odds ratio; CI= confidence interval; *p<0.05, **p<0.01, ***p<0.001

For peer review only

Table 5

Univariable logistic regression models of predictors of online booking preferences (n=614)

	Booking on a website using a desktop/laptop		Booking on a website using a smartphone [a]		Downloading an app to your smartphone [a]	
	% likely to book by..	OR (95% CI)	% likely to book by..	OR (95% CI)	% likely to book by..	OR (95% CI)
**All participants**	57.8	1.00	61.5	1.00	51.8	1.00
**Age group**						
25-34	67.7	1.00	71.9	1.00	64.9	1.00
35-44	59.6	0.70 (0.46-1.07)	64.6	0.72 (0.45-1.12)	53.7	0.63 (0.41-0.97)*
45-54	54.0	0.56 (0.36-0.88)*	53.1	0.44 (0.27-0.72)**	42.5	0.40 (0.25-0.65)***
55-64	41.2	0.33 (0.20-0.55)***	40.8	0.27 (0.15-0.48)***	28.2	0.21 (0.12-0.39)***
**Social grade**						
AB	70.1	1.00	71.4	1.00	54.8	1.00
C1	56.1	0.54 (0.33-0.88)	61.0	0.63 (0.37-1.05)	51.1	0.86 (0.53-1.40)
C2	58.5	0.60 (0.36-0.99)*	59.8	0.60 (0.35-1.01)	54.9	1.01 (0.61-1.66)
D	57.0	0.56 (0.32-0.98)*	56.6	0.52 (0.29-0.93)*	48.2	0.77 (0.44-1.34)
E	42.0	0.31 (0.18-0.54)***	52.5	0.44 (0.23-0.83)*	45.9	0.70 (0.38-1.30)
**Employment**						
Employed	62.5	1.00	65.4	1.00	55.6	1.00
Unemployed	50.0	0.60 (0.41-0.86)**	56.1	0.67 (0.46-1.00)*	48.0	0.74 (0.50-1.08)
Other (studying/retired)	47.5	0.54 (0.28-1.04)	41.4	0.37 (0.17-0.81)*	24.1	0.25 (0.11-0.61)**
**Ethnicity**						
Any white	57.4	1.00	61.1	1.00	51.5	1.00
All other groups	61.2	1.17 (0.70-1.97)	65.1	1.19 (0.69-2.06)	54.0	1.11 (0.65-1.87)
**Caring responsibilities**						
No	58.1	1.00	59.0	1.00	46.4	1.00
Yes	58.4	1.01 (0.72-1.41)	63.8	1.22 (0.85-1.77)	55.4	1.43 (1.00-2.05)
**Screening status**						
Intender	57.3	1.00	62.4	1.00	56.0	1.00
Maintainer	58.2	1.04 (0.75-1.44)	61.0	0.94 (0.66-1.34)	48.9	0.75 (0.53-1.07)
**Practical barriers**						
0 barriers	47.7	1.00	50.6	1.00	40.4	1.00
1 barrier	58.4	1.54 (1.03-2.31)*	61.3	1.54 (1.00-2.40)	51.2	1.55 (1.00-2.41)*
2 barriers	64.7	2.01 (1.30-3.11)**	68.9	2.17 (1.34-3.50)**	59.8	2.20 (1.38-3.51)**
3 or more barriers	64.2	2.32 (1.35-4.01)**	73.3	2.69 (1.48-4.87)**	64.0	2.63 (1.49-4.62)**

Note. OR= unadjusted odds ratio; CI= confidence interval; *p<0.05, **p<0.01, ***p<0.001, [a] participants with no smartphone removed from analyses (*n* = 81)

For peer review only - http://bmjopen.bmj.com/site/about/guidelines.xhtml

Contributorship statement

MR (Conceptualisation; Data analysis; Project administration; Writing – original draft; Writing – review & editing)

JW (Conceptualisation; Supervision; Writing – review & editing)

LM (Conceptualisation; Data analysis; Supervision; Writing – review & editing)

All authors approved the final manuscript as submitted.

Competing interests

The authors have no competing interests to declare.

Funding

MR, JW and LM are supported by a Cancer Research UK Career Development Fellowship awarded to JW (C7492/A17219).

Data sharing statement

Data used and analysed in the study are available from the corresponding author on request (l.marlow@ucl.ac.uk).

The Corresponding Author has the right to grant on behalf of all authors and does grant on behalf of all authors, a worldwide licence to the Publishers and its licensees in perpetuity, in all forms, formats and media (whether known now or created in the future), to i) publish, reproduce, distribute, display and store the Contribution, ii) translate the Contribution into other languages, create adaptations, reprints, include within collections and create summaries, extracts and/or, abstracts of the Contribution, iii) create any other derivative work(s) based on the Contribution, iv) to exploit all subsidiary rights in the Contribution, v) the inclusion of electronic links from the Contribution to third party material where-ever it may be located; and, vi) licence any third party to do any or all of the above.

For peer review only

References

1. NHS. Cervical Screening Programme, England 2016-17, National Statistics. In: Screening & Immunisations Team ND, (ed.). 2017.
2. Landy R, Pesola F, Castanon A, et al. Impact of cervical screening on cervical cancer mortality: estimation using stage-specific results from a nested case-control study. *British journal of cancer* 2016; 115: 1140-1146. 2016/10/26. DOI: 10.1038/bjc.2016.290.
3. Waller J, Jackowska M, Marlow L, et al. Exploring age differences in reasons for nonattendance for cervical screening: a qualitative study. *BJOG : an international journal of obstetrics and gynaecology* 2012; 119: 26-32. 2011/06/15. DOI: 10.1111/j.1471-0528.2011.03030.x.
4. Hope KA, Moss E, Redman CWE, et al. Psycho-social influences upon older women's decision to attend cervical screening: A review of current evidence. *Prev Med* 2017; 101: 60-66. 2017/05/16. DOI: 10.1016/j.ypmed.2017.05.002.
5. Douglas E, Waller J, Duffy SW, et al. Socioeconomic inequalities in breast and cervical screening coverage in England: are we closing the gap? *Journal of medical screening* 2016; 23: 98-103. 2015/09/18. DOI: 10.1177/0969141315600192.
6. Spencer AM, Roberts SA, Brabin L, et al. Sociodemographic factors predicting mother's cervical screening and daughter's HPV vaccination uptake. *Journal of epidemiology and community health* 2014; 68: 571-577. 2014/02/26. DOI: 10.1136/jech-2013-202629.
7. Sutton S and Rutherford C. Sociodemographic and attitudinal correlates of cervical screening uptake in a national sample of women in Britain. *Social Science & Medicine* 2005; 61: 2460-2465. DOI: https://doi.org/10.1016/j.socscimed.2005.07.017.
8. Young B, Bedford L, Kendrick D, et al. Factors influencing the decision to attend screening for cancer in the UK: a meta-ethnography of qualitative research. *Journal of public health (Oxford, England)* 2018; 40: 315-339. 2017/05/10. DOI: 10.1093/pubmed/fdx026.
9. Marlow LAV, Ferrer RA, Chorley AJ, et al. Variation in health beliefs across different types of cervical screening non-participants. *Preventive Medicine* 2018; 111: 204-209. DOI: https://doi.org/10.1016/j.ypmed.2018.03.014.
10. Waller J, Bartoszek M, Marlow L, et al. Barriers to cervical cancer screening attendance in England: a population-based survey. *Journal of medical screening* 2009; 16: 199-204. 2010/01/08. DOI: 10.1258/jms.2009.009073.
11. Marlow LAV, Chorley AJ, Haddrell J, et al. Understanding the heterogeneity of cervical cancer screening non-participants: Data from a national sample of British women. *European journal of cancer (Oxford, England : 1990)* 2017; 80: 30-38. 2017/05/24. DOI: 10.1016/j.ejca.2017.04.017.
12. Sheeran P and Conner M. Improving the translation of intentions into health actions: The role of motivational coherence. *Health psychology : official journal of the Division of Health Psychology, American Psychological Association* 2017; 36: 1065-1073. 2017/08/22. DOI: 10.1037/hea0000553.
13. Sheeran P. Intention—Behavior Relations: A Conceptual and Empirical Review. *European review of social psychology* 2002; 12: 1-36. DOI: 10.1080/14792772143000003.
14. Weinstein ND. The precaution adoption process. *Health psychology : official journal of the Division of Health Psychology, American Psychological Association* 1988; 7: 355-386. 1988/01/01.
15. Sheeran P and Webb T. The Intention–Behavior Gap. *Social and Personality Psychology Compass* 2016; 10: 503-518. DOI: doi:10.1111/spc3.12265.
16. Everett T, Bryant A, Griffin MF, et al. Interventions targeted at women to encourage the uptake of cervical screening. *The Cochrane database of systematic reviews* 2011: CD002834. 2011/05/13. DOI: 10.1002/14651858.CD002834.pub2.
17. McDowell I, Newell C and Rosser W. Computerized reminders to encourage cervical screening in family practice. *The Journal of family practice* 1989; 28: 420-424. 1989/04/01.
18. Binstock MA, Geiger AM, Hackett JR, et al. Pap smear outreach: a randomized controlled trial in an HMO. *American journal of preventive medicine* 1997; 13: 425-426. 1998/01/07.

For peer review only - http://bmjopen.bmj.com/site/about/guidelines.xhtml

19. Duffy SW, Myles JP, Maroni R, et al. Rapid review of evaluation of interventions to improve participation in cancer screening services. *Journal of medical screening* 2017; 24: 127-145. 2016/10/19. DOI: 10.1177/0969141316664757.
20. Kitchener HC, Gittins M, Rivero-Arias O, et al. A cluster randomised trial of strategies to increase cervical screening uptake at first invitation (STRATEGIC). *Health technology assessment (Winchester, England)* 2016; 20: 1-138. 2016/09/17. DOI: 10.3310/hta20680.
21. Ipsos. Social grade: a classification tool. 2009.
22. Sheeran P and Orbell S. Using implementation intentions to increase attendance for cervical cancer screening. *Health Psychology* 2000; 19: 283.
23. Kerrison RS, Shukla H, Cunningham D, et al. Text-message reminders increase uptake of routine breast screening appointments: a randomised controlled trial in a hard-to-reach population. *British journal of cancer* 2015; 112: 1005-1010. 2015/02/11. DOI: 10.1038/bjc.2015.36.
24. Chorley AJ, Marlow LA, Forster AS, et al. Experiences of cervical screening and barriers to participation in the context of an organised programme: a systematic review and thematic synthesis. *Psycho-oncology* 2017; 26: 161-172. 2016/04/14. DOI: 10.1002/pon.4126.
25. Quinn M, Babb P, Jones J, et al. Effect of screening on incidence of and mortality from cancer of cervix in England: evaluation based on routinely collected statistics. *BMJ (Clinical research ed)* 1999; 318: 904-908. 1999/04/02.
26. ONS. Labour Force Survey. Office for National Statistics, 2011.
27. GPPS. GP Patient Survey National results and trends. 2017.
28. MORI I. The GP Patient Survey 2018. Ipsos MORI, 2018.
29. Einstein GO, McDaniel MA, Williford CL, et al. Forgetting of intentions in demanding situations is rapid. *Journal of experimental psychology Applied* 2003; 9: 147-162. 2003/10/23. DOI: 10.1037/1076-898x.9.3.147.
30. ONS. Ethnicity and National Identity in England and Wales: 2011. In: Statistics OfN, (ed.). 2011.
31. Johnson HC, Lafferty EI, Eggo RM, et al. Effect of HPV vaccination and cervical cancer screening in England by ethnicity: a modelling study. *The Lancet Public health* 2018; 3: e44-e51. 2018/01/09. DOI: 10.1016/s2468-2667(17)30238-4.
32. Howard M, Agarwal G and Lytwyn A. Accuracy of self-reports of Pap and mammography screening compared to medical record: a meta-analysis. *Cancer Causes & Control* 2008; 20: 1. journal article. DOI: 10.1007/s10552-008-9228-4.
33. Rauscher GH, Johnson TP, Cho YI, et al. Accuracy of Self-Reported Cancer-Screening Histories: A Meta-analysis. *Cancer Epidemiology Biomarkers & Prevention* 2008; 17: 748-757. DOI: 10.1158/1055-9965.epi-07-2629.

For peer review only - http://bmjopen.bmj.com/site/about/guidelines.xhtml

Online Supplement 1: Could changing invitation and booking processes help women translate their cervical screening intentions into action? A population-based survey of women's preferences in Great Britain.
(Mairead Ryan, Jo Waller and Laura Marlow)

Questionnaire

Have you ever been diagnosed with cervical cancer?
1 Yes
2 No

The next few questions in this section are about cervical screening, also known as a smear or a Pap test. The NHS Cervical Screening Programme invites women in England for a cervical screening, smear or Pap test every 3 years from age 25 to age 49 and every 5 years from age 50 to age 64. Which of these statements describes whether you have had a cervical screening? If you have had a cervical screening and can't remember when, please give your best estimate.
1 I have had a test within the last 3 years > INCLUDE (1)
2 My last test was 3 to 5 years ago > INCLUDE (2)
3 My last test was more than 5 years ago > INCLUDE (3)
4 I have never been invited to have a test > EXCLUDE
5 I have been invited but have never had a test > INCLUDE (4)
6 I have had a hysterectomy so I don't need to have tests > EXCLUDE
7 I have never heard of cervical screening > EXCLUDE

Will you go for cervical screening when next invited?
1 Definitely not > EXCLUDE
2 Probably not > EXCLUDE
3 Yes, probably > INCLUDE (a)
4 Yes, definitely > INCLUDE (a)

NB: Participants were categorised as follows based on responses to the above questions:

If answered 1 and a = maintainer

If 25-49 years and answered 2 and a = intender

If 50-64 and answered 2 and a = maintainer

If answered 3 or 4 and a = intender

On the next screen will be an invitation letter that the NHS sends to women to invite them to book a cervical screening appointment. Most women book cervical screening appointments at their GP practice. I would like you to imagine you received this letter in the post. Please read the letter and afterwards you will be asked some questions about your response to the letter.

* Picture of NHS screening letter shown to participant

For peer review only

I will now read a number of statements relating to the cervical screening letter you've just read. After each statement, please state the extent to which you agree, on a scale from 'strongly disagree 'to 'strongly agree'.

How much do you agree or disagree with this statement?
It is easy for me to find time to read a letter like this.

1 Strongly disagree
2 Disagree
3 Neither disagree or agree
4 Agree
5 Strongly agree

How much do you agree or disagree with this statement?
I might forget to book an appointment after reading this letter.

1 Strongly disagree
2 Disagree
3 Neither disagree or agree
4 Agree
5 Strongly agree

How much do you agree or disagree with this statement?
It is difficult for me to call my GP practice during their opening hours.
*GP opening hours provided if necessary: "Opening hours are generally between 8.00am to 6.30pm Monday to Friday"

1 Strongly disagree
2 Disagree
3 Neither disagree or agree
4 Agree
5 Strongly agree

How much do you agree or disagree with this statement?
I have access to a telephone/mobile with phone credit/minutes to call my GP practice.

1 Strongly disagree
2 Disagree
3 Neither disagree or agree
4 Agree
5 Strongly agree

How much do you agree or disagree with this statement?
It would be easy for me to find the phone number for my GP practice to contact them.

1 Strongly disagree
2 Disagree
3 Neither disagree or agree
4 Agree
5 Strongly agree

For peer review only

How much do you agree or disagree with this statement?
I find it takes too long to get through to a receptionist when I phone my GP practice.

1 Strongly disagree
2 Disagree
3 Neither disagree or agree
4 Agree
5 Strongly agree

We are interested in what is important to you in terms of booking a cervical screening appointment. For the following statements I read out, please state the extent to which you think each factor is important to you, on a scale from 'very unimportant' to 'very important' when booking an appointment at your GP practice.

How important is this when booking a cervical screening appointment at your GP practice?
Ease of booking

1 Very unimportant
2 Quite unimportant
3 Neither unimportant or important
4 Quite important
5 Very important

How important is this when booking a cervical screening appointment at your GP practice?
Cost of making booking (i.e. phone credit)

1 Very unimportant
2 Quite unimportant
3 Neither unimportant or important
4 Quite important
5 Very important

How important is this when booking a cervical screening appointment at your GP practice?
Choice of appointment times

1 Very unimportant
2 Quite unimportant
3 Neither unimportant or important
4 Quite important
5 Very important

How important is this when booking a cervical screening appointment at your GP practice?
Being able to change an appointment time/day after booking it

1 Very unimportant
2 Quite unimportant
3 Neither unimportant or important
4 Quite important
5 Very important

For peer review only

How important is this when booking a cervical screening appointment at your GP practice?
Privacy when booking an appointment

1 Very unimportant
2 Quite unimportant
3 Neither unimportant or important
4 Quite important
5 Very important

How important is this when booking a cervical screening appointment at your GP practice?
How long it takes to book an appointment

1 Very unimportant
2 Quite unimportant
3 Neither unimportant or important
4 Quite important
5 Very important

How important is this when booking a cervical screening appointment at your GP practice?
Being able to talk with a healthcare professional when booking (e.g. to ask questions about the screening before attending)

1 Very unimportant
2 Quite unimportant
3 Neither unimportant or important
4 Quite important
5 Very important

How important is this when booking a cervical screening appointment at your GP practice?
Time to the next available appointment (e.g. next available appointment isn't for two weeks)

1 Very unimportant
2 Quite unimportant
3 Neither unimportant or important
4 Quite important
5 Very important

How important is this when booking a cervical screening appointment at your GP practice?
Being able to book an appointment when the GP practice is shut (e.g. online booking)
*GP opening hours provided if necessary: "Opening hours are generally between 8.00am to 6.30pm Monday to Friday"

1 Very unimportant
2 Quite unimportant
3 Neither unimportant or important
4 Quite important
5 Very important

Again thinking about the letter you read which is sent in the post to invite women to book a cervical screening appointment. We are interested in different forms of communication to invite women to book a cervical screening appointment.

Please state the extent to which you think the following forms of communication are acceptable, on a scale from 'very unacceptable' to 'very acceptable'.

For peer review only

How acceptable is this form of communication when being invited to book a cervical screening appointment?
Posted letter

1 Very unacceptable
2 Quite unacceptable
3 Neither unacceptable or acceptable
4 Quite acceptable
5 Very acceptable

* If participant responded 'Quite unacceptable' or 'Very unacceptable', participant subsequently asked: Please can you tell me why you would not find receiving an invitation for a cervical screening appointment by posted letter acceptable?
How acceptable is this form of communication when being invited to book a cervical screening appointment?
Text message

1 Very unacceptable
2 Quite unacceptable
3 Neither unacceptable or acceptable
4 Quite acceptable
5 Very acceptable

* If participant responded 'Quite unacceptable' or 'Very unacceptable', participant subsequently asked: Please can you tell me why you would not find receiving an invitation for a cervical screening appointment by text message acceptable?

How acceptable is this form of communication when being invited to book a cervical screening appointment?
Email

1 Very unacceptable
2 Quite unacceptable
3 Neither unacceptable or acceptable
4 Quite acceptable
5 Very acceptable

* If participant responded 'Quite unacceptable' or 'Very unacceptable', participant subsequently asked: Please can you tell me why you would not find receiving an invitation for a cervical screening appointment by email acceptable?

For peer review only

How acceptable is this form of communication when being invited to book a cervical screening appointment?
Phone call to your mobile phone

1 Very unacceptable
2 Quite unacceptable
3 Neither unacceptable or acceptable
4 Quite acceptable
5 Very acceptable

* If participant responded 'Quite unacceptable' or 'Very unacceptable', participant subsequently asked: Please can you tell me why you would not find receiving an invitation for a cervical screening appointment by phone call to your mobile phone acceptable?

How acceptable is this form of communication when being invited to book a cervical screening appointment?
Phone call to your house landline

1 Very unacceptable
2 Quite unacceptable
3 Neither unacceptable or acceptable
4 Quite acceptable
5 Very acceptable

* If participant responded 'Quite unacceptable' or 'Very unacceptable', participant subsequently asked: Please can you tell me why you would not find receiving an invitation for a cervical screening appointment by phone call to your house landline acceptable?

Imagine now that different options were available to you to book a cervical screening appointment at your GP practice. Please state the extent to which you are likely to use each of the following methods to book an appointment.

How likely are you to use this method to book a cervical screening appointment at your GP practice?
Calling your GP practice

1 Very unlikely
2 Quite unlikely
3 Neither likely or unlikely
4 Quite likely
5 Very likely

How likely are you to use this method to book a cervical screening appointment at your GP practice?
Calling a 24-hour automated telephone appointment-booking system

1 Very unlikely
2 Quite unlikely
3 Neither likely or unlikely
4 Quite likely
5 Very likely

For peer review only

How likely are you to use this method to book a cervical screening appointment at your GP practice?
Requesting a call-back from your GP practice

1 Very unlikely
2 Quite unlikely
3 Neither likely or unlikely
4 Quite likely
5 Very likely

How likely are you to use this method to book a cervical screening appointment at your GP practice?
Booking on a website using a desktop computer/laptop

1 Very unlikely
2 Quite unlikely
3 Neither likely or unlikely
4 Quite likely
5 Very likely

How likely are you to use this method to book a cervical screening appointment at your GP practice?
Booking on a website using a smartphone

1 Very unlikely
2 Quite unlikely
3 Neither likely or unlikely
4 Quite likely
5 Very likely

How likely are you to use this method to book a cervical screening appointment at your GP practice?
Downloading an app to a smartphone to book an appointment (you could then use the app to book other appointments at your surgery)

1 Very unlikely
2 Quite unlikely
3 Neither likely or unlikely
4 Quite likely
5 Very likely

Which of the following methods have you previously used to book an appointment at your GP practice? This could be an appointment for anything, with a GP or with a nurse.
Please select all that apply.

1 Booked in person (i.e. at the reception desk)
2 Booked by phoning the GP practice
3 Booked using a 24-hour automated telephone appointment-booking system
4 Booked online on a website
5 Booked by text-message
6 Booked using a smartphone app
7 Other
8 Don't know - someone else has always booked my appointments
9 I have never booked an appointment at my GP practice

For peer review only

Do you have a mobile phone?
*Description of smartphone provided if necessary; “A ‘smart phone’ is a mobile phone that performs many of the functions of a computer, typically having a touchscreen and Internet access”

1 Yes, a smart phone
2 Yes, but it is not a smart phone
3 No, I do not have a mobile phone

For peer review only

Online Supplement 2: Could changing invitation and booking processes help women translate their cervical screening intentions into action? A population-based survey of women's preferences in Great Britain.

(Mairead Ryan, Jo Waller and Laura Marlow)

Survey inclusion flow diagram

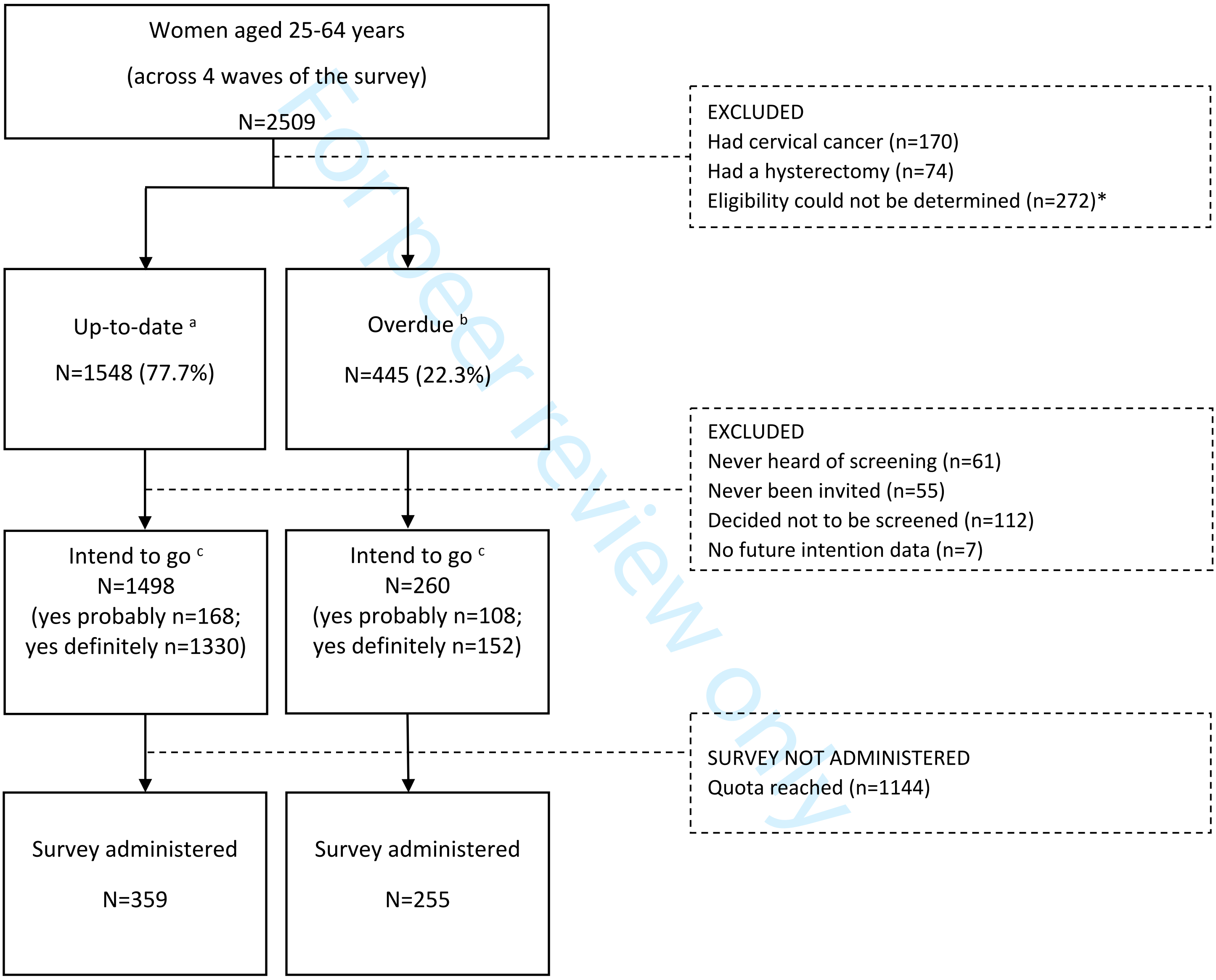

*Women who refused to answer the hysterectomy question (n=177) or screening uptake question (n=95)

[a] Up-to-date: been screened within the last 3 years if 25-64 years or the last 5 years if 50-64 years

[b] Overdue: not been screened within the last 3 years if 25-64 years or the last 5 years if 50-64 years

[c] Responded that they would 'probably' or 'definitely' attend screening when next invited

For peer review only

Online Supplement 3: Could changing invitation and booking processes help women translate their cervical screening intentions into action? A population-based survey of women's preferences in Great Britain.

(Mairead Ryan, Jo Waller and Laura Marlow)

Table 1:

Multivariable logistic regression models of predictors of the acceptability of cervical screening invitation modalities (n=614)

	**Posted letter**	**Text-message**	**Email**	**Mobile phone call**	**Landline phone call**
	OR (95% CI)	OR (95% CI)	OR (95% CI)	OR (95% CI)	OR (95% CI)
**Age group**					
25-34	1.00	1.00	1.00	1.00	1.00
35-44	0.63 (0.30-1.34)	0.71 (0.40-1.25)	0.64 (0.38-1.05)	0.78 (0.46-1.32)	1.06 (0.68-1.63)
45-54	0.49 (0.22-1.06)	0.63 (0.35-1.12)	0.71 (0.42-1.20)	0.50 (0.30-0.85)*	0.70 (0.44-1.10)
55-64	1.03 (0.36-2.94)	0.29 (0.15-0.53)***	0.35 (0.19-0.63)***	0.47 (0.26-0.86)*	0.99 (0.58-1.70)
**Social grade**					
AB	1.00	1.00	1.00	1.00	1.00
C1	0.78 (0.36-1.66)	1.03 (0.58-1.82)	0.75 (0.43-1.32)	1.17 (0.71-1.94)	1.04 (0.65-1.67)
C2	3.47 (1.18-10.27)*	1.04 (0.58-1.87)	0.68 (0.39-1.21)	2.58 (1.45-4.58)**	1.82 (1.11-2.99)
D	1.75 (0.63-4.92)	1.60 (0.78-3.28)	0.90 (0.47-1.73)	1.82 (0.98-3.39)	1.67 (0.96-2.90)
E	0.88 (0.31-2.52)	0.92 (0.42-2.01)	0.38 (0.19-0.78)**	2.98 (1.35-6.58)**	1.76 (0.90-3.43)
**Employment**					
Employed	1.00	1.00	1.00	1.00	1.00
Unemployed	0.63 (0.30-1.33)	1.12 (0.65-1.94)	0.89 (0.54-1.47)	1.06 (0.63-1.79)	1.31 (0.83-2.06)
Other (studying/retired)†	-	-	-	-	-
**Ethnicity**					
White	1.00	1.00	1.00	1.00	1.00
All other groups	0.45 (0.21-0.97)*	1.60 (0.75-3.39)	2.22 (1.08-4.57)*	1.26 (0.65-2.45)	1.38 (0.79-2.42)
**Caring responsibilities**					
No	1.00	1.00	1.00	1.00	1.00
Yes	1.79 (0.96-3.23)	1.09 (0.70-1.69)	1.19 (0.79-1.80)	1.60 (1.06-2.41)*	1.20 (0.83-1.73)

Note. OR= adjusted odds ratio; CI= confidence interval; *p<0.05, **p<0.01, ***p<0.001; 'screening status' and 'practical barriers' variables not included because not significant in univariable analyses; †category not included due to insufficient cases

For peer review only

Table 2

Multivariable logistic regression models of predictors of phone-based booking preferences (n=614)

	**Calling the GP**	**Calling a 24-hour automated service**	**Requesting a call-back**
	OR (95% CI)	OR (95% CI)	OR (95% CI)
**Age group**			
25-34	1.00	1.00	1.00
35-44	0.78 (0.38-1.60)	0.68 (0.45-1.04)	0.96 (0.62-1.47)
45-54	0.70 (0.32-1.50)	0.50 (0.32-0.79)*	0.65 (0.41-1.03)
55-64	0.76 (0.31-1.83)	0.45 (0.27-0.76)**	1.19 (0.70-2.01)
**Caring responsibilities**			
No	1.00	1.00	1.00
Yes	1.11 (0.61-2.01)	0.95 (0.67-1.37)	1.82 (1.26-2.62)**
**Screening status**			
Intender	1.00	1.00	1.00
Maintainer	1.55 (0.89-2.68)	0.97 (0.70-1.36)	0.68 (0.48-0.95)*
**Practical barriers**			
0 barriers	1.00	1.00	1.00
1 barrier	1.48 (0.71-3.06)	0.90 (0.59-1.36)	0.81 (0.53-1.23)
2 barriers	1.39 (0.65-2.97)	1.26 (0.81-1.96)	1.26 (0.80-1.97)
3 or more barriers cited	0.63 (0.29-1.37)	1.63 (0.94-2.82)	1.35 (0.77-2.36)

Note. OR= adjusted odds ratio; CI= confidence interval; *p<0.05, **p<0.01, ***p<0.001; 'social grade', 'employment' and 'ethnicity' not included because not significant in univariable analyses

For peer review only

Table 3

Multivariable logistic regression models of predictors of online booking preferences (n=614)

	**Booking on a website using a desktop/laptop**	**Booking on a website using a smartphone [a]**	**Downloading an app to your smartphone [a]**
	OR (95% CI)	OR (95% CI)	OR (95% CI)
**Age group**			
25-34	1.00	1.00	1.00
35-44	0.64 (0.42-1.00)*	0.63 (0.39-1.01)	0.56 (0.36-0.88)*
45-54	0.58 (0.36-0.93)*	0.42 (0.25-0.71)**	0.39 (0.24-0.65)***
55-64	0.34 (0.20-0.58)***	0.28 (0.15-0.52)***	0.25 (0.13-0.47)***
**Social grade**			
AB	1.00	1.00	1.00
C1	0.52 (0.31-0.86)*	0.57 (0.33-0.98)*	0.81 (0.48-1.35)
C2	0.55 (0.33-0.93)*	0.49 (0.28-0.86)*	0.87 (0.51-1.49)
D	0.50 (0.78-0.89)*	0.42 (0.23-0.79)**	0.65 (0.36-1.18)
E	0.35 (0.18-0.69)**	0.47 (0.21-1.03)	0.78 (0.36-1.69)
**Employment**			
Employed	1.00	1.00	1.00
Unemployed	0.80 (0.51-1.24)	0.75 (0.46-1.24)	0.73 (0.44-1.18)
Other (studying/retired)	0.82 (0.39-1.73)	0.50 (0.21-1.16)	0.34 (0.13-0.87)*
**Practical barriers**			
0 barriers	1.00	1.00	1.00
1 barrier	1.46 (0.96-2.23)	1.33 (0.84-2.12)	1.33 (0.84-2.12)
2 barriers	1.73 (1.10-2.73)*	1.76 (1.07-2.91)*	1.78 (1.09-2.90)*
3 or more barriers	2.16 (1.22-3.82)**	2.63 (0.40-4.92)**	2.45 (1.35-4.44)**

Note. OR= adjusted odds ratio; CI= confidence interval; *p<0.05, **p<0.01, ***p<0.001; [a] participants with no smartphone removed from analyses (*n* = 81); 'ethnicity', 'caring responsibilities' and 'screening status' not included because not significant in univariable analyses

For peer review only

Online Supplement 4: Could changing invitation and booking processes help women translate their cervical screening intentions into action? A population-based survey of women's preferences in Great Britain.

(Mairead Ryan, Jo Waller and Laura Marlow)

Open responses provided for citing invitation method as unacceptable

Invitation mode	Unacceptable (n)	Reasons for being unacceptable
Posted letter	12	Don't open post/might miss the letter/no time to read letter (n=4)<br>Receive letter too late (n=2)<br>Letter could be lost in the post (n=2)<br>Other (n=4)<br>• Would forget (n=1)<br>• Environmental concerns (n=1)<br>• Waste of time (n=1)<br>• No reason provided (n=1)
Text-message	67	Privacy concerns (n=21)<br>Easy to miss it/may not read message (n=9)<br>Reason not provided (i.e. N/A) (n=9)<br>Doesn't have or use mobile (n=7)<br>Impersonal (n=6)<br>Could change number (n=4)<br>Prefer a letter/phone call (n=4)<br>Not reliable source/unprofessional (n=3)<br>Would forget/not act on it (n=2)<br>Other (n=2)<br>• Don't know (n=1)<br>• They can text me but I don't want to text them (n=1)
Email	94	Would be lost in other emails/would not be seen (n=38)<br>No email/doesn't use email/no internet/no computer (n=17)<br>Privacy concerns (n=12)<br>Reason not provided (i.e. N/A) (n=12)<br>Prefer phone or letter (n=5)<br>Would forget/not act on it (n=2)<br>Impersonal/rude (n=2)<br>Other (n=6)<br>• Not timely (n=1)<br>• Intrusive (n=1)<br>• Not normal (n=1)<br>• No reason (n=1)<br>• Not keen (n=1)<br>• Doesn't trust source (n=1)
Mobile	90	Would not be able to pick up/would miss call (n=33)

For peer review only

phone call		Privacy concerns (n=22)<br>Would prefer in writing/a letter (n=10)<br>Reason not provided (i.e. N/A) (n=8)<br>Would not know number – so would not answer call (n=5)<br>No mobile (n=2)<br>Would forget (n=2)<br>Too many phone calls (n=2)<br>Other (n=6)<br>• Don’t like idea (n=1)<br>• Talking takes too much time (n=1)<br>• Need time to think (n=1)<br>• Impersonal (n=1)<br>• People change phone number (n=1)<br>• Don’t like calls (n=1)
Landline phone call	129	No landline (n=39)<br>Would miss call/out of the house during the day (n=31)<br>Privacy concerns (n=24)<br>No reason provided (i.e. N/A) (n=12)<br>Feels intrusive (n=5)<br>Prefer in writing/letter (n=5)<br>Don’t want phone call (n=4)<br>Not reliable source (n=3)<br>Other (n=6)<br>• Impersonal (n=1)<br>• “Better with working” (n=1)<br>• Unnecessary (n=1)<br>• Unknown number (n=1)<br>• Want time to think (n=1)<br>• Doesn’t matter either way (n=1)

For peer review only

STROBE 2007 (v4) Statement—Checklist of items that should be included in reports of *cross-sectional studies*

Section/Topic	Item #	Recommendation	Reported on page #
**Title and abstract**	1	(*a*) Indicate the study's design with a commonly used term in the title or the abstract	2
		(*b*) Provide in the abstract an informative and balanced summary of what was done and what was found	2
**Introduction**			
Background/rationale	2	Explain the scientific background and rationale for the investigation being reported	3-4
Objectives	3	State specific objectives, including any prespecified hypotheses	4
**Methods**			
Study design	4	Present key elements of study design early in the paper	4
Setting	5	Describe the setting, locations, and relevant dates, including periods of recruitment, exposure, follow-up, and data collection	4
Participants	6	(*a*) Give the eligibility criteria, and the sources and methods of selection of participants	4-5
Variables	7	Clearly define all outcomes, exposures, predictors, potential confounders, and effect modifiers. Give diagnostic criteria, if applicable	5-6
Data sources/ measurement	8*	For each variable of interest, give sources of data and details of methods of assessment (measurement). Describe comparability of assessment methods if there is more than one group	5-6
Bias	9	Describe any efforts to address potential sources of bias	4
Study size	10	Explain how the study size was arrived at	5
Quantitative variables	11	Explain how quantitative variables were handled in the analyses. If applicable, describe which groupings were chosen and why	6
Statistical methods	12	(*a*) Describe all statistical methods, including those used to control for confounding	6
		(*b*) Describe any methods used to examine subgroups and interactions	6
		(*c*) Explain how missing data were addressed	
		(*d*) If applicable, describe analytical methods taking account of sampling strategy	
		(*e*) Describe any sensitivity analyses	
**Results**			

For peer review only

Participants	13*	(a) Report numbers of individuals at each stage of study—eg numbers potentially eligible, examined for eligibility, confirmed eligible, included in the study, completing follow-up, and analysed	6
		(b) Give reasons for non-participation at each stage	n/a
		(c) Consider use of a flow diagram	
Descriptive data	14*	(a) Give characteristics of study participants (eg demographic, clinical, social) and information on exposures and potential confounders	6-7
		(b) Indicate number of participants with missing data for each variable of interest	
Outcome data	15*	Report numbers of outcome events or summary measures	
Main results	16	(*a*) Give unadjusted estimates and, if applicable, confounder-adjusted estimates and their precision (eg, 95% confidence interval). Make clear which confounders were adjusted for and why they were included	7-8, 12-15
		(*b*) Report category boundaries when continuous variables were categorized	5-6
		(*c*) If relevant, consider translating estimates of relative risk into absolute risk for a meaningful time period	n/a
Other analyses	17	Report other analyses done—eg analyses of subgroups and interactions, and sensitivity analyses	
**Discussion**			
Key results	18	Summarise key results with reference to study objectives	8-10
Limitations	19	Discuss limitations of the study, taking into account sources of potential bias or imprecision. Discuss both direction and magnitude of any potential bias	9-10
Interpretation	20	Give a cautious overall interpretation of results considering objectives, limitations, multiplicity of analyses, results from similar studies, and other relevant evidence	10
Generalisability	21	Discuss the generalisability (external validity) of the study results	9-10
**Other information**			
Funding	22	Give the source of funding and the role of the funders for the present study and, if applicable, for the original study on which the present article is based	1

*Give information separately for cases and controls in case-control studies and, if applicable, for exposed and unexposed groups in cohort and cross-sectional studies.

Note: An Explanation and Elaboration article discusses each checklist item and gives methodological background and published examples of transparent reporting. The STROBE checklist is best used in conjunction with this article (freely available on the Web sites of PLoS Medicine at http://www.plosmedicine.org/, Annals of Internal Medicine at http://www.annals.org/, and Epidemiology at http://www.epidem.com/). Information on the STROBE Initiative is available at www.strobe-statement.org.

For peer review only

BMJ Open

Could changing invitation and booking processes help women translate their cervical screening intentions into action? A population-based survey of women's preferences in Great Britain.

Journal:	*BMJ Open*
Manuscript ID	bmjopen-2018-028134.R2
Article Type:	Research
Date Submitted by the Author:	28-May-2019
Complete List of Authors:	Ryan, Mairead; University College London Institute of Epidemiology and Health Care, Department of Behavioural Science and Health;<br>Waller, Jo; UCL, Epidemiology and Public Health<br>Marlow, Laura; University College London
**Primary Subject Heading**:	Epidemiology
Secondary Subject Heading:	Epidemiology, Communication
Keywords:	cervical cancer screening, non-participants, interventions, age, screening status, uptake

SCHOLARONE™
Manuscripts

Could changing invitation and booking processes help women translate their cervical screening intentions into action? A population-based survey of women's preferences in Great Britain.

Mairead Ryan, MSc
Jo Waller, PhD
Laura A.V Marlow, PhD

Cancer Communication and Screening Group, Research Department of Behavioural Science and Health, University College London, Gower Street, London, UK

Corresponding author contact details: Laura Marlow, l.marlow@ucl.ac.uk, 020 7679 1798.

MR, JW and LM are supported by a Cancer Research UK Career Development Fellowship awarded to JW (C7492/A17219).

For peer review only

Abstract

Objectives: Many women who do not attend screening intend to go, but do not get around to booking an appointment. Qualitative work suggests these 'intenders' face more practical barriers to screening than women who are up-to-date ('maintainers'). This study explored practical barriers to booking a screening appointment and preferences for alternative invitation and booking methods that might overcome these barriers.

Design: A cross-sectional survey was employed.

Setting: Great Britain

Participants: Women aged 25 to 64, living in Great Britain who intended to be screened but were overdue ('intenders', n=255) and women who were up-to-date with screening ('maintainers', n=359)

Results: 'Intenders' reported slightly more barriers than 'maintainers' overall (mean = 1.36 vs 1.06, t=3.03, p <0.01) and were more likely to think they might forget to book an appointment (Odds ratio=2.87, 95% confidence interval: 2.01-4.09). Over half of women said they would book on a website using a smartphone (62%),a computer (58%) or via an app (52%). Older women and women from lower social grades were less likely to say they would use online booking methods (all ps<.05). Women who reported two or more barriers were more likely to say they would use online booking than women who reported none (ps<.01).

Conclusions: Women who are overdue for screening face practical barriers to booking appointments. Future interventions may assess the efficacy of changing the architecture of the invitation and booking system. This may help women overcome logistical barriers to participation and increase coverage for cervical screening.

Strengths and limitations of this study

- This was the first study to break down the invitation and booking process into its component parts, identifying barriers at each stage of the process and alternative booking options which may help women to overcome these barriers

For peer review only - http://bmjopen.bmj.com/site/about/guidelines.xhtml

- Women were purposely recruited to be up-to-date and overdue, however response rate was not recorded.

- The practical barriers cited in this study relate to the booking process and are not exhaustive of all practical barriers to cervical screening. They may not reflect booking processes in other countries.

Introduction

Cervical screening programmes are designed to reduce the incidence and mortality rate of cervical cancer.[1] In Great Britain all eligible women aged 25 to 64 registered with a GP are invited to be screened for the presence of abnormal cell changes in the cervix, which could, if undetected and untreated, develop into cervical cancer. The efficacy of the programme has been widely acknowledged,[2] however the success of any screening programme is dependent on good coverage. In 2017, coverage (i.e. the percentage of eligible women recorded as adequately screened) was 72%, well below the national target of 80% and in keeping with a trend of decreasing screening coverage.

Reasons for screening non-attendance are complex and differ depending on socio-demographic factors such as age, socio-economic status and marital status.[3-6] Emotional barriers including embarrassment, fear of pain and negative experiences are often reported, particularly in qualitative studies.[7-9] While these barriers undoubtedly need to be addressed, practical barriers have been found to be more predictive of screening status than emotional barriers.[10] Recent research showed that over half of women overdue for cervical screening have positive intentions to attend.[11] While this is encouraging, intentions are frequently not translated into action.[12, 13]

Weinstein used a 'messy desk' analogy to help explain the problem of translating intentions into action.[14] He proposed that we do not carry out errands in a logical sequence, but rather in a haphazard manner, acting on 'to-do' list items when we feel pressure, when items need to be actioned quickly, when prompted or because of personal preference. More recently, Sheeran and Webb identified three key problems (or 'TRIALS') people might encounter when trying to realise their intentions; i) they fail to get started (e.g. forget to act or miss an opportunity to act), ii) they fail to keep the goal on track (fail to monitor the goal, face competing thoughts or distractions) and iii) they fail to close (don't quite meet the goal).[15]

Women receive a posted letter inviting them to book a screening appointment. The letter states the recipient "can make an appointment for cervical screening by phoning *(their)* GP surgery". GP surgery hours generally coincide with 'normal' working hours, presenting several practical barriers for women who are in full-time employment or who have caring responsibilities, both in terms of

For peer review only - http://bmjopen.bmj.com/site/about/guidelines.xhtml

phoning and attending a GP surgery. Previous research has identified that many women find the booking process arduous and inflexible.[3]

Few studies have assessed alternative methods of inviting women for cervical screening.[16] The most recent Cochrane review of interventions to improve uptake [16] reported two studies from the 1980s and 90s, which found that participants who received a telephone invitation were significantly more likely to attend than those who received a letter.[17, 18] Studies which have examined the utility of more recent technological developments to invite women are lacking.[19] There is also a paucity of literature concerning alternative booking methods for cervical screening, most likely due to limited booking options being available until recently. One trial investigated the efficacy of online booking among first time invitees.[20] The intervention group booked slightly more appointments within three months (2.18% higher than the control group) however, this was not statistically significant.[20] The authors noted that the way the online booking system was offered could account for the lack of support (in a letter participants were asked to visit a website to book at one of three sexual health clinics). Hence, other forms of online booking may be desirable to women.

New technologies offer opportunities for editing the architecture of the invitation and booking system in ways that may help to overcome some of the challenges women face between forming a positive intention and translating this into behaviour, as highlighted in the TRIALS model. For example, online booking methods may reduce the likelihood that women would fail to get started, given that opportunities to act (i.e. book an appointment) are not limited to GP practice opening hours. The present study explored practical barriers to booking an appointment among two groups: women who are up-to-date with screening ('maintainers') and women who intend to be screened but are currently overdue ('intenders'). Our aim was to examine between-group differences which may account for this intention-behaviour gap among 'intenders'. We also assessed invitation and booking preferences and explored whether these might help to overcome practical barriers.

Methods

Participants

Participants were recruited by Kantar TNS UK as part of their omnibus survey. The TNS omnibus survey recruits a new sample of 2000-4000 men and women living in Great Britain on a weekly basis and asks questions on a range of topics commissioned by external companies. Recruitment uses random location sampling to identify areas for sampling participants using the 2011 Census and the Postcode Address File. Recruiters visit homes in the identified areas and knock on doors asking those who answer to participate. All interviews are conducted in English. Quotas are set at each location for age, gender, working status, and presence of children in the household.

For peer review only - http://bmjopen.bmj.com/site/about/guidelines.xhtml

Women who were eligible for cervical screening and had not previously been diagnosed with cervical cancer, were asked to report their past attendance at cervical screening and future intention to attend (see Online Supplement 1). Responses to these questions were used to classify women as ‘intenders’ (intended to be screened but were currently overdue), ‘maintainers’ (up-to-date with screening and intending to go in the future) or ‘other’ (never heard of screening, never been invited, decided not to be screened). A sample of 600 women was expected to allow us to establish a significant difference of 5% between preferred booking options in the two groups of attenders within +/- 8% with 95% confidence.

Procedure

Ethical approval was granted by University College London Research Ethics Committee (reference: 10353/003). Data were collected between April and May 2018. Face-to-face computer-assisted personal interviews were used to collect data. Kantar TNS provided anonymised data to UCL for analysis.

Measures

Invitation preferences: Participants were asked whether several different modes of communication were acceptable to them as a means of being invited to book a cervical screening appointment (see Online Supplement 1). Participants’ responses were recoded as ‘acceptable’ (if they responded quite acceptable/very acceptable) or ‘unacceptable/ambivalent’ (if they responded quite unacceptable/very unacceptable/neither unacceptable nor acceptable). Participants who responded quite/very unacceptable were asked to explain why (open response).

Practical barriers to booking an appointment: Participants were asked to respond to a list of barriers, which were based on the key problems outlined in the TRIALS model.[15] Statements addressing the key problem of ‘failing to get started’ included ‘It is easy for me to find time to read a letter like this’ and ‘I might forget to book an appointment after reading this letter’. Statements addressing ‘failing to keep the goal on track’ included ‘It is difficult for me to call my GP practice during their opening hours’ and ‘I find it difficult to get through to a receptionist when I phone my GP practice’. Women were then asked to state which booking attributes were important to them, the aim of which was to address factors that might influence ‘failure to close’ (i.e. being able to book the appointment).

Booking preferences: Participants were asked to indicate how likely they would be to use different booking methods. The feasibility of these methods were informally discussed with stakeholders from the NHS national screening programme and with representatives from a technology company, who develop methods of improving access to healthcare. Participants’ responses were recoded as ‘likely to use’ a method (if they responded quite likely/very likely) or ‘not likely to use/ambivalent’ (if they

For peer review only - http://bmjopen.bmj.com/site/about/guidelines.xhtml

responded quite unlikely/very unlikely/neither unlikely nor likely). Participants were also asked to indicate which booking methods they had used in the past for any GP appointment.

Socio-demographic and background factors: Data regarding age, ethnicity, education level, employment status, marital status, social grade, child/carer responsibilities and smartphone ownership were also collected. Social grade is determined by the occupation of the Chief Income Earner in the household and is classified as follows: AB managerial/professional; C1 supervisory; C2 skilled manual; D semi-skilled/unskilled manual; E casual workers/unemployed.[21]

Patient and Public Involvement Statement

The study was supported by a PPI group who provided input into the contents of the survey. A group of 10 screening-eligible women were invited to guide and refine the survey questions. Women who were both up-to-date and overdue were represented in the group. The group helped to establish the perceived difficulty of the questions (e.g. unknown terms, ambiguous concepts, long and overly complex questions) and omissions from the survey. The questions and response options were tailored based on feedback provided by this PPI group.

Analyses

All analyses were conducted using IBM SPSS version 22. Chi-squared analyses were conducted to test for significant differences in participant demographics between 'Intenders' and 'Maintainers'. Descriptive statistics were conducted to assess booking history and smartphone/mobile phone ownership across all participants. For each of the six practical barrier statements, any positively-framed items were reverse-scored so that a higher score was indicative of a barrier for all items. Total practical barrier scores were created by allocating a score of 1 for each barrier statement that a participant 'agreed' or 'strongly agreed' with and adding these together (possible range 0-6). Independent samples t-tests were conducted to assess differences in the mean barriers scores between 'intenders' and 'maintainers'. A series of binary logistic regressions were then conducted to assess the associations between endorsing each barrier/booking attribute and the unadjusted odds for being an 'intender' (versus a 'maintainer'). A series of univariable logistic regressions were conducted to explore whether socio-demographic factors, screening status and number of practical barriers reported were associated with invitation (acceptable v unacceptable/ambivalent) and booking preferences (likely to use v unlikely to use/ambivalent). Participants responding don't know or not applicable were excluded. Multivariable logistic regressions are presented as supplementary material.

Results

For peer review only - http://bmjopen.bmj.com/site/about/guidelines.xhtml

Sample characteristics

2509 eligible respondents (i.e. women aged 25-64 years) completed the Kantar TNS survey. After exclusions, 1548 (78%) were up-to-date and 445 (22%) were overdue for screening. Our questions on invitation and booking preferences for cervical screening were asked to all women who were classified as 'intenders' and women who were classified as 'maintainers' in week 1. See Online Supplement 2 for survey inclusion flow diagram.

Sample characteristics for participants classified as 'intenders' (n=255) and 'maintainers' (n=359) are presented in Table 1. Mean age was 41.69 years (SD=10.84, range: 25-64 years), the majority self-identified as White (89%), were employed (64%), married or co-habiting (67%) and had regular caring responsibilities (i.e. for children/parents; 63%). 'Intenders' (mean=39.41; SD=9.94) were significantly younger than 'maintainers' (mean=43.31; SD=11.16); $t(612)=4.47$, $p<.001$.

The majority of women had previously booked by phoning the practice (89%), over one-third had booked in person (39%) and 14% had booked on a website. 'Maintainers' were significantly more likely to have previously booked on a website than 'intenders' (see Table 1). The majority of participants had a smartphone (87%), fewer women had a mobile phone which was not a smartphone (11%) and a small minority had no mobile phone (2%).

Practical barriers to appointment booking and desired attributes

Over two-thirds of women reported one or more barriers to booking (69%); mean number of reported barriers was 1.21 (SD=1.06). 'Intenders' (mean=1.36; SD=1.06) reported slightly more barriers than 'maintainers' overall (mean=1.10; SD=1.04; $t(612)=3.03$, $p<0.01$). The most commonly endorsed barrier was 'I find it difficult to get through to a receptionist when I phone my GP practice' (50% of participants 'strongly agreed' or 'agreed'), followed by 'It is difficult for me to call my GP practice during their opening hours' (31%) and 'I might forget to book an appointment after reading this letter' (31%). Practical barriers to appointment booking and booking characteristics considered to be important are outlined in Table 2. The 'intenders' group were significantly more likely to endorse the statement 'I might forget to book an appointment after reading this letter' than 'maintainers'. 'Intenders' were also more likely to state 'How long it takes to book the appointment' was important to them than 'maintainers'.

Invitation preferences

Posted letters emerged as the most acceptable invitation mode followed by text-messages (see Table 3). Socio-demographic predictors of the acceptability of each modality are shown in Table 3. Text-message, email and mobile call invitations were less acceptable to women aged 55-64; these associations remained significant in multivariable analyses (see Online Supplement 3). Mobile and

For peer review only - http://bmjopen.bmj.com/site/about/guidelines.xhtml

landline call invites were more acceptable to women from lower socio-economic backgrounds and this remained significant in multivariable analyses for mobile invites. Reasons for considering invitation modes as unacceptable are provided in Online Supplement 4; fears about missing a phone call/email or text and privacy concerns were commonly cited. Many participants also reported they had no landline phone.

Phone-based booking preferences

Most women said they were likely to book by phoning their GP practice (90%; see Table 4). Older women were significantly less likely to say they would call a 24-hour automated service than women aged 25-34 (44% vs 63%). Women with caring responsibilities were more likely to say they would request a call-back compared to women with no caring responsibilities (63% vs 51%). 'Maintainers' were less likely to say they would request a call-back than 'intenders' (54% vs 66%). These associations remained significant in multivariable analyses. Women who cited three or more barriers were more likely to say they would call a 24-hour automated service but this association was not significant in multivariable analyses.

Online booking preferences

Booking on a website using a smartphone (59%) was the preferred online booking method (see Table 5). Older women (55-64 years) were less likely to say they would book online than younger women (25-34 years). Women in lower social grades were less likely than women in the highest grade to state they would book on a website, either using a desktop or smartphone. Participants who were studying or retired were less likely than those employed to say they would book online (either on a website using a smartphone: 41% vs 64%, or through an app: 22% vs 54%). Women who reported two or more barriers were more likely to report that they would use all online booking methods compared to women who reported no barriers (see Table 5). Age, social grade, employment status and number of barriers remained significant in multivariable analyses.

Discussion

This study examined women's practical barriers to booking a cervical screening appointment and assessed whether invitation and booking preferences are associated with reported barriers, socio-demographic factors and screening status. Approximately one-third of all women reported that it is difficult to phone their GP practice within opening hours and half reported that it is difficult to get through to a receptionist. Although the survey found that 'intenders' experience slightly more practical barriers to screening than 'maintainers', endorsement of barriers across the sample suggests that both groups need more support in booking an appointment.

For peer review only - http://bmjopen.bmj.com/site/about/guidelines.xhtml

'Intenders' were more likely to report that they would forget to book an appointment after reading the screening letter than 'maintainers'. This key problem relates to a 'failure to get started', which is a first barrier people face between forming an intention and translating this into behaviour.[15] Written reminders are an integral part of the screening programme and there is good evidence to show these improve uptake,[16] but in their current format these reminders do not seem to help all women to remember to book their appointment. Future research might explore methods of increasing the salience of cervical screening among invitees (e.g. employing implementation intentions).[22] The use of text-message reminders has shown promise in other screening contexts.[23] 'Intenders' were also more likely to say that the length of time needed to book an appointment was important to them. Since all women eligible for cervical screening fall within the working age population, and GP opening hours generally overlap with working hours, it is likely this cohort face competing obligations,[24] and, as a result 'fail to keep their goal on track'.[15] The rate of female employment (16 to 64 years) has increased from 62.2% in 1994, when coverage was high (85%; five yearly coverage for women aged 20 to 64)[25] to 70.5% in 2017.[26] Alternative booking methods may provide more flexibility.

Women who reported more barriers showed greater interest in using alternative booking methods. Specifically, participants who reported two or more barriers were more likely to say that they would book on a website or through an app. This is perhaps not surprising since these methods overcome the most common practical barriers highlighted by participants, including, difficulty getting through to a receptionist and difficulty calling the practice during opening hours; hence they 'fail to close'. Nevertheless, while 24-hour automated services offers these same advantages, consistent with previous national surveys,[27] fewer women reported that they would use this booking option. Online booking services are already set up in the majority of GP practices across England for GP appointments, however a national survey found that over 40% of patients are unaware if there are online booking services at their GP practice.[28] Hence, signposting online booking services, if available for nurse appointments, to groups of the screening-eligible population (i.e. younger women who are more likely to be 'intenders') may be an effective means of increasing uptake. This survey suggests that there are likely to be age and socio-economic inequalities in the use of online bookings. For example, women aged 45-54 years and women age 55-64 showed less interest in using online booking methods. Thus, ensuring that traditional telephone booking options remains available is important.

For peer review only - http://bmjopen.bmj.com/site/about/guidelines.xhtml

Previous research has found that it is very difficult for individuals to maintain intentions after even very brief periods of time (less than one minute), especially in circumstances where there are competing tasks.[29] Unlike posted letters, which may not be read until the end of the day, text-messages can be delivered at a time when GP practices are open, so women can act immediately on their intentions to book an appointment. Given that text-message invites were considered acceptable to the majority of women across all socio-demographic backgrounds, and have previously been found to be effective in increasing uptake for other national screening programmes,[23] the use of text-message invitations may be a worthwhile intervention to explore. Text-messages within the cervical screening programme have, thus far, been introduced as a booking reminder, rather than as a stand-alone invitation, which the current study did not specify. Some participants shared concerns that they may miss the message; outlining that text-messages would be used as a supplemental invitation may have further increased acceptability within the sample. Further research is needed to explore methods of overcoming privacy concerns associated with text-messages, which some of the participants raised.

This study had some limitations. We were unable to collect data on women who elected not to participate in the study. Hence the response rate and differences between respondents and non-respondents could not be determined. Women in the survey tended to be slightly less deprived and were less likely to be from ethnic minority backgrounds than the population represented in the most recent Census.[30] This suggests there was a slight bias in participation. This survey was also conducted in English and therefore non-English speakers were not represented. Given ethnic disparities in screening attendance in England,[31] more work is needed to explore methods of overcoming practical barriers to screening for ethnic minority women.

Participation in screening was self-reported. Previous research has found that women tend to over-report their participation in cervical screening programmes,[32, 33] thus some of the women classified as 'maintainers' may actually be overdue for screening. Furthermore, although this study explored practical barriers to appointment-booking based on the TRIALS model,[15] several other practical barriers were not assessed. For example, previous research has found that 'intenders' are more likely to have children under the age of five;[11] childcare may be an additional practical barrier to screening. Thus the barriers cited in this study are not exhaustive of all practical barriers to screening for women. In addition, the study was designed to reflect the current booking process for cervical screening in Great Britain. While there may be parallels with other countries that have call-recall programs with paper-based invitations and self-booked appointments in primary care, the findings

For peer review only - http://bmjopen.bmj.com/site/about/guidelines.xhtml

may not be generalisable to screening programmes in other countries, where the invitation and booking approach differs.

Nevertheless, this was the first study to assess preferences for booking a screening appointment in Great Britain, an important first step in the development of trialling and implementing any of these changes. The invitation and booking process was broken down to identify barriers at each stage and associated preferences which may help women to overcome such barriers. The lack of differences by screening status suggests that changing the architecture should not deter 'maintainers' from participation. Future interventions may assess the efficacy of i) signposting invitees to online booking services, ii) text-messages which are delivered during GP opening hours and iii) sending reminders to reduce the likelihood of forgetting to book an appointment. Implementation research will further determine how best to introduce such changes to the screening infrastructure.

For peer review only

For peer review only - http://bmjopen.bmj.com/site/about/guidelines.xhtml

Table 1:

Sample Characteristics (n=614)

	**Overall** (n=614) N (%)	**Maintainers** (n=359) N (%)	**Intenders** (n=255) N (%)	**Difference between maintainers and intenders** Chi Square (df), P-value
**Age (years)**				14.16 (3), <.001
25-34	192 (31.3)	103 (28.7)	89 (34.9)	
35-44	183 (29.8)	95 (26.5)	88 (34.5)	
45-54	137 (22.3)	88 (24.5)	49 (19.2)	
55-64	102 (16.6)	73 (20.3)	29 (11.4)	
**Ethnicity**				0.10 (1), 0.76
Any white	547 (89.1)	321 (89.4)	226 (88.6)	
All other groups	67 (10.9)	38 (10.6)	29 (11.4)	
**Education level**				2.12 (4), 0.71
GCSE or below	180 (29.3)	108 (30.1)	72 (28.2)	
A level or equivalent	71 (11.6)	45 (12.5)	26 (10.2)	
College qualification	115 (18.7)	62 (17.3)	53 (20.8)	
Degree or higher	213 (34.7)	125 (34.8)	88 (34.5)	
Other	35 (5.7)	19 (5.3)	16 (6.3)	
**Employment status**				3.19 (2), 0.20
Employed (full-time/part-time)	392 (63.8)	234 (65.2)	158 (62.0)	
Unemployed	182 (29.6)	98 (27.3)	84 (32.9)	
Other (studying/retired)	40 (6.5)	27 (7.5)	13 (5.1)	
**Marital status**				2.89 (2), 0.24
Single	129 (21.0)	67 (18.7)	62 (24.3)	
Married/living as married	413 (67.3)	249 (69.4)	164 (64.3)	
Widowed/divorced/separated	72 (11.7)	43 (12.0)	29 (11.4)	
**Parent/carer role**				0.62 (0.45), 0.43
Yes	387 (63.0)	221 (61.6)	166 (65.1)	
No	222 (36.2)	134 (37.3)	88 (34.5)	
**Social status**				7.93 (4), 0.09
AB (highest)	134 (21.8)	90 (25.1)	44 (17.3)	
C1	157 (25.6)	88 (24.5)	69 (27.1)	
C2	142 (23.1)	84 (23.4)	58 (22.7)	
D	93 (15.1)	54 (15.0)	39 (15.3)	
E (lowest)	88 (14.3)	43 (12.0)	45 (17.6)	
**Booking history (Yes/No)**				
Phoned the practice	545 (88.8)	316 (88.0)	229 (89.8)	0.47 (1), 0.49
At reception (in person)	240 (39.1)	145 (40.4)	95 (37.3)	0.62 (1), 0.43
24-hr automated service	23 (3.7)	14 (3.9)	9 (3.5)	0.06 (1), 0.81
Text-message	7 (1.1)	4 (1.1)	3 (1.2)	0.01 (1), 0.94
Website	85 (13.8)	60 (16.7)	25 (9.8)	5.97 (1), <.05
Smartphone app	23 (3.7)	15 (4.2)	8 (3.1)	0.45 (1), 0.50
**Phone ownership**				0.72 (2), 0.70
Smartphone	533 (86.8)	315 (87.7)	218 (85.5)	
Non-smartphone mobile	67 (10.9)	36 (10.0)	31 (12.2)	
No phone	14 (2.3)	8 (2.2)	6 (2.4)	

For peer review only

For peer review only - http://bmjopen.bmj.com/site/about/guidelines.xhtml

Table 2:

Practical barriers to appointment booking and booking characteristics considered to be important (n=614)

	All *(n=614)* N (%)	'Maintainers' *(n=359)* N (%)	'Intenders' *(n=255)* N (%)	OR for being an 'intender' (95% CI)
**Practical barriers to booking screening (% agree/strongly agree)**				
It is (*not*) easy for me to find time to read a letter like this	25 (4.1)	15 (4.2)	10 (3.9)	0.94 (0.41-2.12)
I might forget to book an appointment after reading this letter	187 (30.5)	76 (21.2)	111 (43.5)	2.87 (2.01-4.09)**
It is difficult for me to call my GP practice during their opening hours	192 (31.3)	108 (30.1)	84 (32.9)	1.14 (0.81-1.61)
I (*do not*) have access to a telephone/mobile with phone credit/minutes to call my GP practice	13 (2.1)	8 (2.2)	5 (2.0)	0.88 (0.28-2.71)
I would (*not*) find it easy to find the phone number for my GP practice to contact them	19 (3.1)	11 (3.1)	8 (3.1)	1.01 (0.41-2.59)
I find it difficult to get through to a receptionist when I phone my GP practice	306 (49.8)	177 (49.3)	129 (50.6)	1.05 (0.76-1.45)
**Booking attributes (% saying quite/very important)**				
Ease of booking	519 (84.5)	305 (85.0)	214 (83.9)	0.92 (0.59-1.44)
Choice of appointments	486 (79.2)	280 (78.0)	206 (80.8)	1.19 (0.83-1.77)
Being able to change an appointment after booking	474 (77.2)	274 (76.3)	200 (78.4)	1.13 (0.77-1.66)
How long it takes to book appointment	424 (69.1)	235 (65.5)	189 (74.1)	1.51 (1.06-2.15)*
Waiting time for next available appointment	428 (69.7)	245 (68.2)	183 (71.8)	1.18 (0.83-1.68)
Privacy when booking appointment	410 (66.8)	230 (64.1)	180 (70.6)	1.35 (0.95-1.90)
Being able to talk with a healthcare professional when booking	345 (56.2)	195 (54.3)	150 (58.8)	1.20 (0.87-1.66)
Being able to book an appointment when the GP practice is shut	284 (46.3)	173 (48.2)	111 (43.5)	0.83 (0.60-1.15)[a]
Cost of making booking (i.e. phone credit)	166 (27.0)	94 (26.2)	72 (28.2)	1.11 (0.77-1.59)

Note. OR= odds ratio; CI= confidence interval; *p<0.05, **p<0.001, [a]30% missing data for this variable

For peer review only

Table 3:

Univariable logistic regression models of predictors of the acceptability of cervical screening invitation modalities

	Posted letter (n=598)		Text-message (n=597)		Email (n=592)		Mobile phone call (n=598)		Landline phone call (n=576)	
	%	OR (95% CI)	%	OR (95% CI)	%	OR (95% CI)	%	OR (95% CI)	%	OR (95% CI)
**All participants**	92.5	1.00	80.7	1.00	75.2	1.00	75.8	1.00	62.3	1.00
**Age group**										
25-34	94.7	1.00	86.7	1.00	80.9	1.00	82.4	1.00	65.0	1.00
35-44	92.1	0.66 (0.28-1.52)	84.2	0.82 (0.46-1.46)	78.2	0.85 (0.51-1.41)	80.8	0.90 (0.53-1.52)	67.5	1.12 (0.72-1.74)
45-54	87.5	0.40 (0.18-0.89)*	78.7	0.57 (0.31-1.02)	74.1	0.68 (0.40-1.15)	69.1	0.48 (0.28-0.80)*	53.4	0.62 (0.39-0.98)*
55-64	95.9	1.33 (0.41-4.35)	65.6	0.29 (0.16-0.53)***	60.0	0.36 (0.21-0.62)***	62.9	0.36 (0.21-0.63)***	60.4	0.82 (0.49-1.37)
**Social grade**										
AB	91.6	1.00	77.9	1.00	81.3	1.00	64.6	1.00	51.6	1.00
C1	91.2	0.95 (0.41-2.19)	81.8	1.27 (0.71-2.29)	78.2	0.83 (0.46-1.50)	71.6	1.38 (0.83-2.29)	57.0	1.25 (0.77-2.01)
C2	97.2	3.14 (0.97-10.12)	79.4	1.10 (0.62-1.96)	73.0	0.63 (0.35-1.12)	82.4	2.56 (1.46-4.50)**	67.9	1.99 (1.21-3.27)**
D	95.6	1.99 (0.61-6.47)	86.8	1.87 (0.90-3.90)	79.1	0.87 (0.45-1.71)	79.1	2.08 (1.12-3.86)*	67.8	1.98 (1.12-3.49)*
E	85.2	0.53 (0.23-1.24)	79.1	1.07 (0.55-2.09)	60.0	0.35 (0.19-0.64)*	85.1	3.12 (1.56-6.22)**	73.2	2.56 (1.41-4.66)**
**Employment**										
Employed	93.0	1.00	80.3	1.00	77.8	1.00	73.6	1.00	58.2	1.00
Unemployed	89.8	0.66 (0.36-1.24)	82.9	1.19 (0.75-1.90)	70.5	0.68 (0.46-1.02)	84.0	1.89 (1.19-3.00)**	72.0	1.84 (1.25-2.74)**
Other (studying/retired)	100.0	-	75.7	0.77 (0.35-1.69)	69.4	0.65 (0.31-1.37)	59.5	0.53 (0.26-1.06)	59.5	1.05 (0.53-2.09)
**Ethnicity**										
Any white	93.3	1.00	79.6	1.00	73.5	1.00	74.9	1.00	61.4	1.00
All other groups	85.9	0.44 (0.20-0.97)	90.5	2.44 (1.02-5.80)*	88.9	2.88 (1.28-6.47)**	82.8	1.61 (0.82-3.18)	69.8	1.46 (0.83-2.57)
**Caring responsibilities**										
No	91.7	1.00	78.0	1.00	71.8	1.00	68.1	1.00	58.7	1.00
Yes	92.9	1.19 (0.64-2.22)	82.2	1.30 (0.86-1.97)	77.0	1.31 (0.90-1.92)	80.1	1.88 (1.29-2.76)**	64.3	1.27 (0.90-1.80)
**Screening status**										
Intender	91.1	1.00	82.2	1.00	75.1	1.00	76.5	1.00	61.9	1.00
Maintainer	93.4	1.38 (0.75-2.54)	79.7	0.85 (0.56-1.29)	75.2	1.01 (0.69-1.47)	75.2	0.93 (0.64-1.36)	62.6	1.03 (0.73-1.45)
**Practical barriers**										
0 barriers	94.0	1.00	81.0	1.00	73.1	1.00	77.6	1.00	60.7	1.00
1 barrier	94.1	1.02 (0.43-2.42)	79.1	0.89 (0.54-1.48)	73.7	1.03 (0.64-1.64)	75.9	0.91 (0.56-1.48)	60.4	0.99 (0.65-1.51)
2 barriers	92.5	0.79 (0.33-1.87)	81.6	1.04 (0.60-1.82)	77.2	1.25 (0.75-2.08)	73.6	0.81 (0.49-1.34)	65.0	1.20 (0.76-1.91)
3 or more barriers	84.8	0.36 (0.15-0.84)*	82.3	1.09 (0.55-2.16)	79.7	1.45 (0.77-2.75)	75.0	0.87 (0.47-1.60)	65.8	1.25 (0.71-2.19)

Note. Reference group: 'unacceptable/ambivalent'. OR= unadjusted odds ratio; CI= confidence interval; *p<0.05, **p<0.01, ***p<0.05

For peer review only

Table 4

Univariable logistic regression models of predictors of phone-based booking preferences

	Calling the GP (n=596)		Calling a 24-hour automated service (n=590)		Requesting a call-back (n=593)	
	% likely to book by..	OR (95% CI)	% likely to book by..	OR (95% CI)	% likely to book by..	OR (95% CI)
**All participants**	92.3		53.7		59.0	
**Age group**						
25-34	93.0	1.00	63.2	1.00	61.0	1.00
35-44	92.7	0.94 (0.42-2.09)	54.8	0.71 (0.46-1.07)	64.2	1.15 (0.75-1.76)
45-54	89.7	0.65 (0.30-1.43)	45.9	0.49 (0.31-0.78)**	48.9	0.61 (0.39-0.96)*
55-64	93.8	1.12 (0.41-3.05)	44.2	0.46 (0.28-0.76)**	60.0	0.96 (0.58-1.59)
**Social grade**						
AB	91.5	1.00	51.5	1.00	55.4	1.00
C1	91.8	1.04 (0.44-2.44)	53.1	1.07 (0.33-1.71)	52.7	0.90 (0.56-1.45)
C2	93.6	1.36 (0.54-3.39)	58.9	1.35 (0.83-2.18)	60.3	1.22 (0.75-1.98)
D	94.5	1.59 (0.53-4.74)	54.4	1.12 (0.66-1.93)	65.6	1.53 (0.88-2.67)
E	89.7	0.80 (0.32-2.02)	48.8	0.90 (0.52-1.55)	66.3	1.58 (0.90-2.79)
**Employment**						
Employed	91.7	1.00	51.3	1.00	57.3	1.00
Unemployed	92.6	1.14 (0.58-2.23)	52.3	0.92 (0.64-1.32)	63.6	1.30 (0.91-1.88)
Other (studying/retired)	97.2	3.18 (0.42-23.99)	54.3	1.00 (0.50-2.00)	54.3	0.88 (0.44-1.77)
**Ethnicity**						
Any white	92.3	1.00	52.3	1.00	58.2	1.00
All other groups	92.2	0.99 (0.38-2.59)	65.6	1.74 (1.01-3.00)	65.6	1.37 (0.80-2.36)
**Caring responsibilities**						
No	93	1.00	53.3	1.00	51.4	1.00
Yes	91.9	0.85 (0.45-1.62)	54.0	1.03 (0.73-1.44)*	63.3	1.63 (1.16-2.29)**
**Screening status**						
Intender	91.1	1.00	56.1	1.00	65.7	1.00
Maintainer	93.1	1.33 (0.73-2.44)	52.0	0.85 (0.61-1.18)	54.3	0.62 (0.44-0.90)**
**Practical barriers**						
0 barriers	93.4	1.00	50.9	1.00	57.2	1.00
1 barrier	93.6	1.04 (0.46-2.38)	48.4	0.92 (0.61-1.38)	53.7	0.87 (0.58-1.31)
2 barriers	93.9	1.09 (0.45-2.66)	59.0	1.41 (0.91-2.19)	64.8	1.38 (0.88-2.16)
3 or more barriers cited	83.8	0.37 (0.16-0.84)*	64.1	1.75 (1.01-3.02)*	65.0	1.39 (0.80-2.40)

Note. Reference group: 'not likely to use/ambivalent'. OR= unadjusted odds ratio; CI= confidence interval; *p<0.05, **p<0.01, ***p<0.001

For peer review only - http://bmjopen.bmj.com/site/about/guidelines.xhtml

Table 5

Univariable logistic regression models of predictors of online booking preferences

	Booking on a website using a desktop/laptop (n=589)		Booking on a website using a smartphone [a] (n=-513)		Downloading an app to your smartphone [a] (n=517)	
	% likely to book by..	OR (95% CI)	% likely to book by..	OR (95% CI)	% likely to book by..	OR (95% CI)
**All participants**	60.3		58.8		49.1	
**Age group**						
25-34	71.0	1.00	74.5	1.00	67.6	1.00
35-44	61.9	0.66 (0.43-1.03)	64.8	0.63 (0.40-0.99)*	53.7	0.56 (0.36-0.85)**
45-54	55.2	0.50 (0.32-0.80)**	47.0	0.30 (0.19-0.49)***	36.3	0.27 (0.17-0.44)***
55-64	43.8	0.32 (0.19-0.53)***	34.0	0.18 (0.10-0.30)***	22.9	0.14 (0.08-0.25)***
**Social grade**						
AB	72.3	1.00	70.0	1.00	53.1	1.00
C1	61.1	0.60 (0.36-1.00)	63.9	0.76 (0.46-1.26)	53.4	1.01 (0.63-1.63)
C2	59.3	0.56 (0.34-0.93)*	54.3	0.51 (0.31-0.84)**	48.9	0.85 (0.53-1.37)
D	58.2	0.53 (0.30-0.94)*	54.9	0.52 (0.30-0.91)*	47.3	0.79 (0.46-1.36)
E	44.0	0.30 (0.17-0.54)***	44.7	0.35 (0.20-0.61)***	36.6	0.53 (0.31-0.93)*
**Employment**						
Employed	64.5	1.00	63.7	1.00	53.5	1.00
Unemployed	52.6	0.61 (0.43-0.88)**	51.7	0.61 (0.43-0.88)**	44.8	0.71 (0.49-1.01)
Other (studying/retired)	52.8	0.62 (0.31-1.22)	41.2	0.40 (0.20-0.82)*	22.2	0.25 (0.11-0.56)**
**Ethnicity**						
Any white	59.7	1.00	57.7	1.00	48.4	1.00
All other groups	65.1	1.26 (0.73-2.17)	68.3	1.58 (0.90-2.75)	54.7	1.29 (0.77-2.17)
**Caring responsibilities**						
No	60.6	1.00	54.2	1.00	42.5	1.00
Yes	60.1	0.98 (0.70-1.38)	61.4	1.34 (0.96-1.89)	52.8	1.51 (1.08-2.12)*
**Screening status**						
Intender	59.6	1.00	59.2	1.00	52.8	1.00
Maintainer	60.8	1.05 (0.75-1.47)	58.6	0.98 (0.70-1.36)	46.4	0.77 (0.56-1.07)
**Practical barriers**						
0 barriers	50.8	1.00	48.9	1.00	39	1.00
1 barrier	60.0	1.45 (0.96-2.20)	55.4	1.30 (0.86-1.96)	45.2	1.29 (0.85-1.95)
2 barriers	67.4	2.00 (1.27-3.14)**	68.1	2.23 (1.41-3.52)**	58.3	2.19 (1.40-3.42)**
3 or more barriers	69.6	2.22 (1.27-3.89)**	73.1	2.84 (1.59-5.07)***	64.6	2.85 (1.65-4.93)***

Note. Reference group: 'not likely to use/ambivalent'. OR= unadjusted odds ratio; CI= confidence interval; *p<0.05, **p<0.01, ***p<0.001,
[a] participants with no smartphone removed from analyses (*n* = 81)

For peer review only

Contributorship statement

MR (Conceptualisation; Data analysis; Project administration; Writing – original draft; Writing – review & editing)

JW (Conceptualisation; Supervision; Writing – review & editing)

LM (Conceptualisation; Data analysis; Supervision; Writing – review & editing)

All authors approved the final manuscript as submitted.

Competing interests

The authors have no competing interests to declare.

Funding

MR, JW and LM are supported by a Cancer Research UK Career Development Fellowship awarded to JW (C7492/A17219).

Data sharing statement

Data used and analysed in the study are available from the corresponding author on request (l.marlow@ucl.ac.uk).

The Corresponding Author has the right to grant on behalf of all authors and does grant on behalf of all authors, a worldwide licence to the Publishers and its licensees in perpetuity, in all forms, formats and media (whether known now or created in the future), to i) publish, reproduce, distribute, display and store the Contribution, ii) translate the Contribution into other languages, create adaptations, reprints, include within collections and create summaries, extracts and/or, abstracts of the Contribution, iii) create any other derivative work(s) based on the Contribution, iv) to exploit all subsidiary rights in the Contribution, v) the inclusion of electronic links from the Contribution to third party material where-ever it may be located; and, vi) licence any third party to do any or all of the above.

For peer review only

References

1. NHS. Cervical Screening Programme, England 2016-17, National Statistics. In: Screening & Immunisations Team ND, (ed.). 2017.
2. Landy R, Pesola F, Castanon A, et al. Impact of cervical screening on cervical cancer mortality: estimation using stage-specific results from a nested case-control study. *British journal of cancer* 2016; 115: 1140-1146. 2016/10/26. DOI: 10.1038/bjc.2016.290.
3. Waller J, Jackowska M, Marlow L, et al. Exploring age differences in reasons for nonattendance for cervical screening: a qualitative study. *BJOG : an international journal of obstetrics and gynaecology* 2012; 119: 26-32. 2011/06/15. DOI: 10.1111/j.1471-0528.2011.03030.x.
4. Hope KA, Moss E, Redman CWE, et al. Psycho-social influences upon older women's decision to attend cervical screening: A review of current evidence. *Prev Med* 2017; 101: 60-66. 2017/05/16. DOI: 10.1016/j.ypmed.2017.05.002.
5. Douglas E, Waller J, Duffy SW, et al. Socioeconomic inequalities in breast and cervical screening coverage in England: are we closing the gap? *Journal of medical screening* 2016; 23: 98-103. 2015/09/18. DOI: 10.1177/0969141315600192.
6. Spencer AM, Roberts SA, Brabin L, et al. Sociodemographic factors predicting mother's cervical screening and daughter's HPV vaccination uptake. *Journal of epidemiology and community health* 2014; 68: 571-577. 2014/02/26. DOI: 10.1136/jech-2013-202629.
7. Sutton S and Rutherford C. Sociodemographic and attitudinal correlates of cervical screening uptake in a national sample of women in Britain. *Social Science & Medicine* 2005; 61: 2460-2465. DOI: https://doi.org/10.1016/j.socscimed.2005.07.017.
8. Young B, Bedford L, Kendrick D, et al. Factors influencing the decision to attend screening for cancer in the UK: a meta-ethnography of qualitative research. *Journal of public health (Oxford, England)* 2018; 40: 315-339. 2017/05/10. DOI: 10.1093/pubmed/fdx026.
9. Marlow LAV, Ferrer RA, Chorley AJ, et al. Variation in health beliefs across different types of cervical screening non-participants. *Preventive Medicine* 2018; 111: 204-209. DOI: https://doi.org/10.1016/j.ypmed.2018.03.014.
10. Waller J, Bartoszek M, Marlow L, et al. Barriers to cervical cancer screening attendance in England: a population-based survey. *Journal of medical screening* 2009; 16: 199-204. 2010/01/08. DOI: 10.1258/jms.2009.009073.
11. Marlow LAV, Chorley AJ, Haddrell J, et al. Understanding the heterogeneity of cervical cancer screening non-participants: Data from a national sample of British women. *European journal of cancer (Oxford, England : 1990)* 2017; 80: 30-38. 2017/05/24. DOI: 10.1016/j.ejca.2017.04.017.
12. Sheeran P and Conner M. Improving the translation of intentions into health actions: The role of motivational coherence. *Health psychology : official journal of the Division of Health Psychology, American Psychological Association* 2017; 36: 1065-1073. 2017/08/22. DOI: 10.1037/hea0000553.
13. Sheeran P. Intention—Behavior Relations: A Conceptual and Empirical Review. *European review of social psychology* 2002; 12: 1-36. DOI: 10.1080/14792772143000003.
14. Weinstein ND. The precaution adoption process. *Health psychology : official journal of the Division of Health Psychology, American Psychological Association* 1988; 7: 355-386. 1988/01/01.
15. Sheeran P and Webb T. The Intention–Behavior Gap. *Social and Personality Psychology Compass* 2016; 10: 503-518. DOI: doi:10.1111/spc3.12265.
16. Everett T, Bryant A, Griffin MF, et al. Interventions targeted at women to encourage the uptake of cervical screening. *The Cochrane database of systematic reviews* 2011: CD002834. 2011/05/13. DOI: 10.1002/14651858.CD002834.pub2.
17. McDowell I, Newell C and Rosser W. Computerized reminders to encourage cervical screening in family practice. *The Journal of family practice* 1989; 28: 420-424. 1989/04/01.
18. Binstock MA, Geiger AM, Hackett JR, et al. Pap smear outreach: a randomized controlled trial in an HMO. *American journal of preventive medicine* 1997; 13: 425-426. 1998/01/07.

For peer review only - http://bmjopen.bmj.com/site/about/guidelines.xhtml

19. Duffy SW, Myles JP, Maroni R, et al. Rapid review of evaluation of interventions to improve participation in cancer screening services. *Journal of medical screening* 2017; 24: 127-145. 2016/10/19. DOI: 10.1177/0969141316664757.
20. Kitchener HC, Gittins M, Rivero-Arias O, et al. A cluster randomised trial of strategies to increase cervical screening uptake at first invitation (STRATEGIC). *Health technology assessment (Winchester, England)* 2016; 20: 1-138. 2016/09/17. DOI: 10.3310/hta20680.
21. Ipsos. Social grade: a classification tool. 2009.
22. Sheeran P and Orbell S. Using implementation intentions to increase attendance for cervical cancer screening. *Health Psychology* 2000; 19: 283.
23. Kerrison RS, Shukla H, Cunningham D, et al. Text-message reminders increase uptake of routine breast screening appointments: a randomised controlled trial in a hard-to-reach population. *British journal of cancer* 2015; 112: 1005-1010. 2015/02/11. DOI: 10.1038/bjc.2015.36.
24. Chorley AJ, Marlow LA, Forster AS, et al. Experiences of cervical screening and barriers to participation in the context of an organised programme: a systematic review and thematic synthesis. *Psycho-oncology* 2017; 26: 161-172. 2016/04/14. DOI: 10.1002/pon.4126.
25. Quinn M, Babb P, Jones J, et al. Effect of screening on incidence of and mortality from cancer of cervix in England: evaluation based on routinely collected statistics. *BMJ (Clinical research ed)* 1999; 318: 904-908. 1999/04/02.
26. ONS. Labour Force Survey. Office for National Statistics, 2011.
27. GPPS. GP Patient Survey National results and trends. 2017.
28. MORI I. The GP Patient Survey 2018. Ipsos MORI, 2018.
29. Einstein GO, McDaniel MA, Williford CL, et al. Forgetting of intentions in demanding situations is rapid. *Journal of experimental psychology Applied* 2003; 9: 147-162. 2003/10/23. DOI: 10.1037/1076-898x.9.3.147.
30. ONS. Ethnicity and National Identity in England and Wales: 2011. In: Statistics OfN, (ed.). 2011.
31. Johnson HC, Lafferty EI, Eggo RM, et al. Effect of HPV vaccination and cervical cancer screening in England by ethnicity: a modelling study. *The Lancet Public health* 2018; 3: e44-e51. 2018/01/09. DOI: 10.1016/s2468-2667(17)30238-4.
32. Howard M, Agarwal G and Lytwyn A. Accuracy of self-reports of Pap and mammography screening compared to medical record: a meta-analysis. *Cancer Causes & Control* 2008; 20: 1. journal article. DOI: 10.1007/s10552-008-9228-4.
33. Rauscher GH, Johnson TP, Cho YI, et al. Accuracy of Self-Reported Cancer-Screening Histories: A Meta-analysis. *Cancer Epidemiology Biomarkers & Prevention* 2008; 17: 748-757. DOI: 10.1158/1055-9965.epi-07-2629.

For peer review only

For peer review only - http://bmjopen.bmj.com/site/about/guidelines.xhtml

Online Supplement 1: Could changing invitation and booking processes help women translate their cervical screening intentions into action? A population-based survey of women's preferences in Great Britain.

(Mairead Ryan, Jo Waller and Laura Marlow)

Questionnaire

Have you ever been diagnosed with cervical cancer?

1 Yes
2 No

The next few questions in this section are about cervical screening, also known as a smear or a Pap test. The NHS Cervical Screening Programme invites women in England for a cervical screening, smear or Pap test every 3 years from age 25 to age 49 and every 5 years from age 50 to age 64. Which of these statements describes whether you have had a cervical screening? If you have had a cervical screening and can't remember when, please give your best estimate.

1 I have had a test within the last 3 years > INCLUDE (1)
2 My last test was 3 to 5 years ago > INCLUDE (2)
3 My last test was more than 5 years ago > INCLUDE (3)
4 I have never been invited to have a test > EXCLUDE
5 I have been invited but have never had a test > INCLUDE (4)
6 I have had a hysterectomy so I don't need to have tests > EXCLUDE
7 I have never heard of cervical screening > EXCLUDE

Will you go for cervical screening when next invited?

1 Definitely not > EXCLUDE
2 Probably not > EXCLUDE
3 Yes, probably > INCLUDE (a)
4 Yes, definitely > INCLUDE (a)

NB: Participants were categorised as follows based on responses to the above questions:

If answered 1 and a = maintainer

If 25-49 years and answered 2 and a = intender

If 50-64 and answered 2 and a = maintainer

If answered 3 or 4 and a = intender

On the next screen will be an invitation letter that the NHS sends to women to invite them to book a cervical screening appointment. Most women book cervical screening appointments at their GP practice. I would like you to imagine you received this letter in the post. Please read the letter and afterwards you will be asked some questions about your response to the letter.

* Picture of NHS screening letter shown to participant

For peer review only

I will now read a number of statements relating to the cervical screening letter you've just read. After each statement, please state the extent to which you agree, on a scale from 'strongly disagree 'to 'strongly agree'.

How much do you agree or disagree with this statement?
It is easy for me to find time to read a letter like this.

1 Strongly disagree
2 Disagree
3 Neither disagree or agree
4 Agree
5 Strongly agree

How much do you agree or disagree with this statement?
I might forget to book an appointment after reading this letter.

1 Strongly disagree
2 Disagree
3 Neither disagree or agree
4 Agree
5 Strongly agree

How much do you agree or disagree with this statement?
It is difficult for me to call my GP practice during their opening hours.
*GP opening hours provided if necessary: "Opening hours are generally between 8.00am to 6.30pm Monday to Friday"

1 Strongly disagree
2 Disagree
3 Neither disagree or agree
4 Agree
5 Strongly agree

How much do you agree or disagree with this statement?
I have access to a telephone/mobile with phone credit/minutes to call my GP practice.

1 Strongly disagree
2 Disagree
3 Neither disagree or agree
4 Agree
5 Strongly agree

How much do you agree or disagree with this statement?
It would be easy for me to find the phone number for my GP practice to contact them.

1 Strongly disagree
2 Disagree
3 Neither disagree or agree
4 Agree
5 Strongly agree

For peer review only

How much do you agree or disagree with this statement?
I find it takes too long to get through to a receptionist when I phone my GP practice.

1 Strongly disagree
2 Disagree
3 Neither disagree or agree
4 Agree
5 Strongly agree

We are interested in what is important to you in terms of booking a cervical screening appointment. For the following statements I read out, please state the extent to which you think each factor is important to you, on a scale from 'very unimportant' to 'very important' when booking an appointment at your GP practice.

How important is this when booking a cervical screening appointment at your GP practice?
Ease of booking

1 Very unimportant
2 Quite unimportant
3 Neither unimportant or important
4 Quite important
5 Very important

How important is this when booking a cervical screening appointment at your GP practice?
Cost of making booking (i.e. phone credit)

1 Very unimportant
2 Quite unimportant
3 Neither unimportant or important
4 Quite important
5 Very important

How important is this when booking a cervical screening appointment at your GP practice?
Choice of appointment times

1 Very unimportant
2 Quite unimportant
3 Neither unimportant or important
4 Quite important
5 Very important

How important is this when booking a cervical screening appointment at your GP practice?
Being able to change an appointment time/day after booking it

1 Very unimportant
2 Quite unimportant
3 Neither unimportant or important
4 Quite important
5 Very important

For peer review only

How important is this when booking a cervical screening appointment at your GP practice?
Privacy when booking an appointment

1 Very unimportant
2 Quite unimportant
3 Neither unimportant or important
4 Quite important
5 Very important

How important is this when booking a cervical screening appointment at your GP practice?
How long it takes to book an appointment

1 Very unimportant
2 Quite unimportant
3 Neither unimportant or important
4 Quite important
5 Very important

How important is this when booking a cervical screening appointment at your GP practice?
Being able to talk with a healthcare professional when booking (e.g. to ask questions about the screening before attending)

1 Very unimportant
2 Quite unimportant
3 Neither unimportant or important
4 Quite important
5 Very important

How important is this when booking a cervical screening appointment at your GP practice?
Time to the next available appointment (e.g. next available appointment isn't for two weeks)

1 Very unimportant
2 Quite unimportant
3 Neither unimportant or important
4 Quite important
5 Very important

How important is this when booking a cervical screening appointment at your GP practice?
Being able to book an appointment when the GP practice is shut (e.g. online booking)
*GP opening hours provided if necessary: "Opening hours are generally between 8.00am to 6.30pm Monday to Friday"

1 Very unimportant
2 Quite unimportant
3 Neither unimportant or important
4 Quite important
5 Very important

Again thinking about the letter you read which is sent in the post to invite women to book a cervical screening appointment. We are interested in different forms of communication to invite women to book a cervical screening appointment.

Please state the extent to which you think the following forms of communication are acceptable, on a scale from 'very unacceptable' to 'very acceptable'.

For peer review only

How acceptable is this form of communication when being invited to book a cervical screening appointment?

Posted letter

1 Very unacceptable
2 Quite unacceptable
3 Neither unacceptable or acceptable
4 Quite acceptable
5 Very acceptable

* If participant responded 'Quite unacceptable' or 'Very unacceptable', participant subsequently asked: Please can you tell me why you would not find receiving an invitation for a cervical screening appointment by posted letter acceptable?

How acceptable is this form of communication when being invited to book a cervical screening appointment?

Text message

1 Very unacceptable
2 Quite unacceptable
3 Neither unacceptable or acceptable
4 Quite acceptable
5 Very acceptable

* If participant responded 'Quite unacceptable' or 'Very unacceptable', participant subsequently asked: Please can you tell me why you would not find receiving an invitation for a cervical screening appointment by text message acceptable?

How acceptable is this form of communication when being invited to book a cervical screening appointment?

Email

1 Very unacceptable
2 Quite unacceptable
3 Neither unacceptable or acceptable
4 Quite acceptable
5 Very acceptable

* If participant responded 'Quite unacceptable' or 'Very unacceptable', participant subsequently asked: Please can you tell me why you would not find receiving an invitation for a cervical screening appointment by email acceptable?

For peer review only

How acceptable is this form of communication when being invited to book a cervical screening appointment?
Phone call to your mobile phone

1 Very unacceptable
2 Quite unacceptable
3 Neither unacceptable or acceptable
4 Quite acceptable
5 Very acceptable

* If participant responded 'Quite unacceptable' or 'Very unacceptable', participant subsequently asked: Please can you tell me why you would not find receiving an invitation for a cervical screening appointment by phone call to your mobile phone acceptable?

How acceptable is this form of communication when being invited to book a cervical screening appointment?
Phone call to your house landline

1 Very unacceptable
2 Quite unacceptable
3 Neither unacceptable or acceptable
4 Quite acceptable
5 Very acceptable

* If participant responded 'Quite unacceptable' or 'Very unacceptable', participant subsequently asked: Please can you tell me why you would not find receiving an invitation for a cervical screening appointment by phone call to your house landline acceptable?

Imagine now that different options were available to you to book a cervical screening appointment at your GP practice. Please state the extent to which you are likely to use each of the following methods to book an appointment.

How likely are you to use this method to book a cervical screening appointment at your GP practice?
Calling your GP practice

1 Very unlikely
2 Quite unlikely
3 Neither likely or unlikely
4 Quite likely
5 Very likely

How likely are you to use this method to book a cervical screening appointment at your GP practice?
Calling a 24-hour automated telephone appointment-booking system

1 Very unlikely
2 Quite unlikely
3 Neither likely or unlikely
4 Quite likely
5 Very likely

For peer review only

How likely are you to use this method to book a cervical screening appointment at your GP practice?
Requesting a call-back from your GP practice

1 Very unlikely
2 Quite unlikely
3 Neither likely or unlikely
4 Quite likely
5 Very likely

How likely are you to use this method to book a cervical screening appointment at your GP practice?
Booking on a website using a desktop computer/laptop

1 Very unlikely
2 Quite unlikely
3 Neither likely or unlikely
4 Quite likely
5 Very likely

How likely are you to use this method to book a cervical screening appointment at your GP practice?
Booking on a website using a smartphone

1 Very unlikely
2 Quite unlikely
3 Neither likely or unlikely
4 Quite likely
5 Very likely

How likely are you to use this method to book a cervical screening appointment at your GP practice?
Downloading an app to a smartphone to book an appointment (you could then use the app to book other appointments at your surgery)

1 Very unlikely
2 Quite unlikely
3 Neither likely or unlikely
4 Quite likely
5 Very likely

Which of the following methods have you previously used to book an appointment at your GP practice? This could be an appointment for anything, with a GP or with a nurse.
Please select all that apply.

1 Booked in person (i.e. at the reception desk)
2 Booked by phoning the GP practice
3 Booked using a 24-hour automated telephone appointment-booking system
4 Booked online on a website
5 Booked by text-message
6 Booked using a smartphone app
7 Other
8 Don't know - someone else has always booked my appointments
9 I have never booked an appointment at my GP practice

For peer review only

Do you have a mobile phone?
*Description of smartphone provided if necessary; "A 'smart phone' is a mobile phone that performs many of the functions of a computer, typically having a touchscreen and Internet access"

1 Yes, a smart phone
2 Yes, but it is not a smart phone
3 No, I do not have a mobile phone

For peer review only

Online Supplement 2: Could changing invitation and booking processes help women translate their cervical screening intentions into action? A population-based survey of women's preferences in Great Britain.

(Mairead Ryan, Jo Waller and Laura Marlow)

Survey inclusion flow diagram

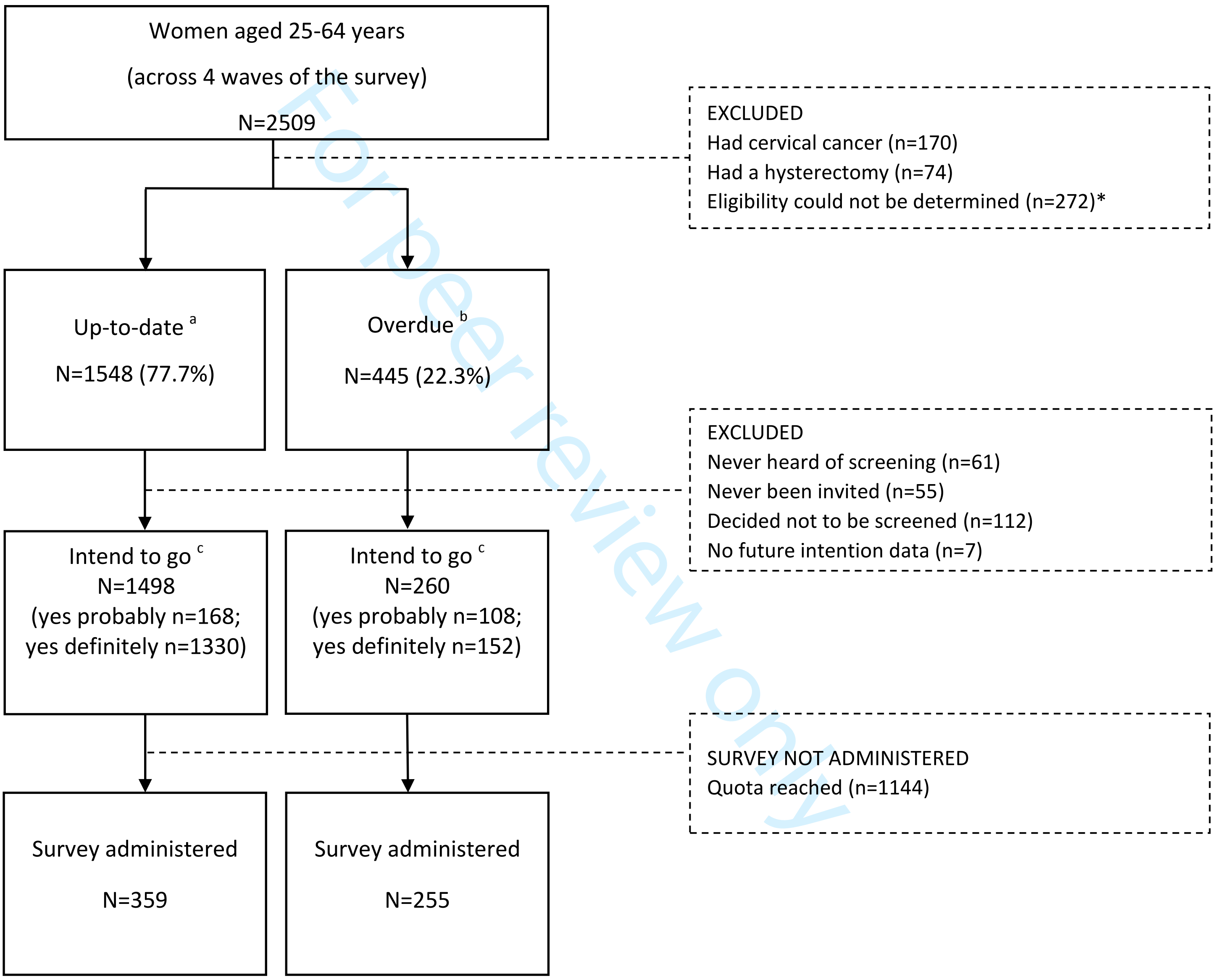

*Women who refused to answer the hysterectomy question (n=177) or screening uptake question (n=95)

[a] Up-to-date: been screened within the last 3 years if 25-64 years or the last 5 years if 50-64 years

[b] Overdue: not been screened within the last 3 years if 25-64 years or the last 5 years if 50-64 years

[c] Responded that they would 'probably' or 'definitely' attend screening when next invited

For peer review only

Online Supplement 3: Could changing invitation and booking processes help women translate their cervical screening intentions into action? A population-based survey of women's preferences in Great Britain.

(Mairead Ryan, Jo Waller and Laura Marlow)

Table S1:

Descriptives for acceptability of cervical screening invitation modalities (n=614)

	Very unacceptable/Quite unacceptable N (%)	Neither unacceptable or acceptable N (%)	Quite acceptable/Very acceptable N (%)	Don't know/ not applicable (Excluded) N (%)
Posted letter	14 (2.3)	31 (5.0)	90.1 (90.1)	16 (2.6)
Text-message	65 (10.6)	50 (8.1)	482 (78.5)	17 (2.8)
Email	95 (15.5)	52 (8.5)	445 (72.5)	22 (3.6)
Mobile phone call	92 (15.0)	53 (8.6)	453 (73.8)	16 (2.6)
Landline phone call	132 (21.5)	85 (13.8)	359 (58.5)	38 (6.2)

Table S2:

Descriptives for likelihood of using different phone-based and online booking methods (n=614)

	Very unlikely/Quite unlikely N (%)	Neither unlikely or likely N (%)	Quite likely/Very likely N (%)	Don't know/ not applicable (Excluded) N (%)
Calling the GP	21 (3.4)	25 (4.1)	550 (89.6)	18 (2.9)
Calling a 24-hour automated service	182 (29.6)	91 (14.8)	317 (51.6)	24 (3.9)
Requesting a call-back	164 (26.7)	79 (12.9)	350 (57.0)	21 (3.4)
Booking on a website using a desktop/laptop	172 (28.0)	62 (10.1)	355 (57.8)	25 (4.1)
Booking on a website using a smartphone[a]	141 (26.5)	44 (8.3)	328 (61.5)	20 (3.8)
Downloading an app to your smartphone[a]	185 (34.7)	56 (10.5)	276 (51.8)	16 (3.0)

[a] participants with no smartphone removed from analyses (*n* = 81)

For peer review only

Table S3

Multivariable logistic regression models of predictors of the acceptability of cervical screening invitation modalities

	**Posted letter (n=597)**	**Text-message (n=596)**	**Email (n=591)**	**Mobile phone call (n=597)**	**Landline phone call (n=575)**
	OR (95% CI)	OR (95% CI)	OR (95% CI)	OR (95% CI)	OR (95% CI)
**Age group**					
25-34	1.00	1.00	1.00	1.00	1.00
35-44	0.71 (0.30-1.68)	0.79 (0.44-1.44)	0.73 (0.43-1.23)	0.86 (0.50-1.50)	1.16 (0.73-1.83)
45-54	0.40 (0.17-0.92)*	0.60 (0.33-1.09)	0.70 (0.41-1.20)	0.49 (0.29-0.84)**	0.65 (0.41-1.05)
55-64	1.06 (0.31-3.66)	0.28 (0.15-0.55)***	0.36 (0.19-0.66)**	0.48 (0.26-0.87)*	0.93 (0.53-1.62)
**Social grade**					
AB	1.00	1.00	1.00	1.00	1.00
C1	1.00 (0.42-2.36)	1.16 (0.63-2.11)	0.75 (0.41-1.38)	1.25 (0.74-2.11)	1.15 (0.71-1.88)
C2	3.47 (1.05-11.47)*	0.97 (0.53-1.77)	0.57 (0.31-1.03)	2.37 (1.32-4.23)**	1.84 (1.10-3.06)*
D	2.11 (0.63-7.04)	1.71 (0.80-3.65)	0.81 (0.40-1.62)	1.85 (0.97-3.51)	1.83 (1.02-3.27)*
E	0.57 (0.17-1.87)	0.84 (0.36-1.93)	0.30 (0.14-0.65)**	2.69 (1.17-6.16)*	1.93 (0.94-3.97)
**Employment**					
Employed	1.00	1.00	1.00	1.00	1.00
Unemployed	0.90 (0.35-2.30)	1.34 (0.73-2.44)	1.05 (0.62-1.80)	1.27 (0.73-2.23)	1.46 (0.90-2.37)
Other (studying/retired)†	-	-	-	-	-
**Ethnicity**					
White	1.00	1.00	1.00	1.00	1.00
All other groups	0.39 (0.17-0.90)*	2.09 (0.86-5.08)	2.85 (1.24-6.57)*	1.34 (0.67-2.72)	1.31 (0.73-2.36)
**Caring responsibilities**					
No	1.00	1.00	1.00	1.00	1.00
Yes	1.50 (0.76-2.96)	0.95 (0.60-1.51)	1.10 (0.72-1.70)	1.52 (1.00-2.32)	1.14 (0.78-1.67)

Note. Reference group: 'unacceptable/ambivalent'. OR= adjusted odds ratio; CI= confidence interval; *p<0.05, **p<0.01, ***p<0.001; 'screening status' and 'practical barriers' variables not included because not significant in univariable analyses; †category not included due to insufficient cases

For peer review only

Table S4

Multivariable logistic regression models of predictors of phone-based booking preferences

	Calling the GP (n=596)	Calling a 24-hour automated service (n=590)	Requesting a call-back (n=593)
	OR (95% CI)	OR (95% CI)	OR (95% CI)
**Age group**			
25-34	1.00	1.00	1.00
35-44	1.05 (0.47-2.36)	0.69 (0.45-1.06)	1.05 (0.68-1.63)
45-54	0.60 (0.27-1.35)	0.50 (0.31-0.79)**	0.65 (0.41-1.02)
55-64	0.96 (0.34-2.75)	0.46 (0.27-0.78)**	1.30 (0.76-2.22)
**Caring responsibilities**			
No	1.00	1.00	1.00
Yes	0.84 (0.43-1.67)	0.92 (0.64-1.33)	1.74 (1.20-2.52)**
**Screening status**			
Intender	1.00	1.00	1.00
Maintainer	1.31 (0.70-2.43)	0.94 (0.67-1.32)	0.63 (0.45-0.90)*
**Practical barriers**			
0 barriers	1.00	1.00	1.00
1 barrier	1.00 (0.43-2.33)	0.83 (0.54-1.26)	0.73 (0.48-1.13)
2 barriers	1.05 (0.42-2.60)	1.24 (0.79 -1.96)	1.21 (0.76-1.93)
3 or more barriers cited	0.35 (0.15-0.83)*	1.59 (0.91-2.78)	1.17 (0.67-2.07)

Note. Reference group: ‘not likely to use/ambivalent’ OR= adjusted odds ratio; CI= confidence interval; *p<0.05, **p<0.01, ***p<0.001; ‘social grade’, ‘employment’ and ‘ethnicity’ not included because not significant in univariable analyses

For peer review only

Table S5

Multivariable logistic regression models of predictors of online booking preferences

	**Booking on a website using a desktop/laptop (n=589)**	**Booking on a website using a smartphone [a] (n=513)**	**Downloading an app to your smartphone [a] (n=517)**
	OR (95% CI)	OR (95% CI)	OR (95% CI)
**Age group**			
25-34	1.00	1.00	1.00
35-44	0.60 (0.38-0.95)*	0.59 (0.34-0.92)*	0.52 (0.33-0.82)**
45-54	0.52 (0.32-0.85)**	0.36 (0.21-0.61)***	0.35 (0.21-0.58)***
55-64	0.32 (0.18-0.55)***	0.25 (0.13-0.47)***	0.23 (0.12-0.44)***
**Social grade**			
AB	1.00	1.00	1.00
C1	0.59 (0.35-0.99)*	0.67 (0.38-1.18)	0.91 (0.54-1.54)
C2	0.51 (0.30-0.87)*	0.47 (0.26-0.83)*	0.82 (0.47-1.41)
D	0.47 (0.26-0.86)*	0.39 (0.20-0.73)**	0.62 (0.34-1.14)
E	0.34 (0.17-0.68)**	0.41 (0.18-0.94)*	0.76 (0.34-1.69)
**Employment**			
Employed	1.00	1.00	1.00
Unemployed	0.85 (0.54-1.35)	0.84 (0.50-1.41)	0.79 (0.48-1.31)
Other (studying/retired)	0.91 (0.42-1.97)	0.55 (0.22-1.35)	0.35 (0.13-0.91)*
**Practical barriers**			
0 barriers	1.00	1.00	1.00
1 barrier	1.35 (0.87-2.08)	1.18 (0.73-1.91)	1.21 (0.75-1.93)
2 barriers	1.67 (1.04-2.69)*	1.52 (0.90-2.54)	1.66 (1.00-2.73)*
3 or more barriers	2.07 (1.15-3.73)*	2.74 (1.41-5.33)**	2.46 (1.00-2.73)**

Note. Reference group: 'not likely to use/ambivalent' OR= adjusted odds ratio; CI= confidence interval; *p<0.05, **p<0.01, ***p<0.001; [a] participants with no smartphone removed from analyses (*n* = 81); 'ethnicity', 'caring responsibilities' and 'screening status' not included because not significant in univariable analyses

For peer review only

Online Supplement 4: Could changing invitation and booking processes help women translate their cervical screening intentions into action? A population-based survey of women's preferences in Great Britain.

(Mairead Ryan, Jo Waller and Laura Marlow)

Open responses provided for citing invitation method as unacceptable

Invitation mode	Unacceptable (n)	Reasons for being unacceptable
Posted letter	12	Don't open post/might miss the letter/no time to read letter (n=4)<br>Receive letter too late (n=2)<br>Letter could be lost in the post (n=2)<br>Other (n=4)<br>• Would forget (n=1)<br>• Environmental concerns (n=1)<br>• Waste of time (n=1)<br>• No reason provided (n=1)
Text-message	67	Privacy concerns (n=21)<br>Easy to miss it/may not read message (n=9)<br>Reason not provided (i.e. N/A) (n=9)<br>Doesn't have or use mobile (n=7)<br>Impersonal (n=6)<br>Could change number (n=4)<br>Prefer a letter/phone call (n=4)<br>Not reliable source/unprofessional (n=3)<br>Would forget/not act on it (n=2)<br>Other (n=2)<br>• Don't know (n=1)<br>• They can text me but I don't want to text them (n=1)
Email	94	Would be lost in other emails/would not be seen (n=38)<br>No email/doesn't use email/no internet/no computer (n=17)<br>Privacy concerns (n=12)<br>Reason not provided (i.e. N/A) (n=12)<br>Prefer phone or letter (n=5)<br>Would forget/not act on it (n=2)<br>Impersonal/rude (n=2)<br>Other (n=6)<br>• Not timely (n=1)<br>• Intrusive (n=1)<br>• Not normal (n=1)<br>• No reason (n=1)<br>• Not keen (n=1)<br>• Doesn't trust source (n=1)
Mobile	90	Would not be able to pick up/would miss call (n=33)

For peer review only

phone call		Privacy concerns (n=22)<br>Would prefer in writing/a letter (n=10)<br>Reason not provided (i.e. N/A) (n=8)<br>Would not know number – so would not answer call (n=5)<br>No mobile (n=2)<br>Would forget (n=2)<br>Too many phone calls (n=2)<br>Other (n=6)<br>• Don't like idea (n=1)<br>• Talking takes too much time (n=1)<br>• Need time to think (n=1)<br>• Impersonal (n=1)<br>• People change phone number (n=1)<br>• Don't like calls (n=1)
Landline phone call	129	No landline (n=39)<br>Would miss call/out of the house during the day (n=31)<br>Privacy concerns (n=24)<br>No reason provided (i.e. N/A) (n=12)<br>Feels intrusive (n=5)<br>Prefer in writing/letter (n=5)<br>Don't want phone call (n=4)<br>Not reliable source (n=3)<br>Other (n=6)<br>• Impersonal (n=1)<br>• "Better with working" (n=1)<br>• Unnecessary (n=1)<br>• Unknown number (n=1)<br>• Want time to think (n=1)<br>• Doesn't matter either way (n=1)

For peer review only

STROBE 2007 (v4) Statement—Checklist of items that should be included in reports of *cross-sectional studies*

Section/Topic	Item #	Recommendation	Reported on page #
**Title and abstract**	1	(*a*) Indicate the study's design with a commonly used term in the title or the abstract	2
		(*b*) Provide in the abstract an informative and balanced summary of what was done and what was found	2
**Introduction**			
Background/rationale	2	Explain the scientific background and rationale for the investigation being reported	3-4
Objectives	3	State specific objectives, including any prespecified hypotheses	4
**Methods**			
Study design	4	Present key elements of study design early in the paper	4
Setting	5	Describe the setting, locations, and relevant dates, including periods of recruitment, exposure, follow-up, and data collection	4
Participants	6	(*a*) Give the eligibility criteria, and the sources and methods of selection of participants	4-5
Variables	7	Clearly define all outcomes, exposures, predictors, potential confounders, and effect modifiers. Give diagnostic criteria, if applicable	5-6
Data sources/ measurement	8*	For each variable of interest, give sources of data and details of methods of assessment (measurement). Describe comparability of assessment methods if there is more than one group	5-6
Bias	9	Describe any efforts to address potential sources of bias	4
Study size	10	Explain how the study size was arrived at	5
Quantitative variables	11	Explain how quantitative variables were handled in the analyses. If applicable, describe which groupings were chosen and why	6
Statistical methods	12	(*a*) Describe all statistical methods, including those used to control for confounding	6
		(*b*) Describe any methods used to examine subgroups and interactions	6
		(*c*) Explain how missing data were addressed	
		(*d*) If applicable, describe analytical methods taking account of sampling strategy	
		(*e*) Describe any sensitivity analyses	
**Results**			

For peer review only

Participants	13*	(a) Report numbers of individuals at each stage of study—eg numbers potentially eligible, examined for eligibility, confirmed eligible, included in the study, completing follow-up, and analysed	6
		(b) Give reasons for non-participation at each stage	n/a
		(c) Consider use of a flow diagram	
Descriptive data	14*	(a) Give characteristics of study participants (eg demographic, clinical, social) and information on exposures and potential confounders	6-7
		(b) Indicate number of participants with missing data for each variable of interest	
Outcome data	15*	Report numbers of outcome events or summary measures	
Main results	16	(*a*) Give unadjusted estimates and, if applicable, confounder-adjusted estimates and their precision (eg, 95% confidence interval). Make clear which confounders were adjusted for and why they were included	7-8, 12-15
		(*b*) Report category boundaries when continuous variables were categorized	5-6
		(*c*) If relevant, consider translating estimates of relative risk into absolute risk for a meaningful time period	n/a
Other analyses	17	Report other analyses done—eg analyses of subgroups and interactions, and sensitivity analyses	
**Discussion**			
Key results	18	Summarise key results with reference to study objectives	8-10
Limitations	19	Discuss limitations of the study, taking into account sources of potential bias or imprecision. Discuss both direction and magnitude of any potential bias	9-10
Interpretation	20	Give a cautious overall interpretation of results considering objectives, limitations, multiplicity of analyses, results from similar studies, and other relevant evidence	10
Generalisability	21	Discuss the generalisability (external validity) of the study results	9-10
**Other information**			
Funding	22	Give the source of funding and the role of the funders for the present study and, if applicable, for the original study on which the present article is based	1

*Give information separately for cases and controls in case-control studies and, if applicable, for exposed and unexposed groups in cohort and cross-sectional studies.

Note: An Explanation and Elaboration article discusses each checklist item and gives methodological background and published examples of transparent reporting. The STROBE checklist is best used in conjunction with this article (freely available on the Web sites of PLoS Medicine at http://www.plosmedicine.org/, Annals of Internal Medicine at http://www.annals.org/, and Epidemiology at http://www.epidem.com/). Information on the STROBE Initiative is available at www.strobe-statement.org.

For peer review only
